# Synthesis of 7-azabicyclo[4.3.1]decane ring systems from tricarbonyl(tropone)iron via intramolecular Heck reactions

**DOI:** 10.3762/bjoc.19.118

**Published:** 2023-10-23

**Authors:** Aaron H Shoemaker, Elizabeth A Foker, Elena P Uttaro, Sarah K Beitel, Daniel R Griffith

**Affiliations:** 1 Department of Chemistry, Lafayette College, 730 High St., Easton, PA 18042, USA

**Keywords:** alkaloids, azabicycles, Heck reaction, iron complex, tropone

## Abstract

The 7-azabicyclo[4.3.1]decane ring system, common to a number of biologically active alkaloids, was accessed from tropone (via its η^4^-diene complex with Fe(CO)_3_) in a short sequence of steps: 1) nucleophilic amine addition and subsequent Boc-protection, 2) photochemical demetallation of the iron complex, and 3) an intramolecular Heck reaction. Minor modifications to the protocol enabled access to the related 2-azabicyclo[4.4.1]undecane system, albeit in lower yield.

## Introduction

**Scheme 1:**
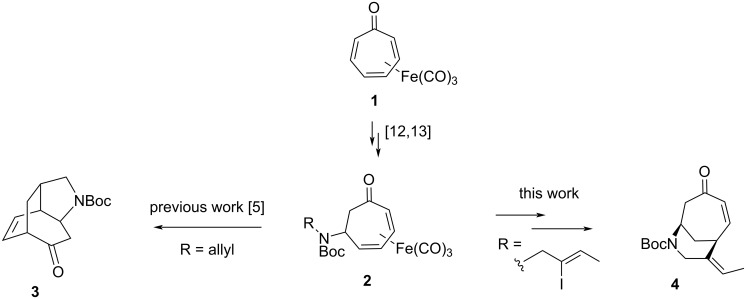
Synthesis of diverse azapolycycles from iron complex **2** derived from tricarbonyl(tropone)iron.

Azapolycycles are embedded within numerous biologically active alkaloids [1] and pharmaceuticals [2]. As such, novel approaches to the synthesis of these motifs have attracted considerable interest. The synthesis of azapolycycles containing a seven-membered carbocyclic ring is particularly challenging in contrast to comparable skeletons containing only five- or six-membered rings [3]. Thus, comparatively few general methods exist for accessing these scaffolds, even though they are found within a number of biologically active alkaloids. We recently demonstrated that the readily available, bench-stable tricarbonyl(tropone)iron complex [4] (**1**, Scheme 1) could serve as a precursor to the previously unreported 2-azatricyclo[4.3.2.0^4,9^]undecane ring system [5] (**3**, Scheme 1). We sought to demonstrate that this iron complex could serve as a common, versatile building block for additional azapolycyclic skeletons.

**Figure 1:**
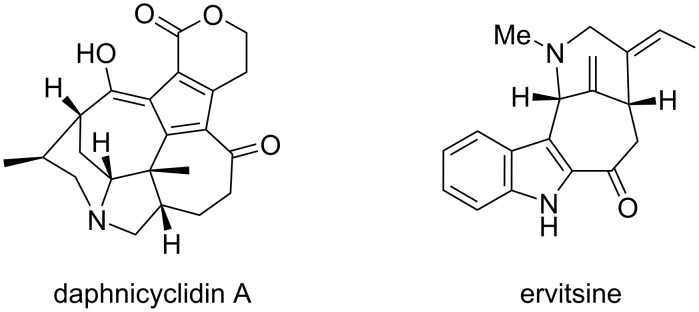
Complex alkaloids containing the 7-azabicyclo[4.3.1]decane ring system.

The 7-azabicyclo[4.3.1]decane ring system is found within several complex alkaloids, including daphnicyclidin A [6–9] and ervitsine [10–11] (Figure 1). We reasoned that, with an appropriately functionalized amine side chain and properly disposed unsaturation on the seven-membered ring, an intramolecular Heck reaction could give rise to skeleton **4** in just a few steps from tricarbonyl(tropone)iron (Scheme 1). Herein, we report our successful efforts to access this bridged bicyclic ring system.

## Results and Discussion

**Scheme 2:**
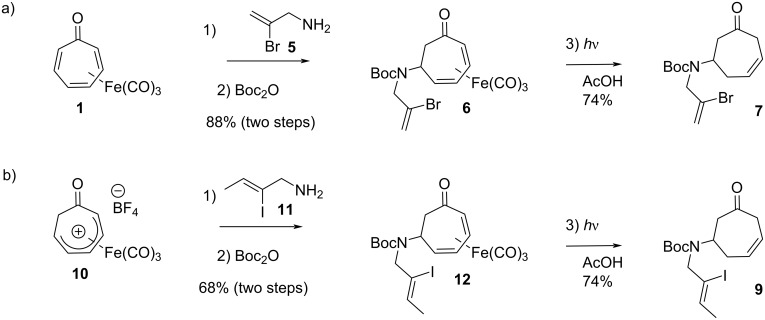
Synthesis of Heck substrates. a) Substrate **7**, reagents and conditions: 1) neat (5 equiv **5**), 24 h; 2) Boc_2_O, NaHCO_3_, EtOH, ultrasound, 1 h, 88% (2 steps); 3) AcOH, *h*ν (360 nm), 6 h, 74%. b) Substrate **9**, reagents and conditions: 1) **11** (2 equiv), EtOAc, 23 °C, 1 h; 2) Boc_2_O, NaHCO_3_, EtOH, ultrasound, 23 °C, 1 h, 68% (2 steps); 3) AcOH, *h*ν (360 nm), 23 °C, 6 h, 74%.

As shown in Scheme 2a, the synthesis of the requisite Heck reaction precursor began with the addition of the known allylic amine **5** to tricarbonyl(tropone)iron, immediately followed by Boc-protection of the crude secondary amine according to our previously described, solvent-free protocol [12–13]. The resulting iron complex **6** was demetallated upon irradiation with UV light [14] to give the deconjugated olefin **7**.

We then hoped to forge the desired bicycle **8** (Table 1) via a 6-*exo-trig* Heck cyclization, drawing on the vast body of knowledge built from many synthetic campaigns towards the *Strychnos* alkaloids [15]. Several combinations of palladium catalyst, base, and other additives were applied to our system (see Table 1, entries 1–4). Reaction conditions such as those deployed to great effect by Rawal [16] (Table 1, entry 2) and Vanderwal [17] (Table 1, entry 3) in the synthesis of other bridged azapolycycles gave poor yields when applied to vinyl bromide **7**. The best result was obtained using the combination of Pd(PPh_3_)_4_, K_2_CO_3_, and proton sponge in refluxing toluene [18–19]. Although this catalyst system proved best among those screened, yields remained modest (44%). X-ray analysis provided confirmation of the structure of bicycle **8** (CCDC No. 2263675).

**Table 1:** Screening of conditions for intramolecular Heck reaction of vinyl halides **7** and **9**.

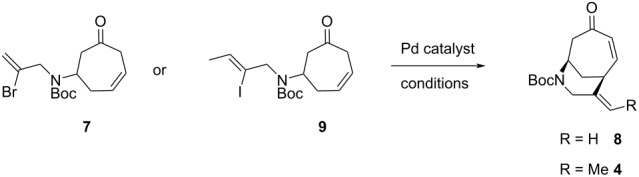			

Entry	Substrate	Conditions	Yield (%)

1	**7**	Pd(OAc)_2_, PPh_3_, Et_3_N, dioxane, 120 °C, 4 h	15
2	**7**	Pd(OAc)_2_, PPh_3_, Et_3_N, MeCN, 80 °C, 2 h	15
3	**7**	Pd(PPh_3_)_4_, PMP, MeCN, 70 °C, 4 h	17
4	**7**	Pd(PPh_3_)_4_, proton sponge, K_2_CO_3_, PhMe, 110 °C, 4.5 h	44
5	**9**	Pd(PPh_3_)_4_, proton sponge, K_2_CO_3_, PhMe, 110 °C, 4.5 h	45
6	**9**	Pd(OAc)_2_, K_2_CO_3_, *n-*Bu_4_NCl, DMF, 70 °C, 4 h	7
7	**9**	Pd(PPh_3_)_4_, K_3_PO_4_, PhOH, Et_3_N, PhMe, 110 °C, 4 h	76
8	**7**	Pd(PPh_3_)_4_, K_3_PO_4_, PhOH, Et_3_N, PhMe, 110 °C, 2 h	42

While searching for methods to improve the yield of our desired azabicycle, we came across the observation of Andrade and Kokkonda that vinylic halides with the same substitution pattern as substrate **7** tend to give poor yields in similar intramolecular Heck reactions [20]. Moreover, it was found that substrates on which the halide is *cis* to an additional methyl substituent (used to forge the ethylidene-substituted polycycle common to many alkaloids) often give superior yields for otherwise identical Heck reactions. Thus, iodoamine **11** (Scheme 2b) was synthesized (see Supporting Information File 1) to evaluate this finding in the context of our system.

Subjection of **9** to the same conditions that proved optimal for vinyl bromide **7** resulted in the formation of the expected product **4**, but with essentially no improvement in yield (Table 1, entry 5). Jeffery conditions [16], which have been shown to work well with similar vinyl iodides, provided a very low yield (entry 6 in Table 1). However, we had better success when we adapted the conditions developed by Bennasar, which elevated the yield to 76% (Table 1, entry 7) [10]. It has been postulated [21] that phenoxide is capable of stabilizing one or more of the intermediate Pd complexes, which may account for the higher yield. Interestingly, applying these conditions to the vinyl bromide **7** resulted in no improvement over the previously optimized conditions (Table 1, entry 8).

Having established an efficient route to our desired azabicycle from tropone, we sought to employ additional amine nucleophiles bearing pendant vinyl iodides to access several analogs of product **4** (see Supporting Information File 1 for amine syntheses). These amines were each carried through the protocols outlined in Scheme 2 to arrive at the deconjugated olefin substrates shown in Table 2. The cinnamylamine derivative **13** underwent the Heck cyclization in 50% yield, while the prenylamine derivative **15** proceeded to give cyclized product **16** in 75% yield. These results suggest that these Heck cyclizations are quite sensitive to the identity of the alkene substituent that is *cis* to the halogen. We were also interested in engaging vinyl iodide **17** in a 7-*exo*-trig cyclization to form **18**. *Z*-Iodoalkene **17** appeared to react cleanly according to TLC analysis, but the isolated yield of the intramolecular Heck product was low, perhaps due to instability of one of the intermediate palladium complexes and/or a slow olefin insertion step. Moreover, the product was obtained as an inseparable mixture of the allylic carbamate **18** and the isomeric enecarbamate **19** (the precise product ratio was variable across several trials, since the ratio of the two compounds was observed to change upon subjection of the crude product mixture to column chromatography).

**Table 2:** Intramolecular Heck reactions of various substituted vinyl halides.^a^

Substrate	Product	Yield (%)

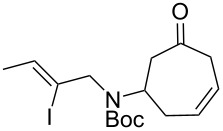 **9**	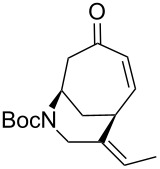 **4**	76
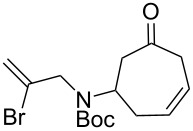 **7**	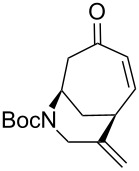 **8**	42
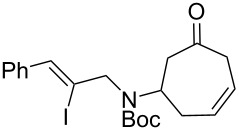 **13**	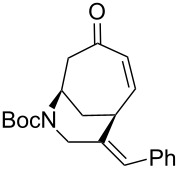 **14**	50
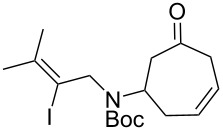 **15**	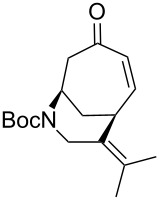 **16**	75
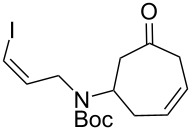 **17**	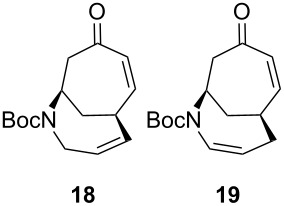	15^b^

^a^Reaction conditions: Pd(PPh_3_)_4_, K_3_PO_4_, PhOH, Et_3_N, PhMe, 110 °C; ^b^combined yield of **18** and **19**.

## Conclusion

In conclusion, we have shown that bridged azabicycles common to a number of alkaloid natural products can be accessed from commercially available tropone in as little as five steps: 1) formation of tricarbonyl(tropone)iron, 2) *aza*-Michael addition, 3) amine protection, 4) photodemetallation, and 5) intramolecular Heck reaction (two steps – *aza*-Michael addition and amine protection – can potentially take place in one pot). We have shown that this protocol can be applied to the synthesis of several analogs bearing different substitution patterns on the alkene. The structural diversity that can be readily obtained utilizing this chemistry underscores the versatility of tropone as a synthetic building block for accessing functionalized azapolycycles containing seven-membered rings.

## Supporting Information

File 1 Experimental procedures for all new compounds and summary of X-ray structure data for compound **8**.

File 2 Copies of ^1^H and ^13^C NMR spectra of all purified novel compounds.

File 3 Chrystallographic information file (cif) of X-ray structure for compound **8**.
