# Supplementary material for: Synthesis of 7-azabicyclo[4.3.1]decane ring systems from tricarbonyl(tropone)iron via intramolecular Heck reactions
Source: Beilstein J Org Chem. 2023 Oct 23;19:1615–9. doi: 10.3762/bjoc.19.118 (PMC10616702; doi:10.3762/bjoc.19.118)
Supplement: File 1 — Experimental procedures for all new compounds and summary of X-ray structure data for compound 8. [file Beilstein_J_Org_Chem-19-1615-s001.pdf]

BEILSTEIN JOURNAL OF ORGANIC CHEMISTRY

Supporting Information

for

Synthesis of 7-azabicyclo[4.3.1]decane ring systems from tricarbonyl(tropone)iron via intramolecular Heck reactions

Aaron H. Shoemaker, Elizabeth A. Foker, Elena P. Uttaro, Sarah K. Beitel
and Daniel R. Griffith

Beilstein J. Org. Chem. **2023,** *19,* 1615–1619. doi:10.3762/bjoc.19.118

Experimental procedures for all new compounds and summary of X-ray structure data for compound 8

License and Terms: This is a supporting information file under the terms of the Creative Commons Attribution License (https://creativecommons.org/licenses/by/4.0). Please note that the reuse, redistribution and reproduction in particular requires that the author(s) and source are credited and that individual graphics may be subject to special legal provisions.
The license is subject to the *Beilstein Journal of Organic Chemistry* terms and conditions: (https://www.beilstein-journals.org/bjoc/terms)

Table of contents

Experimental procedures..S2

Figure S1: ORTEP diagram of compound **8** X-ray structure S12

Table S1: Crystal data and structure refinement for compound **8** S13

References S13

General. Unless otherwise stated, all chemicals were obtained from commercial vendors and used without further purification. All reactions were carried out under an argon atmosphere unless otherwise noted. Anhydrous solvents were obtained by storing commercially available solvents over activated 4 Å molecular sieves. Photochemical reactions were conducted in a Luzchem 4V chamber containing 14 8-watt Hitachi FL8BL-B bulbs (λ_{max} 360 nm). Thin layer chromatography was performed using 0.25 mm E. Merck silica gel plates (60F-254) using UV light and either $KMnO_4$/heat or *p*-anisaldehyde/heat as visualizing agents. Flash silica gel chromatography was performed using a Biotage Isolera Prime with Sfär Duo cartridges or manually using 60 Å porosity silica gel (40–63 µm particle size). NMR spectra were recorded using a Bruker Avance III HD 400 spectrometer and calibrated using residual undeuterated solvent and TMS as references. The following abbreviations are used to describe peak multiplicities: s = singlet; d = doublet; t = triplet; q = quartet; m = multiplet; br = broad; app = apparent. HRMS was performed on a Waters Q-TOF Ultima spectrometer.
Tricarbonyl(tropone)iron, cationic complex **10**,[1] and 2-bromoallylamine (**5**)[2] were prepared according to literature procedures.

Synthesis of iron complexes

Synthesis of vinyl bromide **6**

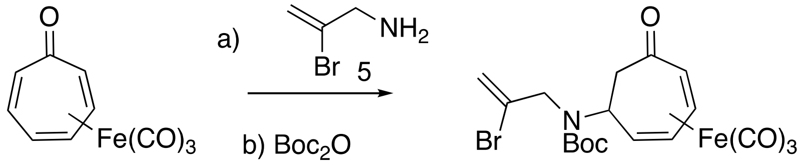

1 **6**

A 4-mL vial was charged with tricarbonyl(tropone)iron (100 mg, 0.4 mmol) and 2-bromoallylamine (272 mg, 2.0 mmol). The resulting red-brown viscous liquid was stirred for 16 h under ambient atmosphere. The progress of the reaction was monitored by removing a small aliquot and analyzing by ^{1}H NMR to confirm the disappearance of the starting iron complex. Upon reaction completion, the excess amine was removed in vacuo.
The crude red-brown oil was dissolved in ethanol (4 mL) and Boc_2O (436 mg, 2.0 mmol) was added followed by solid $NaHCO_3$ (269 mg, 3.2 mmol). The resulting mixture was sonicated for 1 h. Upon completion, the dark brown mixture was filtered through Celite and concentrated. The crude, oily product was then purified via flash chromatography (10→60% EtOAc in hexanes) to give the product **6** as a yellow solid (168 mg, 88%). R_f: 0.48 (1:1 hexanes: EtOAc); [NOTE: some NMR signals appear broadened and/or doubled due to the presence of slowly interconverting rotational isomers[3];] ^{1}H NMR (400 MHz, $CDCl_3$): δ 5.78 (app t, *J* = 5.6, 1 H), 5.72 (br s, 1 H), 5.59 (br s, 2 H), 4.66 (br s, 1 H), 4.11-4.06 (m, 1 H), 3.96-3.82 (m, 1 H), 3.24 (d, *J* = 6.6 Hz, 1 H), 3.14 (app d, *J* = 6.0 Hz, 1 H), 2.30 (m, 1 H), 2.18 (br s, 1 H), 1.49 (m, 9 H); ^{13}C NMR (100 MHz, $CDCl_3$): δ 208.0, 201.7, 153.8, 130.0, 117.4, 90.5, 90.3, 81.6, 81.1, 61.1, 59.7, 57.4, 52.7, 43.5, 42.7, 28.3. HRMS (ESI/Q-TOF) *m/z* $[M+H]^+$: Calcd for $C_{18}H_{21}BrFeNO_6$: 481.9902, found: 481.9905.

General procedure for additions of amines to cationic tropone iron complex 10

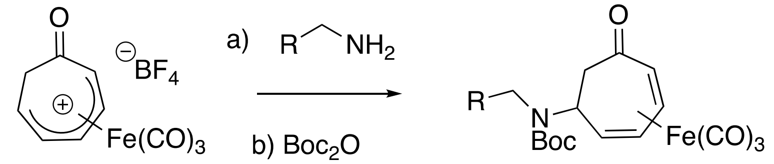

10

To a vigorously stirring solution of the amine (2.0 equiv) in ethyl acetate (≈0.2 M amine concentration) was added the cationic iron complex **10** (1.0 equiv). The resulting yellow suspension was allowed to stir for 1 h under ambient atmosphere. The reaction mixture was then diluted with ethyl acetate and washed with water. The aqueous layer was further extracted twice with ethyl acetate. The combined organic layers were dried over Na_2SO_4, filtered, and concentrated to give the crude addition product, typically a yellow oil or solid.
This crude material was dissolved in ethanol (≈0.1 M concentration) and Boc_2O (3.0 equiv) was added followed by solid $NaHCO_3$ (5.0 equiv). The resulting mixture (typically a yellow-orange suspension) was sonicated for 1 h under ambient atmosphere. Upon completion, the mixture was filtered through Celite and concentrated. The crude product was then purified via flash chromatography (silica gel, hexanes/EtOAc).

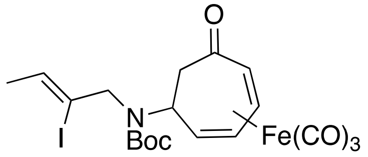

12

Compound 12: (*Z*)-2-iodo-2-buten-1-amine (**11**, 1.2 g, 6.0 mmol) and the cationic complex **10** (886 mg, 2.7 mmol) gave **12** as a yellow solid (994 mg, 68% over 2 steps) after flash chromatography (3:2 hexanes:EtOAc). R_f: 0.38 (3:2 hexanes: EtOAc); [NOTE: some NMR signals appear broadened and/or doubled due to the presence of slowly interconverting rotational isomers] ^{1}H NMR (400 MHz, $CDCl_3$): δ 5.82 (app q, *J* = 6.4 Hz, 1 H), 5.76 (t, *J* = 6.0 Hz, 1 H), 5.59 (br s, 1 H), 4.37 (br s, 1 H), 4.23 (br d, app *J* = 17.3 Hz, 1 H), 4.01-3.84 (br m, 1 H), 3.23 (app d, *J* = 6.7 Hz, 2 H), 2.49-2.29 (br m, 1 H), 2.14 (m, 1 H), 1.81 (d, *J* = 6.4 Hz, 3 H), 1.49 (br s, 9 H); ^{13}C NMR (100 MHz, $CDCl_3$): δ 208.1, 202.3, 154.3,153.8, 132.3, 106.0, 90.6, 90.2, 81.4, 80.9, 61.2, 60.7, 57.2 (2 C), 43.6, 42.8, 28.4, 21.7. HRMS (ESI/Q-TOF) *m/z* $[M+H]^+$: Calcd for $C_{19}H_{23}FeINO_6$: 543.5919, found: 543.9920.

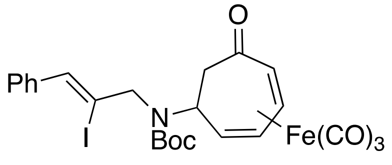

S1

Compound S1: (*Z*)-2-iodo-3-phenyl-2-propen-1-amine (104 mg, 0.4 mmol) and the cationic complex **10** (67 mg, 0.2 mmol) gave **S1** as a yellow oil (66 mg, 52% over 2 steps) after flash chromatography (3:2 hexanes:EtOAc). R_f: 0.28 (7:3 hexanes:EtOAc); [NOTE: some NMR signals appear broadened and/or doubled due to the presence of slowly interconverting rotational isomers] ^{1}H NMR (400 MHz, $CDCl_3$): δ 7.47 (app d, *J* = 7.7 Hz, 2 H), 7.39-7.32 (m, 3 H), 6.89

(br s, 1 H), 5.77 (t, J = 6.0 Hz, 1 H), 5.61 (br s, 1 H), 4.61 (br s, 1 H), 4.38 (m, 1 H), 4.21-3.99 (m, 1 H), 3.30-3.20 (m, 2 H), 2.44 (br s, 1 H), 2.28-2.18 (m, 1 H), 1.50 (br s, 9 H); ^{13}C NMR (100 MHz, $CDCl_3$): δ 208.0, 201.9, 153.9, 137.1, 135.1, 128.6, 128.2, 103.4, 90.4, 81.7, 81.2, 61.3, 60.3, 58.4, 57.4, 43.5, 42.9, 28.4. HRMS (ESI/Q-TOF) *m/z* $[M+H]^+$: Calcd for $C_{24}H_{25}FeINO_6$: 606.0076, found: 606.0070.

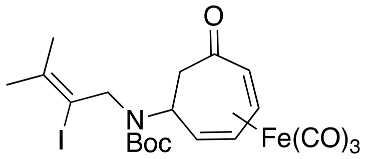

S2

Compound S2: 2-iodo-3-methyl-2-buten-1-amine (144 mg, 0.68 mmol) and the cationic complex **19** (76 mg, 0.23 mmol) gave **S2** as a yellow oil (65 mg, 51% over 2 steps) after flash chromatography (10→80% EtOAc in hexanes). R_f: 0.38 (3:2 hexanes: EtOAc); [NOTE: some NMR signals appear broadened and/or doubled due to the presence of slowly interconverting rotational isomers] ^{1}H NMR (400 MHz, $CDCl_3$): δ 5.74 (app t, J = 5.9 Hz, 1 H), 5.60 (br s, 1 H), 4.27-3.96 (m, 3 H), 3.40 (app d, J = 8.1 Hz, 1 H), 3.24 (d, J = 6.7 Hz, 1 H), 2.79-2.50 (m, 1H), 2.15 (app d, J = 8.6 Hz, 1 H), 2.04 (s, 3 H), 1.96 (s, 3 H), 1.52 (s, 9 H); ^{13}C NMR (100 MHz, $CDCl_3$): δ 208.3, 202.7, 141.0, 99.3, 91.0, 89.9, 80.7, 61.1, 57.5, 53.6, 48.7, 42.7, 32.0, 28.5/28.4, 20.3. HRMS (ESI/Q-TOF) *m/z* $[M+H]^+$: Calcd for $C_{20}H_{25}FeINO_6$: 558.0076, found: 558.0078.

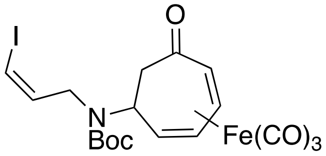

S3

Compound S3: (*Z*)-3-iodo-2-propen-1-amine (113 mg, 0.62 mmol) and the cationic complex **10** (103 mg, 0.31 mmol) gave **S3** as a yellow solid (69 mg, 42% over 2 steps) after flash chromatography (3:2 hexanes: EtOAc). R_f: 0.36 (3:2 hexanes: EtOAc); [NOTE: some NMR signals appear broadened and/or doubled due to the presence of slowly interconverting rotational isomers] ^{1}H NMR (400 MHz, $CDCl_3$): δ 6.36-6.25 (m, 2 H), 5.82 (m, 1 H), 5.62 (t, J = 7.2 Hz, 1 H), 4.91 (m, 1 H), 3.90-3.83 (m, 2 H), 3.24 (d, J = 6.7 Hz, 1 H), 3.02 (d, J = 7.8 Hz, 1 H), 2.19 (app t, J = 12.0 Hz, 1 H), 2.11 (m, 1 H), 1.49 (s, 9 H); ^{13}C NMR (100 MHz, $CDCl_3$): δ 207.9, 201.5, 154.1, 138.8, 90.6, 90.2, 82.6, 80.9, 80.7, 60.3, 60.1, 57.3, 48.3, 43.8,43.1, 28.4. HRMS (ESI/Q-TOF) *m/z* $[M+H]^+$: Calcd for $C_{18}H_{21}FeINO_6$: 529.9763, found: 529.9760

General procedure for synthesis of (*Z*)-2-iodo-2-buten-1-amine, (*Z*)-2-iodo-3-phenyl-2-propen-1-amine, and **2-iodo-3-methyl-2-buten-1-amine** (the route to (*Z*)-2-iodo-2-buten-1-amine shown below is representative)

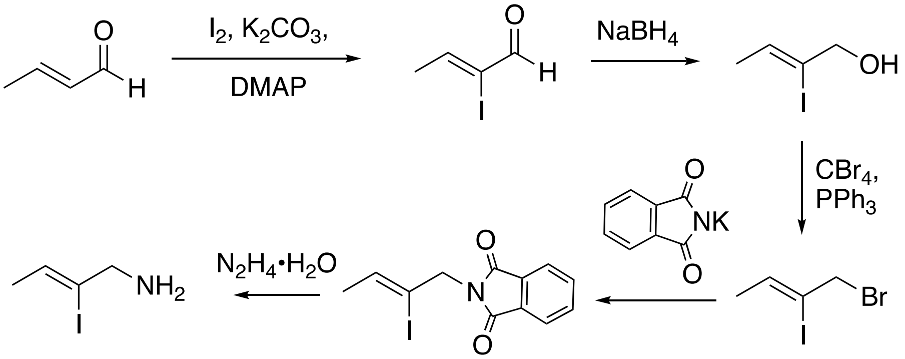

To solution of the appropriate aldehyde (1.0 equiv) in THF/H_2O (1:1, 0.2 M aldehyde concentration) was added K_2CO_3 (1.2 equiv), I_2 (2.0 equiv), and DMAP (0.2 equiv). The reaction mixture was allowed to stir overnight. The mixture was then diluted with CH_2Cl_2 and washed sequentially with saturated aqueous $Na_2S_2O_3$ and brine. The organic layer was dried over Na_2SO_4, filtered, and concentrated to give crude product as a brown liquid, which was carried forward without further purification.

The crude iodoaldehyde was dissolved in THF/H_2O (9:1, ≈0.4 M aldehyde concentration) and cooled to 0 ºC. $NaBH_4$ (1.1 equiv) was added in portions, after which the dark brown color became much lighter. After stirring for 1 h, the reaction mixture was poured into water and extracted with EtOAc (3×). The combined organic layers were washed with brine and dried over Na_2SO_4, filtered, and concentrated. The crude material was carried to the next step without further purification.

The crude alcohol from the reduction step was dissolved in acetonitrile (≈0.2 M alcohol concentration) and CBr_4 (2.0 equiv) was added. The solution was cooled to 0 ºC and PPh_3 (2.0 equiv) was added slowly. The reaction mixture was allowed to stir overnight, over which time an off-white precipitate formed. The acetonitrile solvent was then removed *in vacuo* and the resulting residue was suspended in a 4:1 mixture of hexanes/EtOAc (about ¼ the volume of acetonitrile used) and sonicated for 5 min. The supernatant was then passed through a pad of silica gel, eluting with additional 4:1 hexanes/EtOAc. The filtrate was concentrated to give the crude bromide product that was carried forward without additional purification.

The crude allylic bromide was dissolved in DMF (≈0.4 M concentration) and potassium phthalimide (1.2 equiv) was added. The resulting suspension was stirred overnight. The reaction mixture was then diluted with Et_2O and washed three times with water and once with brine. The organic layer was dried over Na_2SO_4, filtered, and concentrated to give the crude phthalimide, which was carried forward without purification.

The phthalimide was dissolved in ethanol (0.3 M concentration) and hydrazine hydrate (50% hydrazine by weight; 3.0 equiv) was added. The initially heterogeneous mixture was heated to reflux and stirred for 1 h. The mixture initially becomes clear, with a white precipitate forming as the reaction proceeds. After 1 h, 2.0 M HCl (≈3 mL per mmol substrate) was added and heating continued for an additional hour. The reaction vessel was removed from the heating bath and briefly cooled in ice, after which the white precipitate was filtered off. The filtrate was concentrated in vacuo and the resulting solid residue was dissolved in 2 M NaOH (≈5 mL per mmol substrate). The resulting solution was extracted with diethyl ether (5×). The combined ether extracts were dried over Na_2SO_4, filtered and concentrated. The resulting crude amine was deemed to be of sufficient purity for use in subsequent addition reactions.

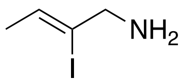

(*Z*)-2-Iodo-2-buten-1-amine: Crotonaldehyde (71.4 mmol) gave the title compound as a yellow liquid (2.4 g, 17% over five steps) whose ^{1}H and ^{13}C NMR spectra were consistent with the literature.[4] ^{1}H NMR (400 MHz, $CDCl_3$): δ 5.82 (q, *J* = 6.4 Hz, 1 H), 3.49 (s, 2 H), 1.77 (d, *J* = 6.4 Hz, 3 H), 1.50 (s, 2 H); ^{13}C NMR (100 MHz, $CDCl_3$): δ 130.0, 114.2, 54.7, 21.7

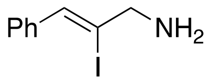

(*Z*)-2-Iodo-3-phenyl-2-propen-1-amine: Cinnamaldehyde (8.0 mmol) gave the title compound as a yellow liquid (0.56 g, 27% over five steps) whose ^{1}H and ^{13}C NMR spectra were consistent with the literature.[5] ^{1}H NMR (400 MHz, $CDCl_3$): δ 7.48 (d, *J* = 8.0 Hz, 2 H), 7.38-7.28 (m, 4 H), 6.91 (s, 1 H), 3.64 (s, 2 H), 1.65 (br s, 2 H) ; ^{13}C NMR (100 MHz, $CDCl_3$): δ 137.5, 133.0, 128.6, 128.1, 128.0, 112.5, 56.6

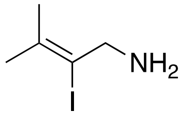

2-Iodo-3-methyl-2-buten-1-amine: 3-methyl-2-butenal (30 mmol) gave the title compound (0.57 g, 9 % over five steps) as a yellow liquid. R_f: 0.16 (95:5 CH_2Cl_2:MeOH); ^{1}H NMR (400 MHz, $CDCl_3$): δ 3.53 (s, 2 H), 1.95 (s, 3 H), 1.89 (s, 3 H), 1.52 (br, 2 H); ^{13}C NMR (100 MHz, $CDCl_3$): δ 137.1, 107.3, 50.7, 31.7, 19.6; HRMS (ESI/Q-TOF) *m/z* $[M+H]^+$: Calcd for $C_5H_{11}IN$: 211.9936, found: 211.9937.

Synthesis of (*Z*)-3-iodo-2-propen-1-amine

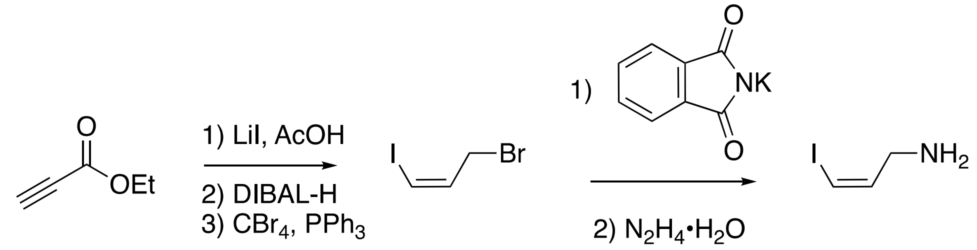

To a solution of ethyl propiolate (0.97 mL, 10.2 mmol) in acetonitrile (10 mL) was added LiI (1.5 g, 11.2 mmol) and glacial acetic acid (0.64 mL, 11.2 mmol). The resulting yellow solution was heated to reflux and stirred for 16 h. The resulting yellow suspension was then cooled to room temperature and aqueous K_2CO_3 (0.3 M, 20 mL) was added. The mixture was extracted with Et_2O (4 × 10 mL). The organic layers were washed with brine (40 mL) and dried over Na_2SO_4, filtered, and concentrated to give an orange-yellow oil which was carried forward without further purification.

The crude material was dissolved in anhydrous Et_2O (85 mL) and cooled to 0 °C under an argon atmosphere. DIBAL-H (1.0 M in toluene, 36 mL, 36 mmol) was then carefully added via syringe, during which the initially deep yellow solution becomes much lighter in color. After addition was complete, the reaction mixture was stirred for 30 min. Then, 2 mL of MeOH were added, followed by 75 mL of saturated aqueous sodium potassium tartrate. The cloudy suspension was then stirred for 16 h, after which time it became clear and formed two layers

upon cessation of stirring. The two layers were separated and the aqueous layer was further extracted with Et_2O (2 × 40 mL). The combined organic layers were dried over Na_2SO_4 to give a pale orange liquid which was carried forward without further purification.

The crude material was dissolved in acetonitrile (85 mL) and CBr_4 (11.3 g, 34 mmol) was added. The solution was cooled to 0 °C and PPh_3 (8.9 g, 34 mmol) was added slowly. The reaction mixture was allowed to stir for 16 h, over which time an off-white precipitate formed. The acetonitrile solvent was then removed in vacuo and the resulting residue was suspended in a 4:1 mixture of hexanes/EtOAc (20 mL) and sonicated for 5 min. The supernatant was then passed through a pad of silica gel, eluting with additional 4:1 hexanes/EtOAc. The filtrate was concentrated to give the crude bromide product as a salmon-colored liquid, which was carried forward without additional purification.

The crude allylic bromide was dissolved in DMF (12 mL) and potassium phthalimide (1.18 g, 6.4 mmol) was added. The resulting suspension was stirred for 16 h. The reaction mixture was then diluted with Et_2O (30 mL) and washed three times with water (20 mL) and once with brine (20 mL). The organic layer was dried over Na_2SO_4, filtered, and concentrated to give the crude phthalimide, which was purified by flash chromatography (8:2 hexanes:EtOAc) to give a crystalline white solid (0.64 g, 12% over four steps).

The phthalimide was dissolved in ethanol (7 mL) and hydrazine hydrate (50% hydrazine by weight; 0.26 mL, 4.1 mmol) was added. The initially heterogeneous mixture was heated to reflux and stirred for 1 h. The mixture initially becomes clear, with a white precipitate forming as the reaction proceeds. After 1 h, 2.0 M HCl (6 mL) was added and heating continued for an additional hour. The reaction vessel was removed from the heating bath and briefly cooled in ice, after which the white precipitate was filtered off. The filtrate was concentrated in vacuo and the resulting solid residue was dissolved in 2 M NaOH (10 mL). The resulting solution was extracted with diethyl ether (5 × 10 mL). The combined ether extracts were dried over Na_2SO_4, filtered and concentrated to give a yellow liquid (0.28 g, 76%) which was sufficiently pure for subsequent addition reactions. R_f: 0.11 (95:5 CH_2Cl_2:MeOH); ^{1}H NMR (400 MHz, $CDCl_3$): δ 6.34 (q, J = 7.2 Hz, 1 H), 6.26 (d, J = 7.8 Hz, 1 H), 3.39 (d, J = 6.0 Hz, 2 H), 1.47 (br s, 2 H); ^{13}C NMR (100 MHz, $CDCl_3$): δ 141.8, 82.1, 46.3; HRMS (ESI/Q-TOF) m/z $[M+H]^+$: Calcd for C_3H_7IN: 183.9623, found: 183.9625

General procedure for photodemetallation of iron complexes

In a microwave vial, the iron complex was dissolved in glacial acetic acid (0.02 M). The vial was sealed and argon was bubbled through the solution for 20 min. The vial was then placed in the UV chamber (see General) and irradiated for 4 h. The reaction mixture was then carefully poured into saturated aqueous Na_2CO_3 and extracted with EtOAc (3×). The combined organic layers were then washed with saturated aqueous $NaHCO_3$ and brine. The organic layers were then dried over Na_2SO_4, filtered and concentrated. The crude material (typically a colorless or pale brown oil) was purified via flash chromatography.

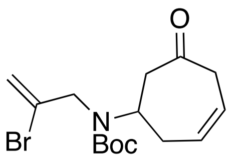

7

Compound 7: Compound **6** (48 mg, 0.1 mmol) gave the title compound (25 mg, 74%) as a clear colorless oil that also contained a small amount of the conjugated enone isomer (≈6%). R_f: 0.24 (7:3 hexanes:EtOAc); [NOTE: some NMR signals appear broadened and/or doubled due to the presence of slowly interconverting rotational isomers; ~2:1 ratio of rotamers] ^{1}H NMR (400 MHz, $CDCl_3$): δ 5.81-5.75 (m, 1 H), 5.73 (s, 1 H), 5.63-5.59 (m, 1 H), 5.58 (s, 1 H), 4.65 (br s, 0.65 H), 4.26 (br s, 0.30 H), 4.09-3.93 (m, 2 H), 3.42-3.25 (m, 1 H), 3.05 (dd, *J* = 15.8, 7.4 Hz, 1 H), 3.00-2.88 (m, 1 H), 2.77 (dd, *J* = 15.2, 4.7 Hz, 1 H), 2.72-2.57 (m, 1 H), 2.50 (m, 1 H), 1.46 (s, 9 H); ^{13}C NMR (100 MHz, $CDCl_3$): δ 207.0, 153.9, 128.3, 122.3, 116.4, 80.6, 52.6, 52.0, 48.3, 42.9, 33.2, 28.3; HRMS (ESI/Q-TOF) *m/z* $[M+Na]^+$: Calcd for $C_{15}H_{22}BrNO_3Na$: 366.0681, found: 366.0681.

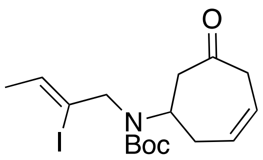

9

Compound 9: Compound **12** (119 mg, 0.2 mmol) gave the title compound (63 mg, 74%) as a clear colorless oil. R_f: 0.23 (8:2 hexanes:EtOAc); [NOTE: some NMR signals appear broadened and/or doubled due to the presence of slowly interconverting rotational isomers] ^{1}H NMR (400 MHz, $CDCl_3$): δ 5.84-5.75 (m, 2 H), 5.61-5.55 (m, 1 H), 4.50-3.93 (m, 3 H), 3.38-3.24 (br s, 1 H), 3.05 (dd, *J* = 15.5, 7.2 Hz, 2 H), 2.76 (app d, *J* = 15.2 Hz, 2 H), 2.49 (app d, *J* = 16.4 Hz, 1 H), 1.80 (d, *J* = 6.5 Hz, 3 H), 1.47 (s, 9 H); ^{13}C NMR (100 MHz, $CDCl_3$): δ 206.5, 154.5, 130.5, 129.6, 121.5, 106.8, 80.6, 57.4, 56.4, 53.2, 52.2, 49.7, 48.7, 42.8, 33.7, 28. 7, 21.7; HRMS (ESI/Q-TOF) *m/z* $[M+Na]^+$: Calcd. for $C_{16}H_{24}INO_3Na$: 428.0699, found: 428.0705.

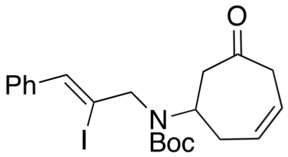

13

Compound 13: Compound **S1** (66 mg, 0.11 mmol) gave the title compound (28 mg, 55%) as a clear yellow oil. R_f: 0.33 (7:3 hexanes:EtOAc); [NOTE: some NMR signals appear broadened and/or doubled due to the presence of slowly interconverting rotational isomers] ^{1}H NMR (400 MHz, $CDCl_3$): δ 7.49 (d, *J* = 7.6 Hz, 2 H), 7.41-7.32 (m, 3 H), 6.91 (br s, 1 H), 5.86-5.80 (m, 1 H), 5.66-5.60 (m, 1 H), 4.65-4.22 (m, 3 H), 3.35 (br s, 1 H), 3.10-3.06 (m, 2 H), 2.88-2.76 (m, 2 H), 2.58 (app d, *J* = 16.6 Hz, 1 H), 1.51 (s, 9 H); ^{13}C NMR (100 MHz, $CDCl_3$): δ 206.3, 154.7, 137.3, 133.6, 129.3, 128.6, 128.2, 121.8, 104.0, 81.0, 57.8, 52.6, 48.6, 43.0, 34.1, 28.4; HRMS (ESI/Q-TOF) *m/z* $[M+Na]^+$: Calcd. for $C_{21}H_{26}INO_3Na$: 490.0855, found: 490.0862

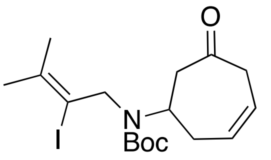

15

Compound 15: Compound **S2** (50 mg, 0.09 mmol) gave the title compound (11 mg, 29%) as a clear colorless oil. R_f: 0.46 (7:3 hexanes:EtOAc); ^{1}H NMR (400 MHz, $CDCl_3$): δ 5.83-5.77 (m, 1 H), 5.60-5.53 (m, 1 H), 4.28-3.93 (br m, 3 H), 3.32-3.16 (m, 2 H), 3.11-3.03 (m, 1 H), 2.93-2.79 (m, 2 H), 2.54 (br d, J = 16.0 Hz, 1 H), 2.00 (s, 3 H), 1.93 (s, 3 H), 1.49 (s, 9H); ^{13}C NMR (100 MHz, $CDCl_3$): δ 206.6, 154.8, 139.7, 130.1, 121.2, 100.0, 80.7, 53.2, 49.4, 42.8, 34.3, 32.1, 28.5, 19.7; HRMS (ESI/Q-TOF) *m/z* $[M+Na]^+$: Calcd. for $C_{17}H_{26}INO_3Na$: 442.0855, found: 442.0867.

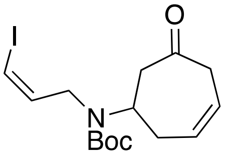

17

Compound 17: Compound **S3** (69 mg, 0.13 mmol) gave the title compound (20 mg, 39%) as a clear colorless oil. R_f: 0.41 (7:3 hexanes:EtOAc); [NOTE: some NMR signals appear broadened and/or doubled due to the presence of slowly interconverting rotational isomers; ~1.6:1 ratio of rotamers] ^{1}H NMR (400 MHz, $CDCl_3$): δ 6.31 (m, 1 H), 6.27 (br s, 1 H), 5.78 (br s, 1 H), 5.59 (br s, 1 H), 4.80 (br s, 0.64 H), 4.34 (br s, 0.39 H), 3.91-3.75 (m, 2 H), 3.40 (br s, 1 H), 3.04-3.02 (m, 1 H), 2.89 (br s, 1 H), 2.71-2.60 (m, 2 H), 2.43 (app d, J = 17.4 Hz, 1 H), 1.47 (s, 9 H); ^{13}C NMR (100 MHz, $CDCl_3$): δ 206.2, 154.7, 139.2, 129.1, 121.4, 81.8, 80.7, 50.8, 48.5, 48.4, 42.8, 34.6, 28.4; HRMS (ESI/Q-TOF) *m/z* $[M+Na]^+$: Calcd. for $C_{15}H_{22}INO_3Na$: 414.0542, found: 414.0547.

General procedure for intramolecular Heck reactions

In a microwave vial, the vinyl halide starting material (0.1 mmol) was dissolved in dry toluene (7 mL) and K_3PO_4 (3 equiv), phenol (0.2 equiv), $Pd(PPh_3)_4$ (0.2 equiv), and triethylamine (6 equiv) were added. The microwave vial was sealed and the bright yellow-orange mixture was degassed by bubbling argon through the mixture for 25 min. The mixture was then heated to 110 °C. When the reaction was judged complete by TLC, the resulting brown mixture was diluted with Et_2O (25 mL) and washed with saturated aqueous Na_2CO_3 (30 mL) and brine (30 mL). The organic layer was dried over Na_2SO_4, filtered through Celite, and concentrated in vacuo. The crude product was purified via flash chromatography.

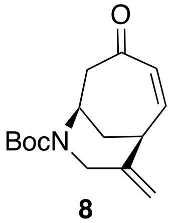

8

Compound 8: Following the general procedure, vinyl bromide **7** (34 mg, 0.1 mmol) gave compound **8** (11 mg, 42%) as a yellow oil. X-ray quality crystals were grown via vapor diffusion of hexane with a saturated solution of **8** in methylene chloride. R_f: 0.26 (7:3 hexanes:EtOAc);

[NOTE: some NMR signals appear broadened and/or doubled due to the presence of slowly interconverting rotational isomers; ~1:1 ratio of rotamers] ^{1}H NMR (400 MHz, $CDCl_3$): 6.42 (dd, *J* = 12.4, 8.0 Hz, 1 H), 6.08 (d, *J* = 12.4 Hz, 1 H), 5.01-4.94 (m, 2 H), 4.65 (br s, 0.5 H) 4.48-4.41 (m, 1 H), 4.25 (app d, *J* = 15.7 Hz, 0.5 H), 3.40-3.37 (m, 2 H), 2.96 (app t, *J* = 18.6 Hz, 1 H), 2.76 (dd, *J* = 17.0, 5.1 Hz, 1 H), 2.21-2.18 (m, 2 H), 1.47 (s, 9 H); ^{13}C NMR (100 MHz, $CDCl_3$): δ 202.5, 201.9, 154.3, 144.2, 144.0, 140.1, 140.0, 132.7, 132.6, 113.3, 112.8, 80.3, 49.9, 49.3, 45.6, 44.6, 42.9, 42.9, 41.7, 33.9, 33.8, 28.3; HRMS (ESI/Q-TOF) *m/z* [M+Na]$^+$: Calcd. for $C_{15}H_{21}NO_3Na$: 286.1419, found: 286.1415.

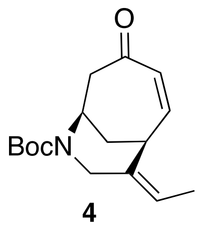

4

Compound 4: Following the general procedure, vinyl iodide **9** (41 mg, 0.1 mmol) gave compound **4** (21 mg, 76%) as a clear colorless oil. R_f: 0.29 (7:3 hexanes:EtOAc); [NOTE: some NMR signals appear broadened and/or doubled due to the presence of slowly interconverting rotational isomers; ~1:1 ratio of rotamers] ^{1}H NMR (400 MHz, $CDCl_3$): δ 6.37 (dd, *J* = 12.4, 8.0 Hz, 1 H), 6.09 (d, *J* = 12.2 Hz, 1 H), 5.60-5.45 (m, 1 H), 4.64 (br s, 0.5 H), 4.48 (br s, 0.5 H), 4.31 (app d, *J* = 15.2 Hz, 0.5 H), 4.13 (m, 0.5 H), 3.78 (br s, 1 H), 3.53-3.51 (m, 1 H), 2.96 (app t, *J* = 18.0 Hz, 1 H), 2.76 (dd, *J* = 17.2, 5.7 Hz, 1 H), 2.23-2.07 (m, 2 H), 1.70 (d, *J* = 6.7 Hz, 3 H), 1.46 (s, 9 H); ^{13}C NMR (100 MHz, $CDCl_3$): δ 202.6, 202.1, 154.3, 143.3, 132.6, 130.8, 130.3, 122.5, 122.1, 80.1, 49.9, 49.4, 46.1, 45.1, 44.1, 42.8, 36.1, 33.4, 28.4, 12.7; HRMS (ESI/Q-TOF) *m/z* [M+Na]$^+$: Calcd. for $C_{16}H_{23}NO_3Na$: 300.1576, found: 300.1569.

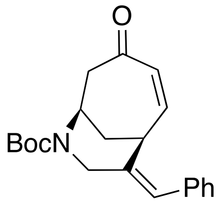

14

Compound 14: Following the general procedure, vinyl iodide **13** (28 mg, 0.06 mmol) gave compound **14** (10 mg, 50%) as a pale brown oil. R_f: 0.29 (7:3 hexanes:EtOAc). [NOTE: some NMR signals appear broadened and/or doubled due to the presence of slowly interconverting rotational isomers; ~1:1 ratio of rotamers] ^{1}H NMR (400 MHz, $CDCl_3$): δ 7.37 (m, 2 H), 7.30-7.23 (m, 3 H), 6.64 (br s, 0.6 H), 6.57 (br s, 0.5 H), 6.51-6.45 (m, 1 H), 6.18 (d, *J* = 12.3 Hz, 1 H), 4.69 (br s, 0.5 H), 4.54-4.49 (m, 1 H), 4.32 (app d, *J* = 15.0 Hz, 0.5 H), 3.88 (br m, 1 H), 3.71-3.58 (m, 1 H), 3.00 (m, 1 H), 2.76 (dd, *J* = 17.0, 5.0 Hz, 1 H), 2.13 (br s, 2 H), 1.49 (br s, 9 H); ^{13}C NMR (100 MHz, $CDCl_3$): δ 202.5, 202.0, 154.3, 143.5, 143.3, 136.3, 133.3, 128.6, 128.3, 127.8, 127.4, 115.3, 80.4, 50.0, 49.4, 46.1, 45.1, 44.5, 43.2, 37.0, 33.8, 26.5; HRMS (ESI/Q-TOF) *m/z* [M+Na]$^+$: Calcd. for $C_{21}H_{25}NO_3Na$: 362.1732, found: 362.1733.

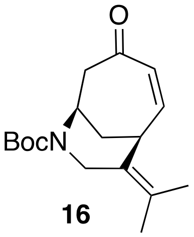

16

Compound 16: Following general procedure A, vinyl iodide **15** (11 mg, 0.026 mmol) gave compound **16** (6 mg, 75 %) as a clear colorless oil. R_f 0.31 (7:3 hexanes:EtOAc). [NOTE: some NMR signals appear broadened and/or doubled due to the presence of slowly interconverting rotational isomers] ^{1}H NMR (400 MHz, $CDCl_3$): δ 6.35 (dd, *J* = 12.4, 8.4 Hz, 1 H), 6.04 (d, *J* = 12.5 Hz, 1 H), 4.89-4.74 (m, 1 H), 4.62 (br s, 0.5 H) 4.44 (br s, 0.5 H), 3.85-3.79 (m, 1 H), 3.18 (br s, 1 H), 2.96 (app d, *J* = 16.6 Hz, 1 H), 2.76 (app d, *J* = 16.5 Hz, 1 H), 2.18-2.10 (m, 2 H), 1.78 (s, 3 H), 1.76 (s, 3 H), 1.47 (s, 9 H); ^{13}C NMR (100 MHz, $CDCl_3$): δ 202.8, 202.3, 154.3, 143.9, 132.0, 128.7, 128.3, 123.2, 122.6, 80.0, 49.9, 49.5, 45.6, 44.7, 37.7, 37.1, 33.4, 28.5, 20.0; HRMS (ESI/Q-TOF) *m/z* $[M+Na]^+$: Calcd. for $C_{17}H_{25}NO_3Na$: 314.1732, found: 314.1735

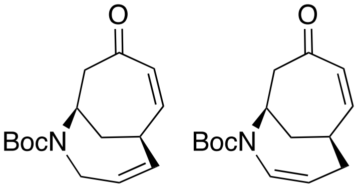

18 **19**

Compounds 18/19: Following general procedure A, vinyl iodide **S3** (60 mg, 0.15 mmol) gave an inseparable mixture of **18** and **19** (6 mg, 15% overall yield) as a clear, colorless oil. R_f 0.55 (4:6 hexanes:EtOAc). [NOTE: some NMR signals appear broadened and/or doubled due to the presence of slowly interconverting rotational isomers] **18**: ^{1}H NMR (400 MHz, $CDCl_3$): δ 6.71 (app t, *J* = 9.9 Hz, 1 H), 6.02 (d, *J* = 11.6 Hz, 1 H), 5.72 (m, 2 H), 4.54 (br s, 1 H), 4.28 (d, *J* = 18.7 Hz, 1 H), 4.07 (br s, 1 H), 3.57 (br s, 1 H), 3.15 (t, *J* = 11.9 Hz, 1 H), 2.95 (dd, *J* = 12.3, 7.1 Hz, 1 H), 2.47 (app d, *J* = 12.0 Hz, 1 H), 2.15 (app dd, *J* = 16.4, 7.0 Hz, 1 H), 1.45 (s, 9 H); ^{13}C NMR (100 MHz, $CDCl_3$): δ 200.7, 154.8, 145.2, 132.1, 129.0 (2C), 80.3, 50.2, 48.8, 41.2, 40.6, 34.7, 28.5. **19**: ^{1}H NMR (400 MHz, $CDCl_3$): δ 6.71 (t, *J* = 9.9 Hz, 1 H), 6.34 (d, *J* = 8.2 Hz, 1 H), 6.02 (d, *J* = 11.6 Hz, 1 H), 5.53 (q, *J* = 7.4 Hz, 1 H), 4.66 (br s, 1 H), 3.89 (br s, 2 H), 3.57 (br s, 1 H), 3.15 (t, *J* = 11.9 Hz, 1 H), 2.95 (dd, *J* = 12.3, 7.1 Hz, 1 H), 2.47 (br d, *J* = 12.0 Hz, 1 H), 2.15 (dd, *J* = 16.4, 7.0 Hz, 1 H), 1.48 (s, 9 H); ^{13}C NMR (100 MHz, $CDCl_3$): δ 200.7, 155.8, 145.2, 132.1, 129.3, 124.9, 80.3, 50.2, 48.8, 41.2, 37.6, 34.7, 28.3. HRMS (ESI/Q-TOF) *m/z* $[M+Na]^+$: Calcd. for $C_{15}H_{21}NO_3Na$: 286.1419, found: 286.1412.

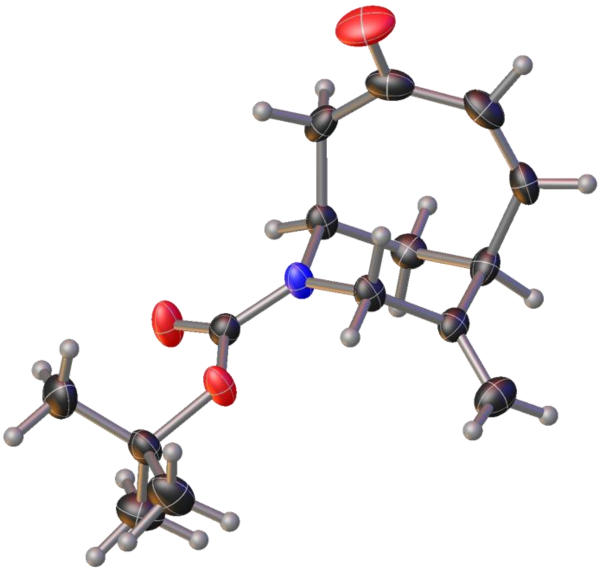

Figure S1. ORTEP diagram of compound **8**

Table S1. Crystal data and structure refinement for compound **8.**

```
Bond precision:   C-C = 0.0055 A          Wavelength=0.71073

Cell:              a=6.144(2)     b=6.122(2)          c=19.583(4)
                   alpha=90       beta=98.95(3)       gamma=90
Temperature:       173 K

                 Calculated                Reported
Volume           727.6(4)                  727.7(4)
Space group      P 21                      P 1 21 1
Hall group       P 2yb                     P 2yb
Moiety formula   C15 H21 N O3              C15 H21 N O3
Sum formula      C15 H21 N O3              C15 H21 N O3
Mr               263.33                    263.33
Dx,g cm-3        1.202                     1.202
Z                2                         2
Mu (mm-1)        0.083                     0.083
F000             284.0                     284.0
F000'            284.13
h,k,lmax         7,7,24                    7,7,24
Nref             2987[ 1639]               2723
Tmin,Tmax        0.991,0.993               0.667,0.745
Tmin'            0.973

Correction method= # Reported T Limits: Tmin=0.667 Tmax=0.745
AbsCorr = NONE

Data completeness= 1.66/0.91      Theta(max)= 26.416

R(reflections)= 0.0355( 2647)     wR2(reflections)= 0.0826( 2723)

S = 1.102                  Npar= 176
```

[1] Huang, Z.; Phelan, Z. K.; Tritt, R. L.; Valent, S. D.; Guan, Z.; He, Y.; Weiss, P. S.; Griffith, D. R. *J. Visualized Exp.* **2019**, *2019,* No. e60050

[2] Fort, D. A.; Woltering, T. J.; Nettekoven, M.; Knust, H.; Bach, T. *Chem. Commun.* **2013**, *49*, 2989-2991.

[3] Similar features were observed in the NMR spectra of other rigid, Boc-protected azapolycycles and their precursors from our previous work. We found that removal and/or replacement of the Boc group on such compounds with a more conformationally mobile protecting group resulted in sharper signals that were not doubled. See Phelan, Z. K.; Weiss, P. S.; He, Y.; Guan, Z.; Thamattoor, D. M.; Griffith, D. R. *J. Org. Chem.* **2020**, *85*, 2202-2212.

[4] Liu, P.; Wang, J.; Zhang, J.; Qiu, F. G.; *Org. Lett.* **2011**, *13*, 6426-6428.

[5] Bowman, W. R.; Cloonan, M. O.; Fletcher, A. J.; Stein, T. *Org. Biomol. Chem.* **2005**, *3*, 1460-1467.
