# Supplementary material for: Synthesis of 7-azabicyclo[4.3.1]decane ring systems from tricarbonyl(tropone)iron via intramolecular Heck reactions
Source: Beilstein J Org Chem. 2023 Oct 23;19:1615–9. doi: 10.3762/bjoc.19.118 (PMC10616702; doi:10.3762/bjoc.19.118)
Supplement: File 2 — Copies of 1H and 13C NMR spectra of all purified novel compounds. [file Beilstein_J_Org_Chem-19-1615-s002.pdf]

BEILSTEIN JOURNAL OF ORGANIC CHEMISTRY

Supporting Information

for

Synthesis of 7-azabicyclo[4.3.1]decane ring systems from tricarbonyl(tropone)iron via intramolecular Heck reactions

Aaron H. Shoemaker, Elizabeth A. Foker, Elena P. Uttaro, Sarah K. Beitel and Daniel R. Griffith

Beilstein J. Org. Chem. **2023,** *19,* 1615–1619. doi:10.3762/bjoc.19.118

Copies of ^{1}H and ^{13}C NMR spectra of all purified novel compounds

License and Terms: This is a supporting information file under the terms of the Creative Commons Attribution License (https://creativecommons.org/licenses/by/4.0). Please note that the reuse, redistribution and reproduction in particular requires that the author(s) and source are credited and that individual graphics may be subject to special legal provisions.
The license is subject to the *Beilstein Journal of Organic Chemistry* terms and conditions: (https://www.beilstein-journals.org/bjoc/terms)

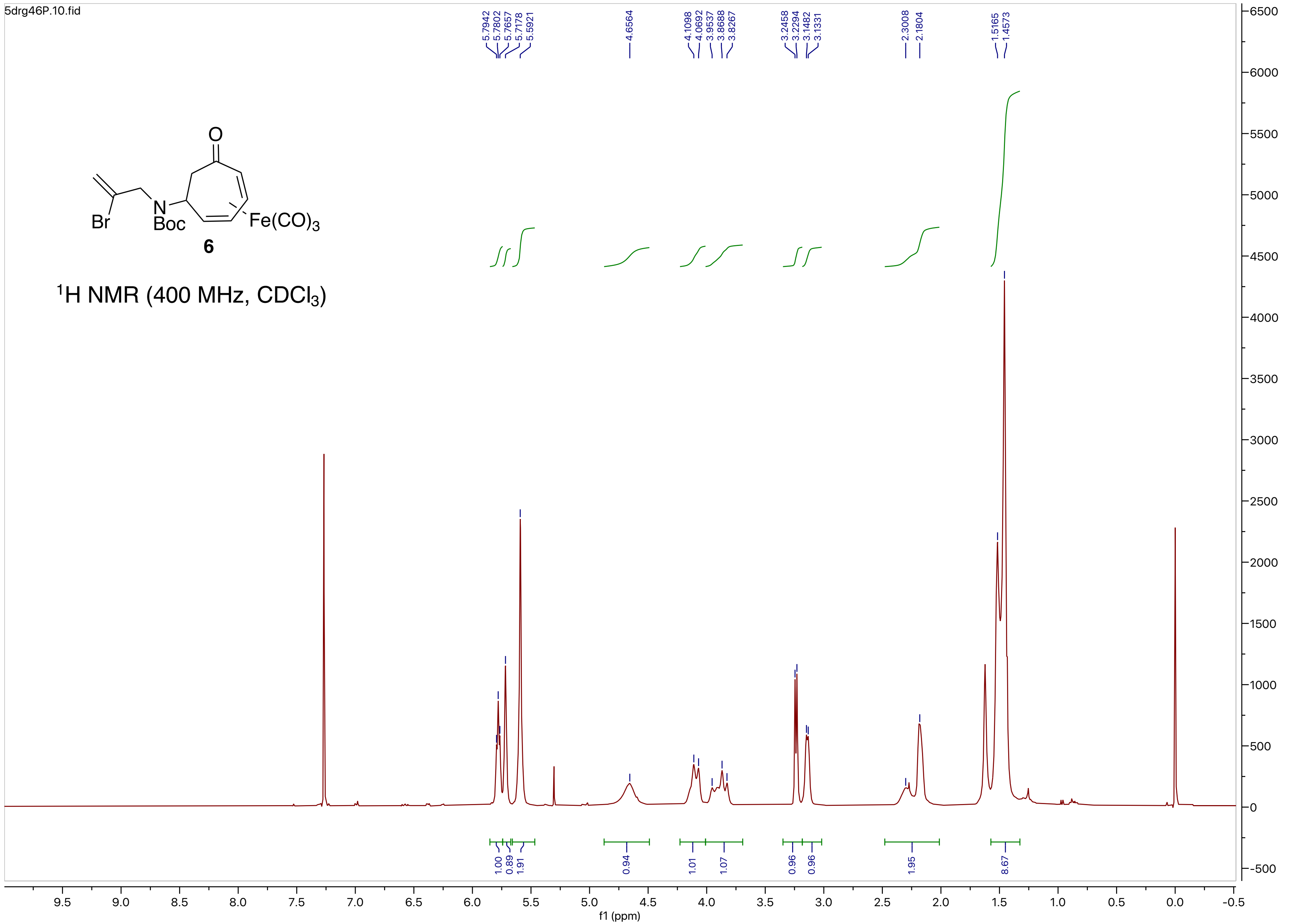
5drg46P.10.fid
O
Br
N
Boc
Fe(CO)3
6
1H NMR (400 MHz, CDCl3)
5.7942
5.7802
5.7657
5.7178
5.5921
4.6564
4.1098
4.0692
3.9537
3.8688
3.8267
3.2458
3.2294
3.1482
3.1331
2.3008
2.1804
1.5165
1.4573
1.00
0.89
1.91
0.94
1.01
1.07
0.96
0.96
1.95
8.67
f1 (ppm)

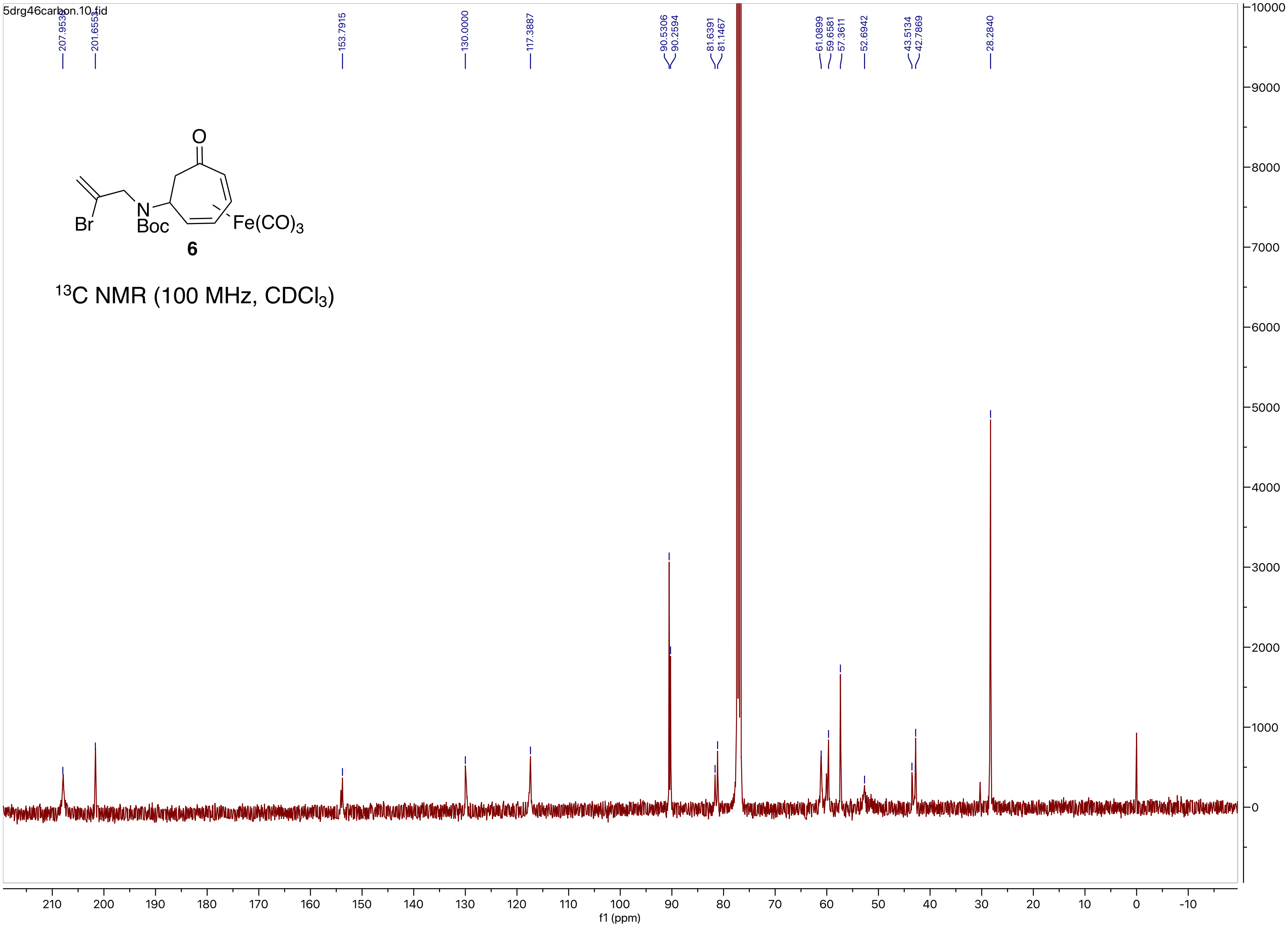
5drg46carbon.10.fid
207.9530
201.6553
153.7915
130.0000
117.3887
90.5306
90.2594
81.6391
81.1467
61.0899
59.6581
57.3611
52.6942
43.5134
42.7869
28.2840
O
N
Br
Boc
Fe(CO)3
6
13C NMR (100 MHz, CDCl3)
f1 (ppm)

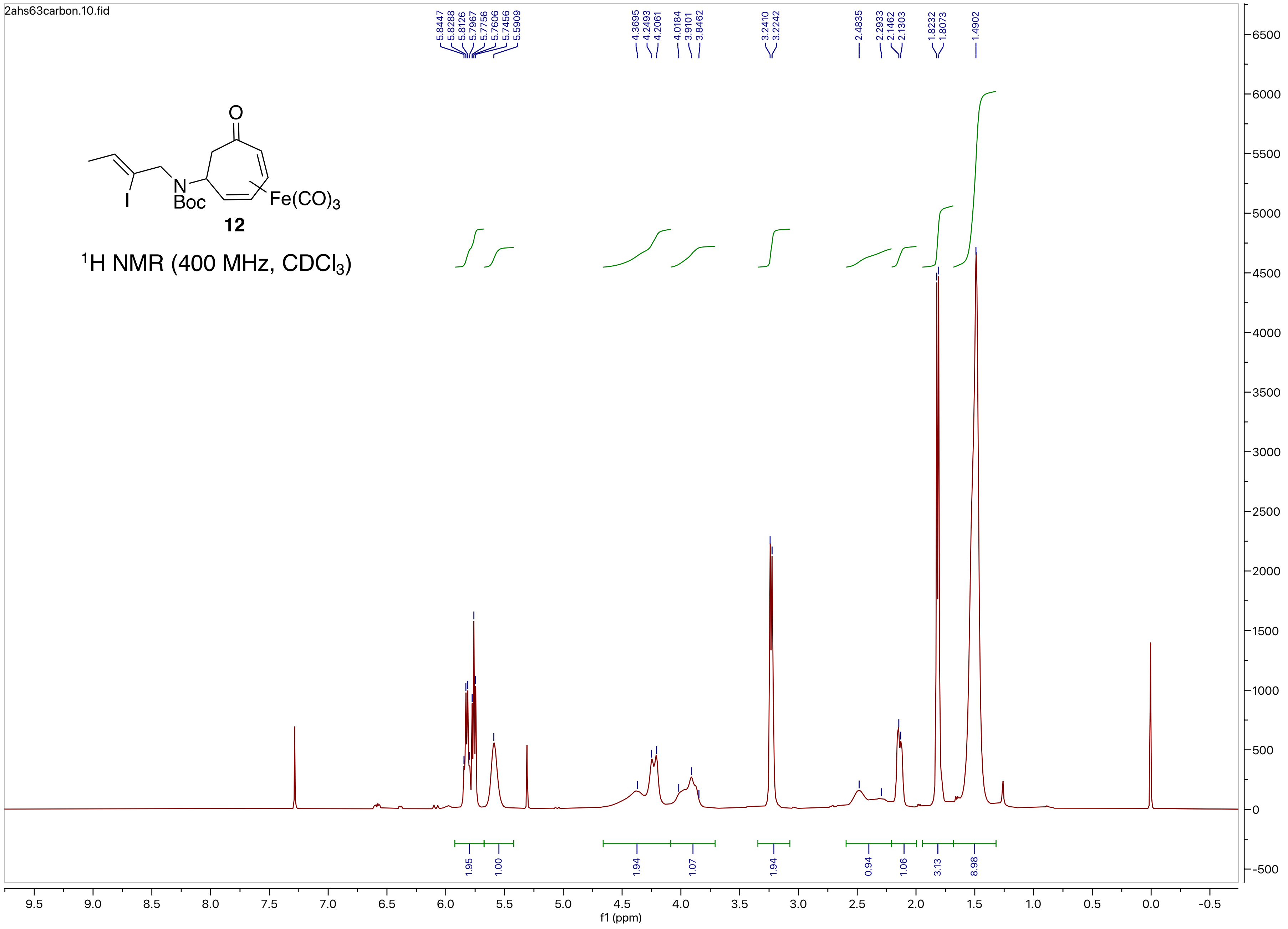
2ahs63carbon.10.fid
5.8447 5.8288 5.8126 5.7967 5.7756 5.7606 5.7456 5.5909
4.3695 4.2493 4.2061 4.0184 3.9101 3.8462
3.2410 3.2242
2.4835 2.2933 2.1462 2.1303
1.8232 1.8073
1.4902
O
N
Boc
I
Fe(CO)3
12
1H NMR (400 MHz, CDCl3)
1.95 1.00 1.94 1.07 1.94 0.94 1.06 3.13 8.98
f1 (ppm)

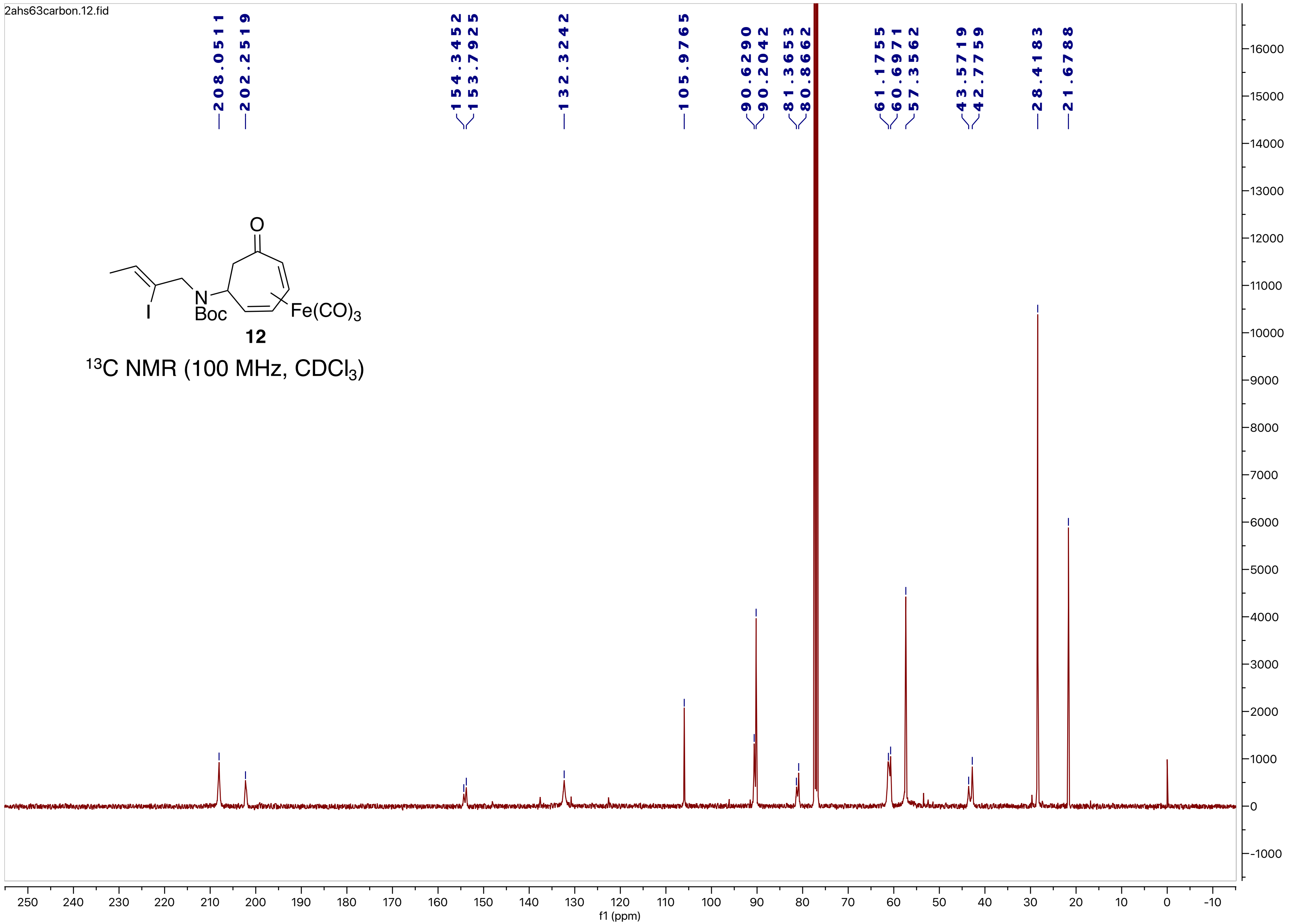
2ahs63carbon.12.fid
208.0511
202.2519
154.3452
153.7925
132.3242
105.9765
90.6290
90.2042
81.3653
80.8662
61.1755
60.6971
57.3562
43.5719
42.7759
28.4183
21.6788
O
N
Boc
I
Fe(CO)3
12
13C NMR (100 MHz, CDCl3)
f1 (ppm)

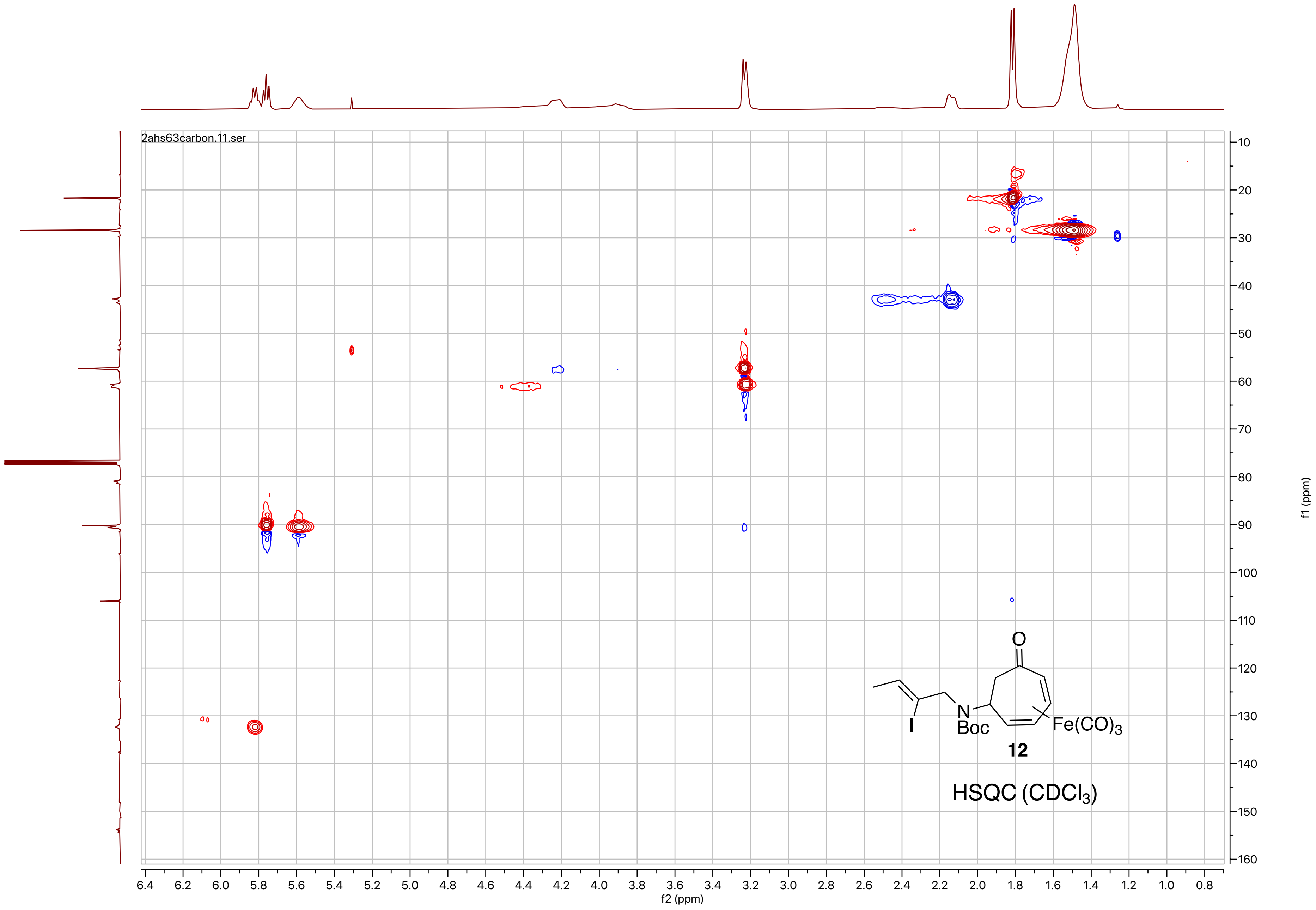

2ahs63carbon.11.ser
f2 (ppm)
f1 (ppm)
N
Boc
I
O
Fe(CO)3
12
HSQC (CDCl3)

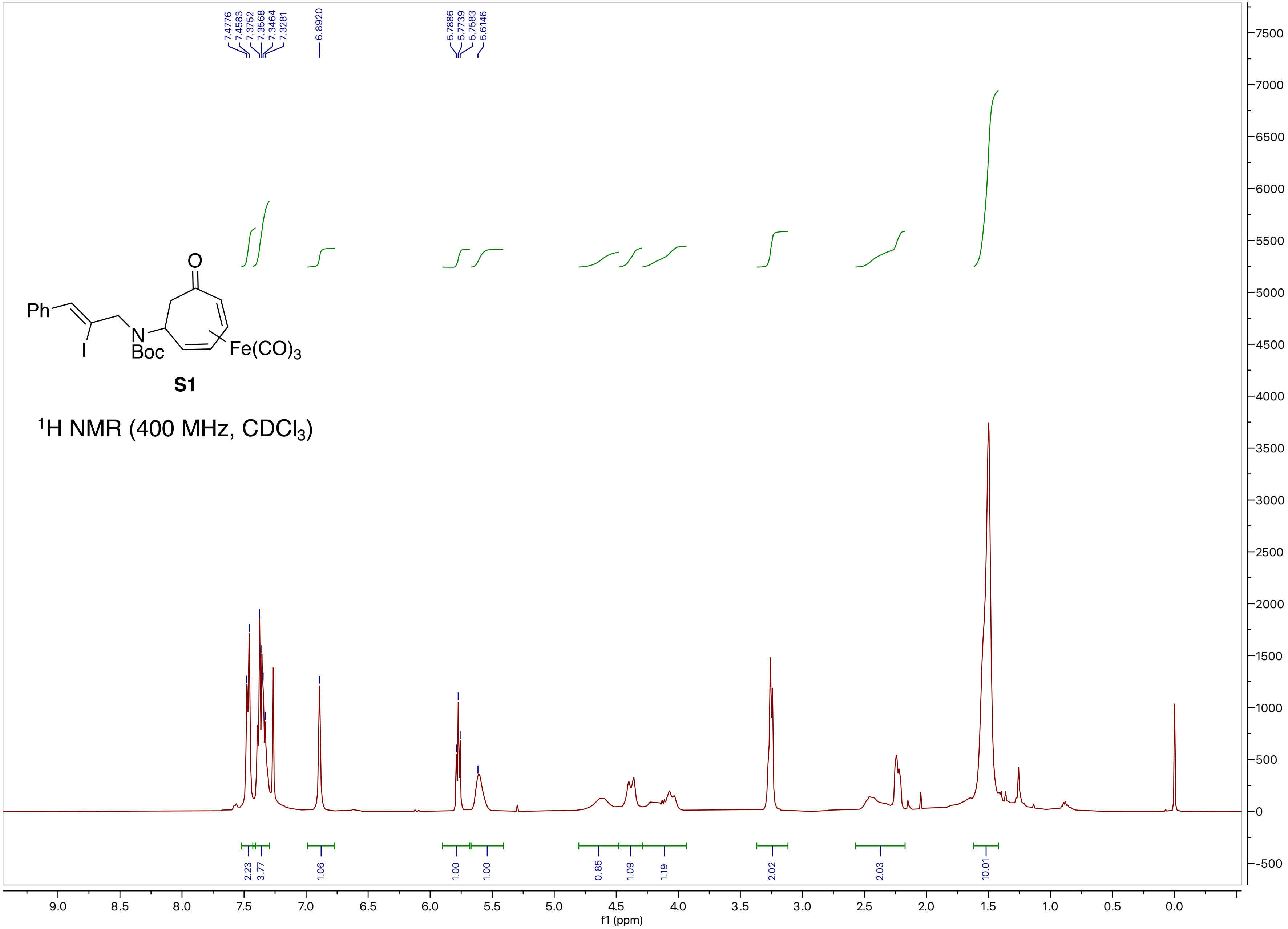

S1

^{1}H NMR (400 MHz, $CDCl_3$)

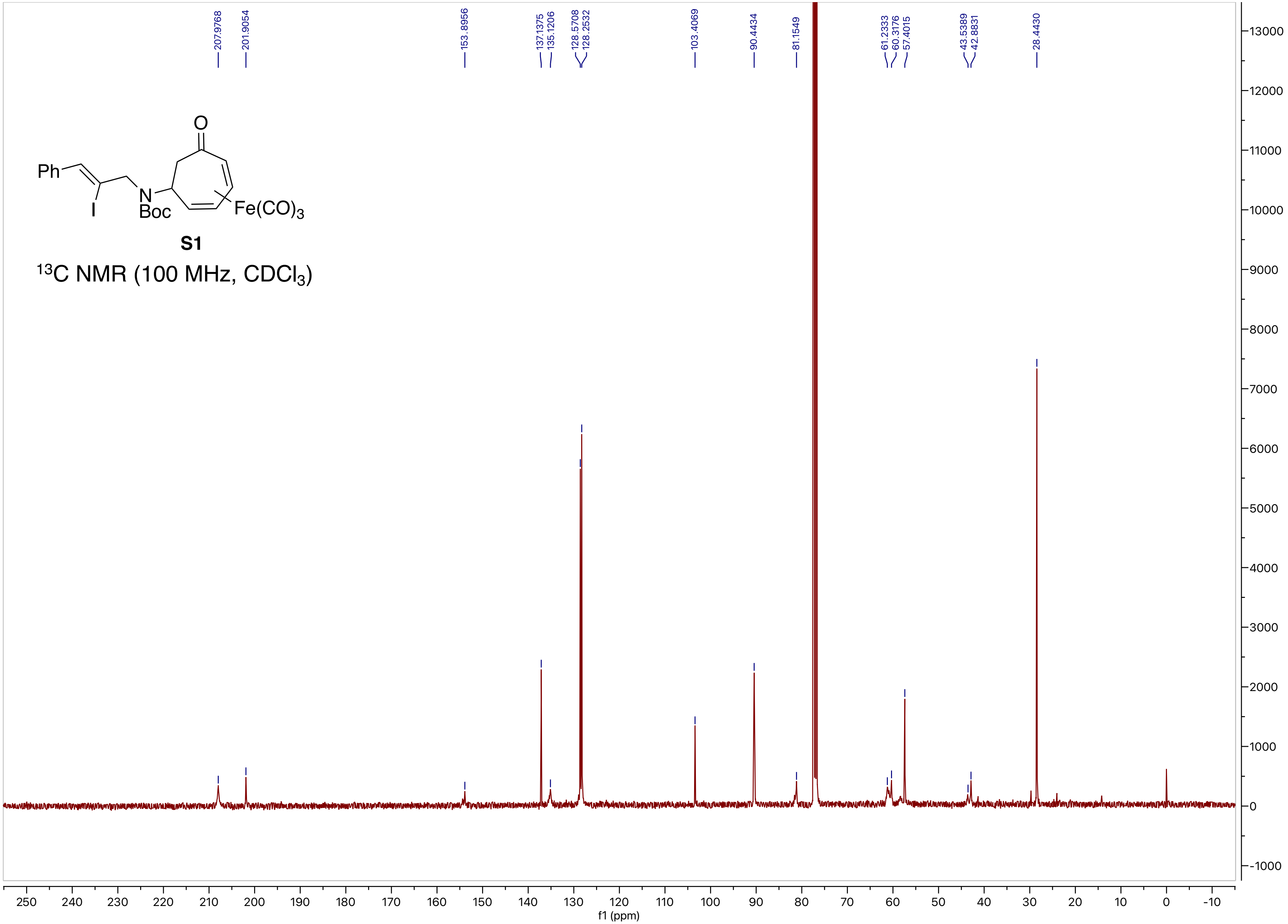
207.9768
201.9054
153.8956
137.1375
135.1206
128.5708
128.2532
103.4069
90.4434
81.1549
61.2333
60.3176
57.4015
43.5389
42.8831
28.4430
Ph
I
N
Boc
O
Fe(CO)3
S1
13C NMR (100 MHz, CDCl3)
f1 (ppm)

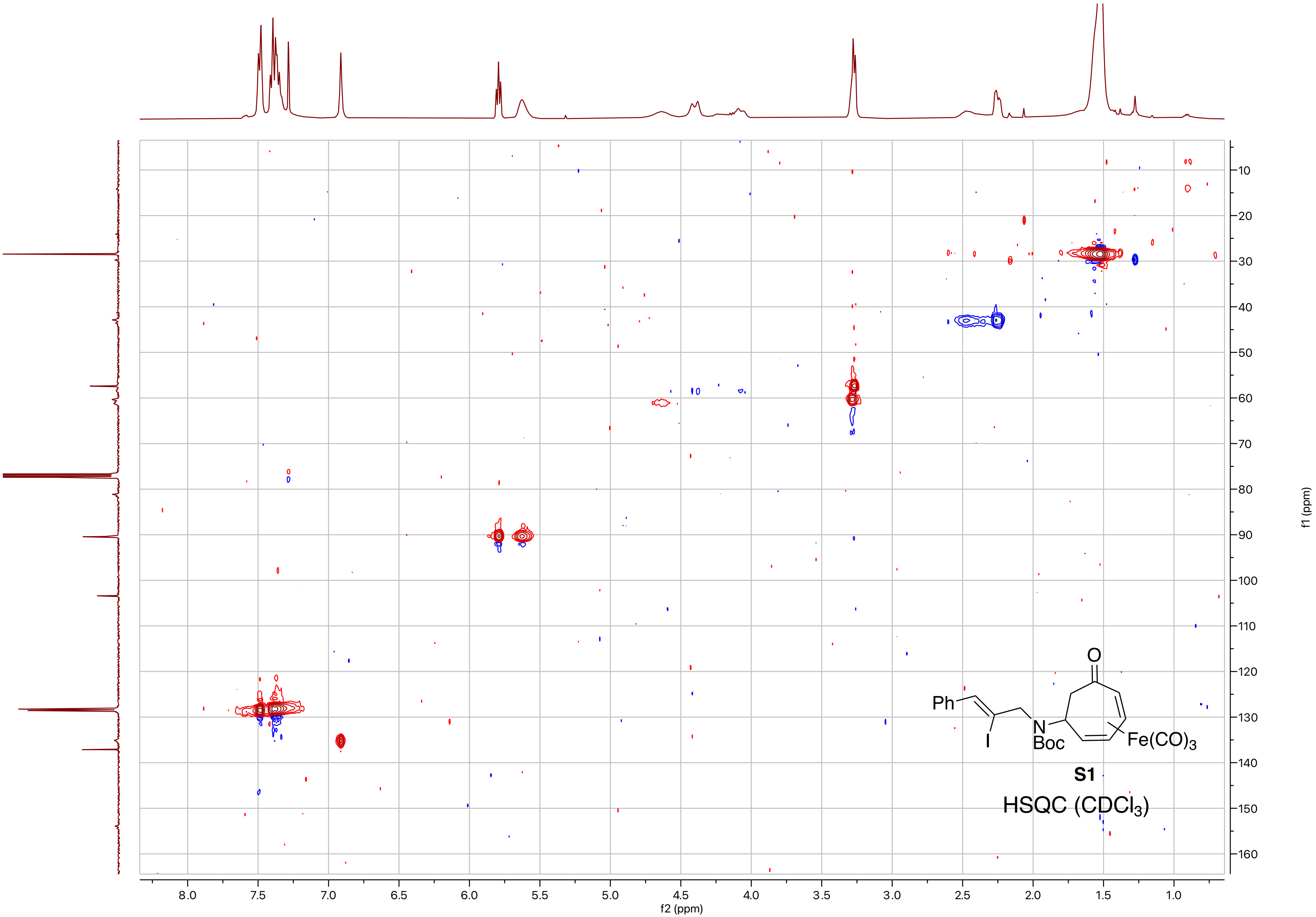

Ph
I
N
Boc
O
Fe(CO)3
S1
HSQC (CDCl3)
f2 (ppm)
f1 (ppm)
8.0 7.5 7.0 6.5 6.0 5.5 5.0 4.5 4.0 3.5 3.0 2.5 2.0 1.5 1.0
10 20 30 40 50 60 70 80 90 100 110 120 130 140 150 160

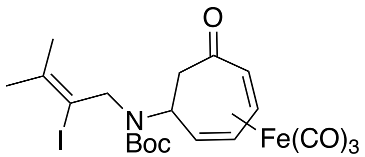

S2

^{1}H NMR (400 MHz, $CDCl_3$)

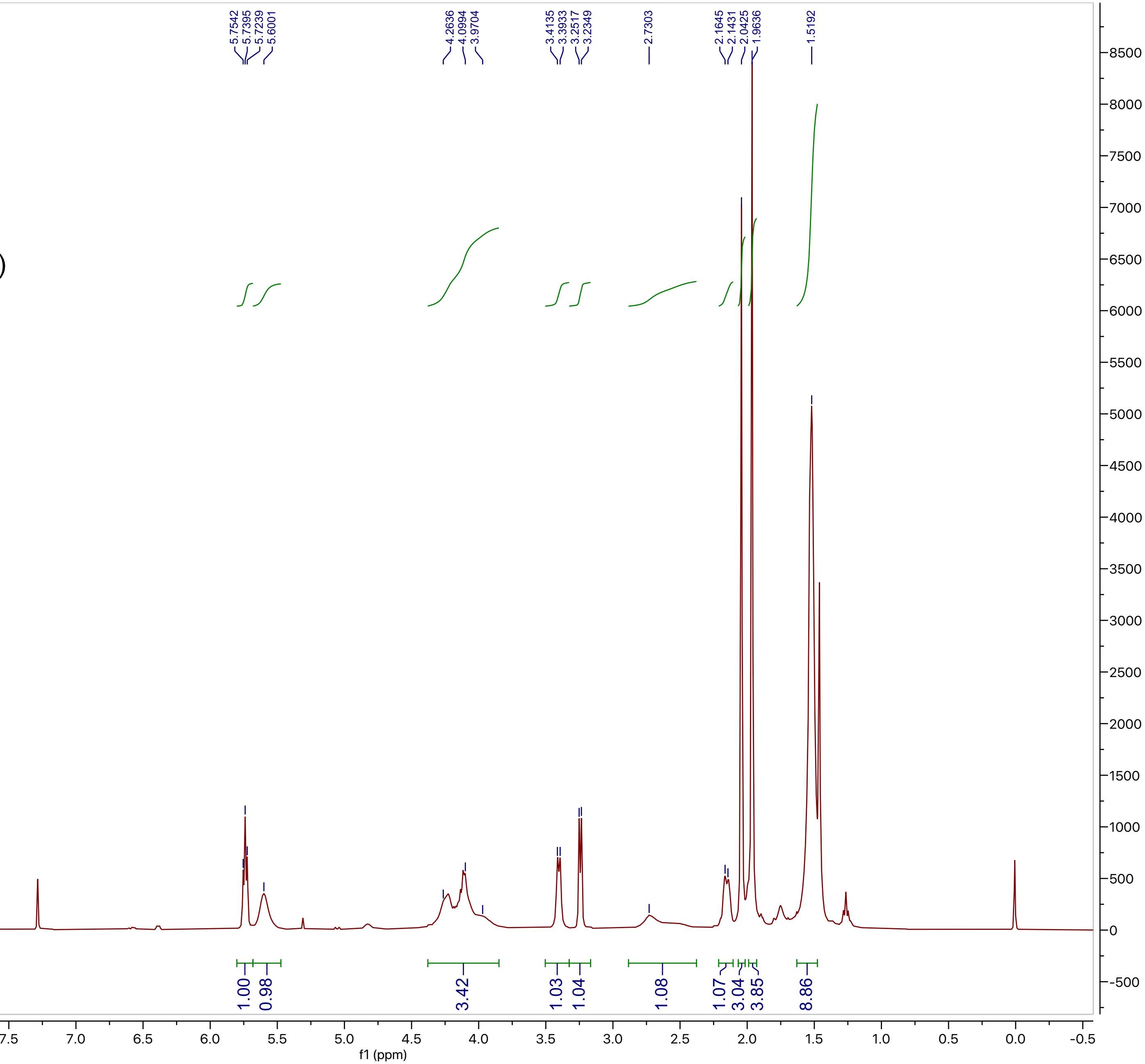

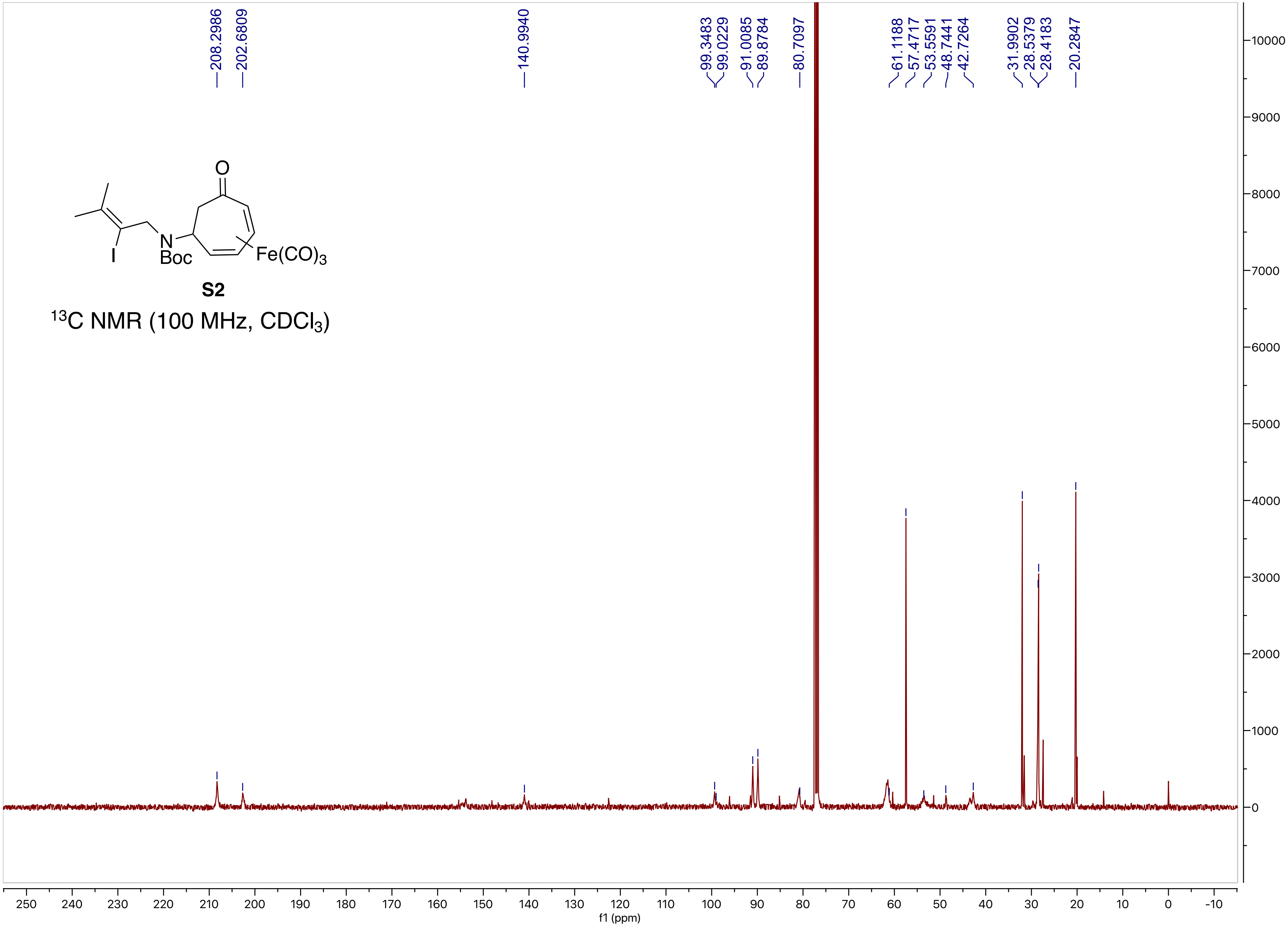

S2

^{13}C NMR (100 MHz, $CDCl_3$)

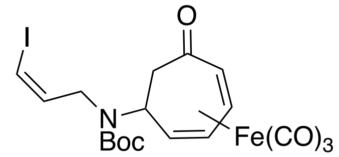

S3

^{1}H NMR (400 MHz, $CDCl_3$)

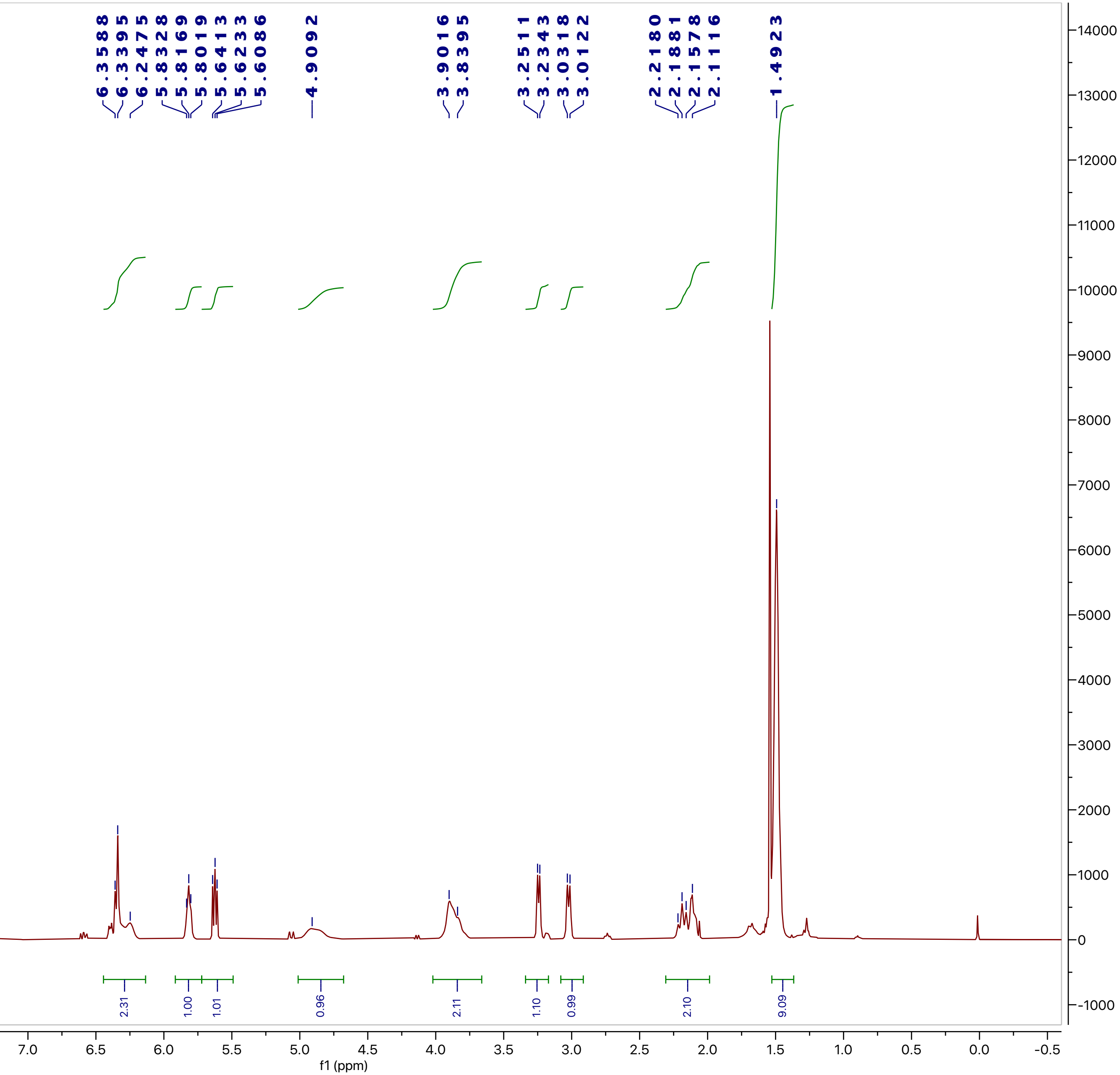

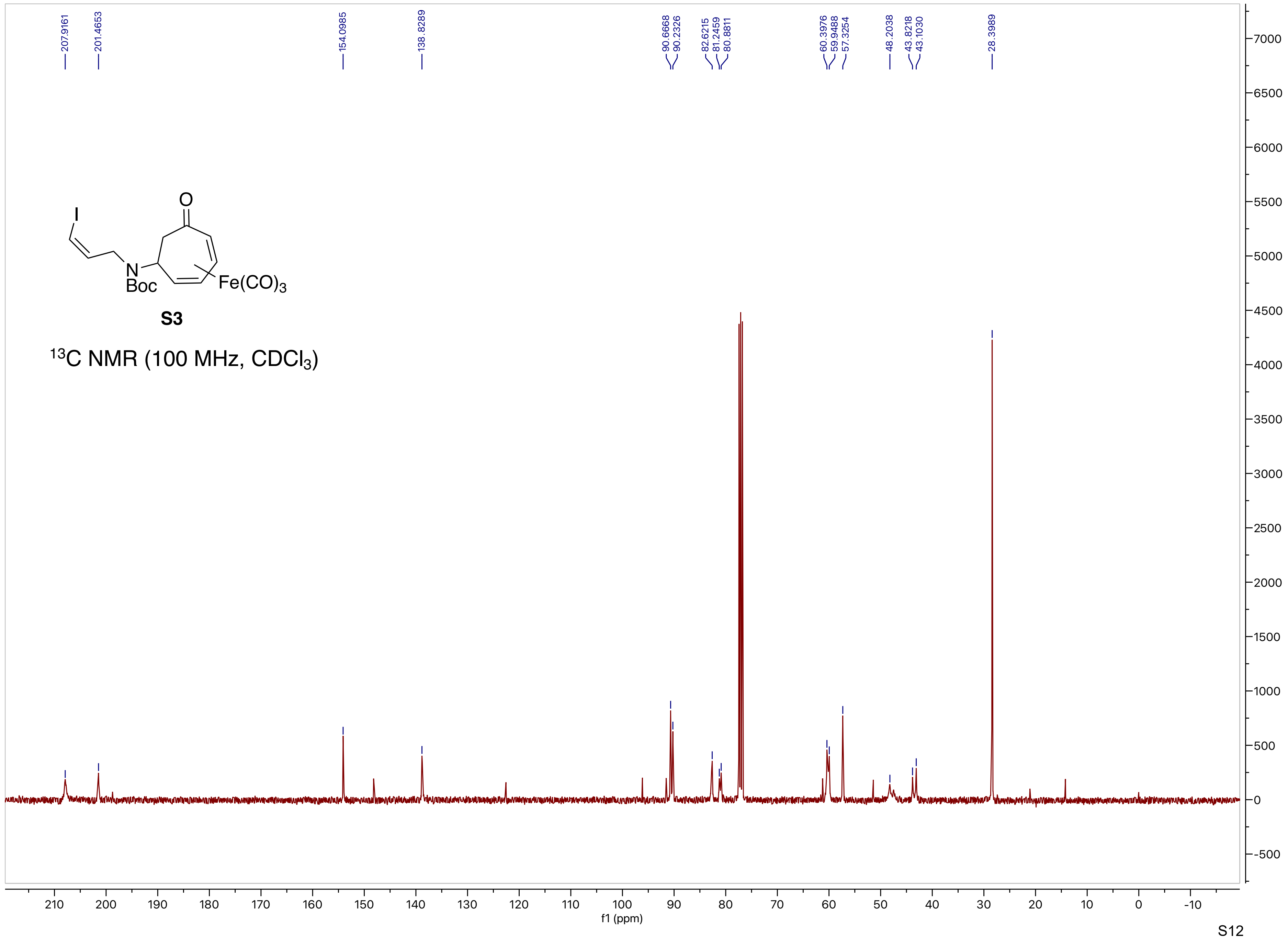
207.9161
201.4653
154.0985
138.8289
90.6668
90.2326
82.6215
81.2459
80.8811
60.3976
59.9488
57.3254
48.2038
43.8218
43.1030
28.3989
I
O
N
Boc
Fe(CO)3
S3
13C NMR (100 MHz, CDCl3)
f1 (ppm)

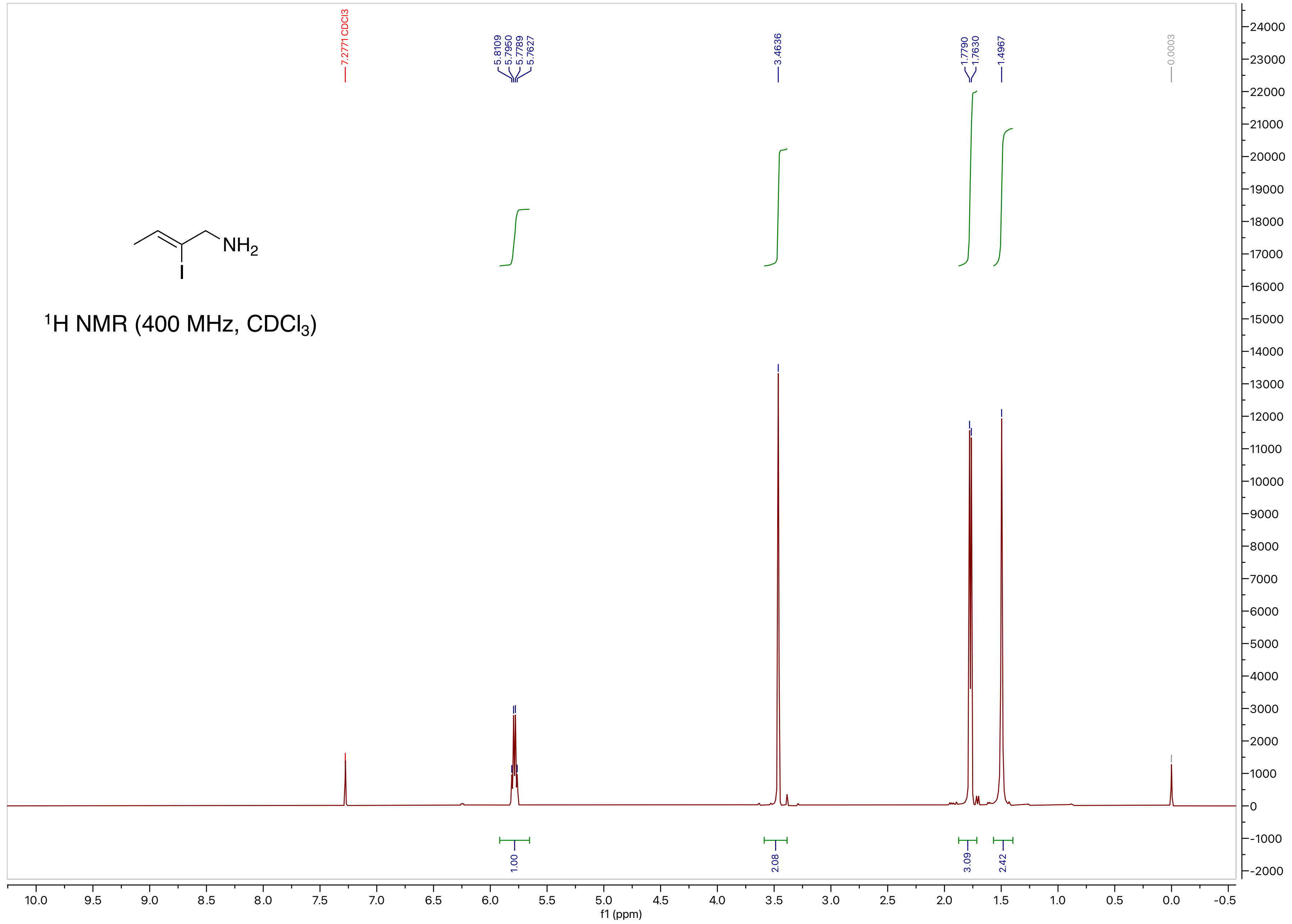
7.2771 CDCl3
5.8109
5.7950
5.7789
5.7627
3.4636
1.7790
1.7630
1.4967
0.0003
NH2
I
1H NMR (400 MHz, CDCl3)
1.00
2.08
3.09
2.42
f1 (ppm)

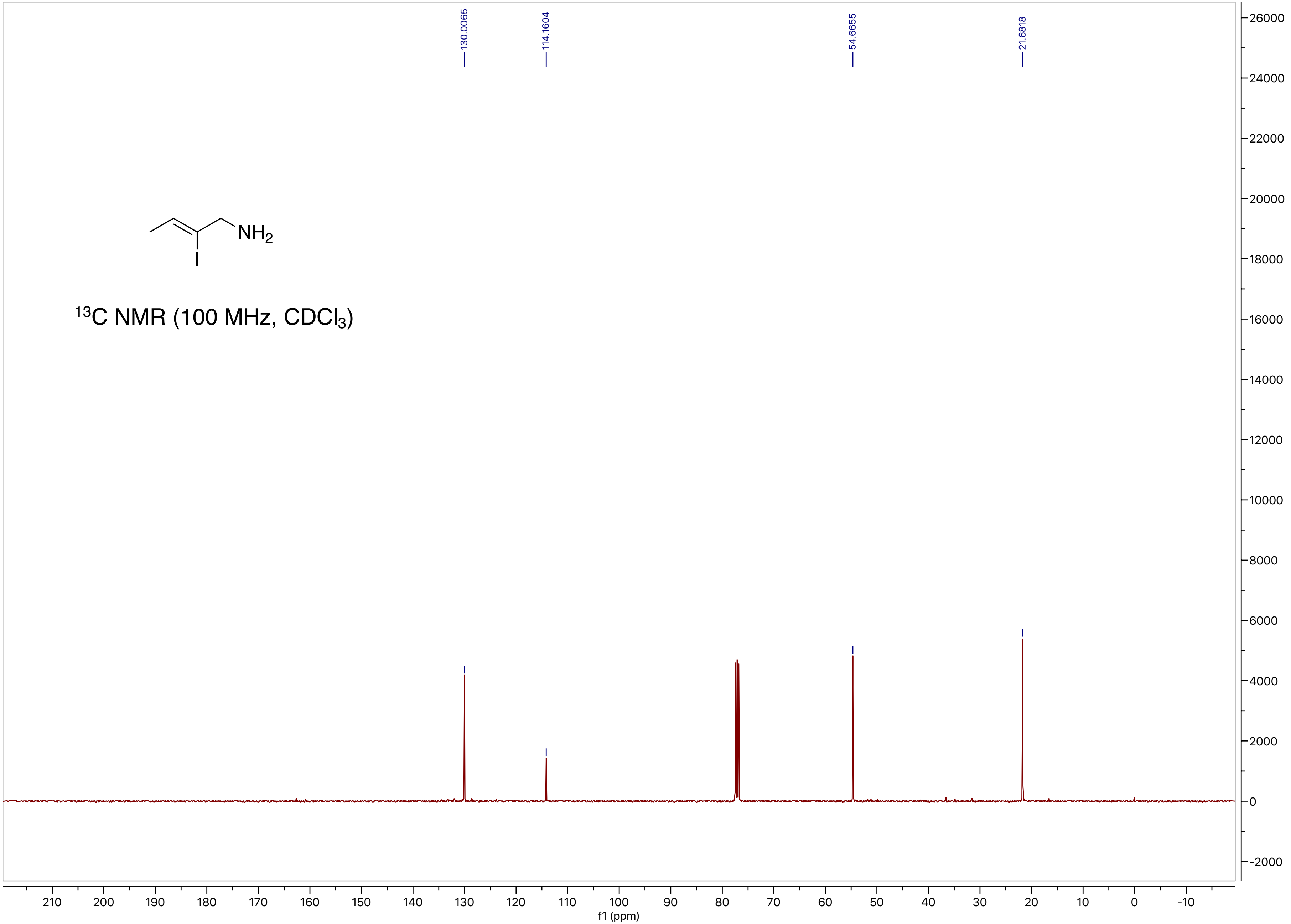

^{13}C NMR (100 MHz, $CDCl_3$)

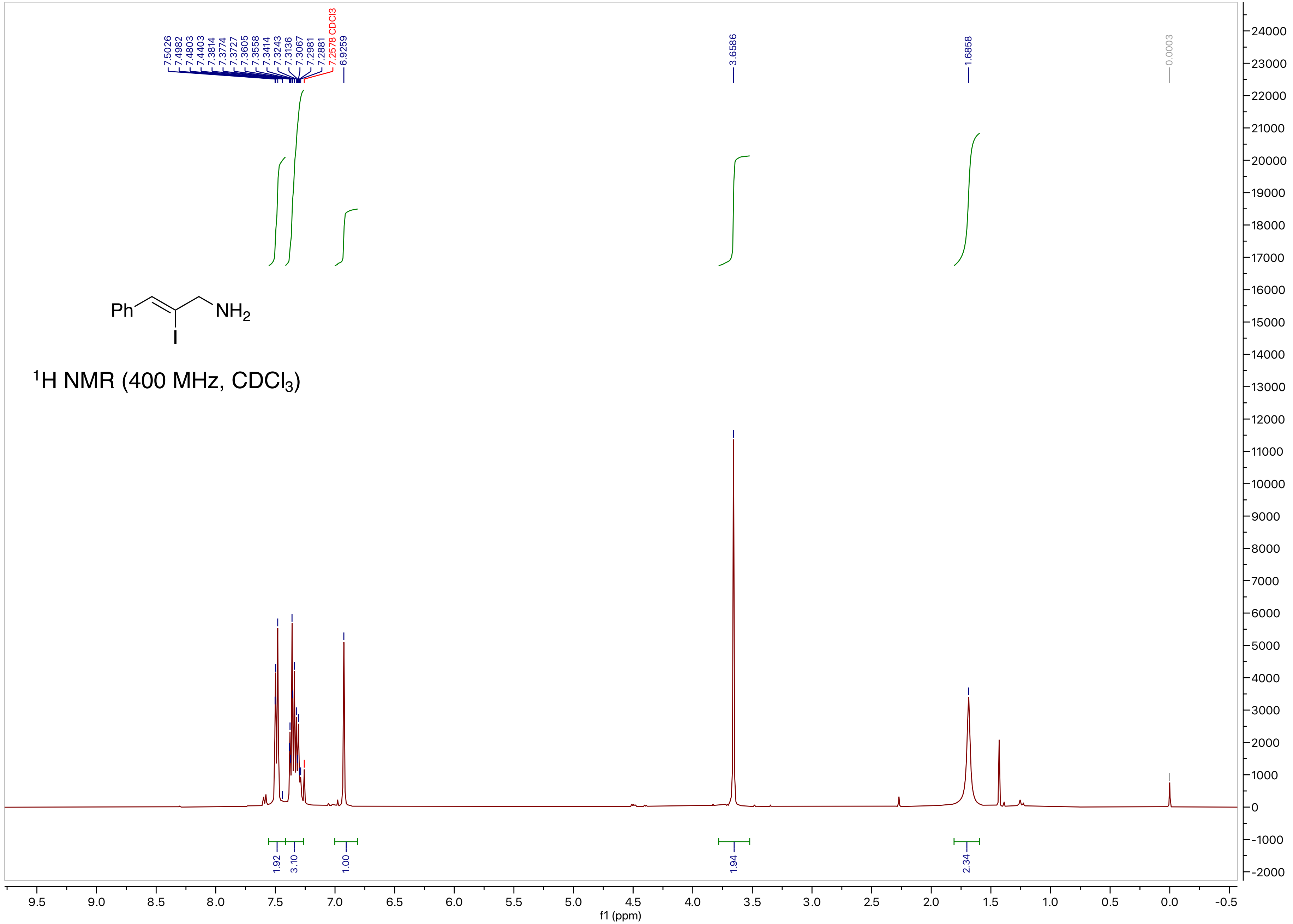
7.5026
7.4982
7.4803
7.4403
7.3814
7.3774
7.3727
7.3605
7.3558
7.3414
7.3243
7.3136
7.3067
7.2981
7.2881
7.2578 CDCl3
6.9259
3.6586
1.6858
0.0003
Ph
NH2
I
1H NMR (400 MHz, CDCl3)
1.92
3.10
1.00
1.94
2.34
f1 (ppm)

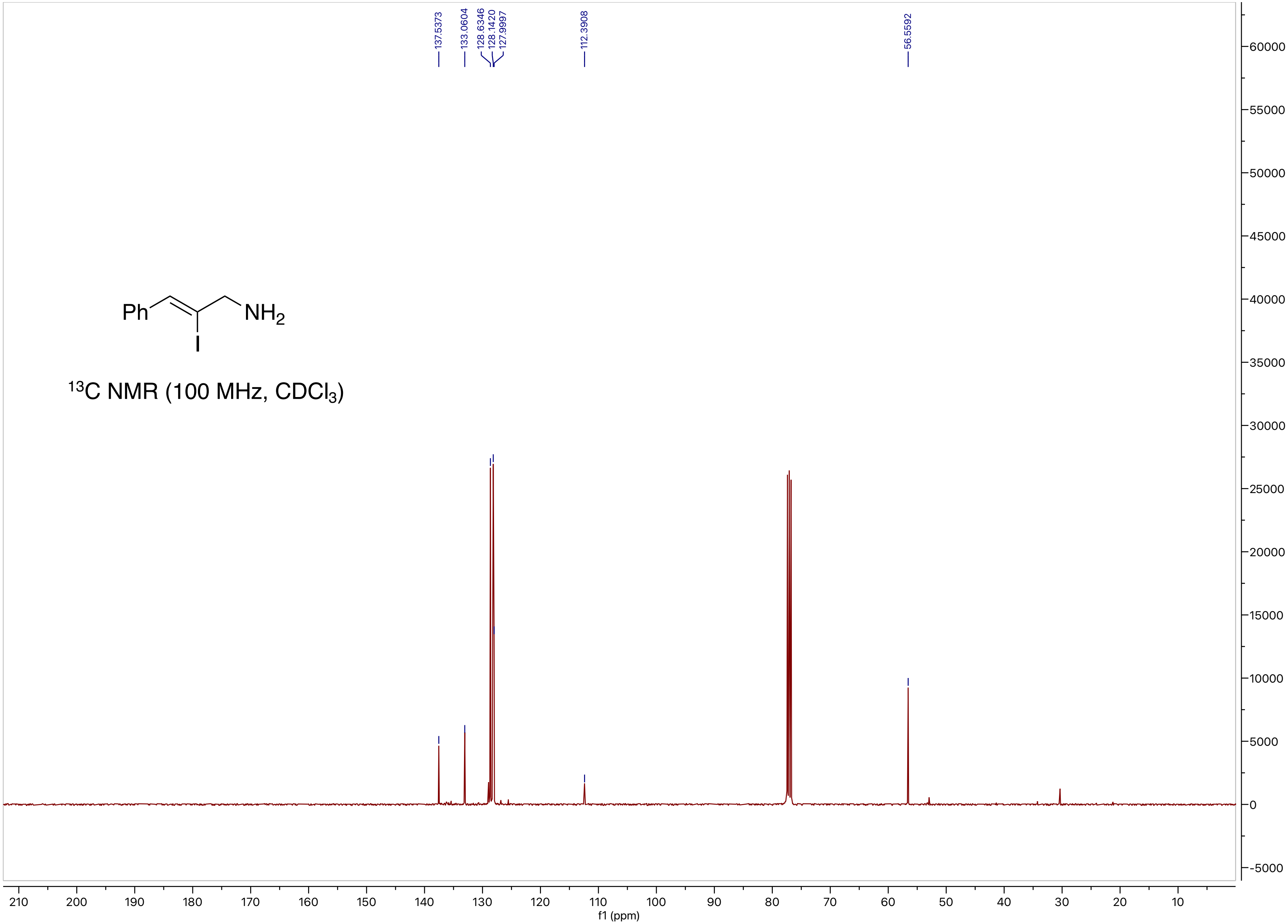
137.5373
133.0604
128.6346
128.1420
127.9997
112.3908
56.5592
Ph
NH2
I
13C NMR (100 MHz, CDCl3)
f1 (ppm)

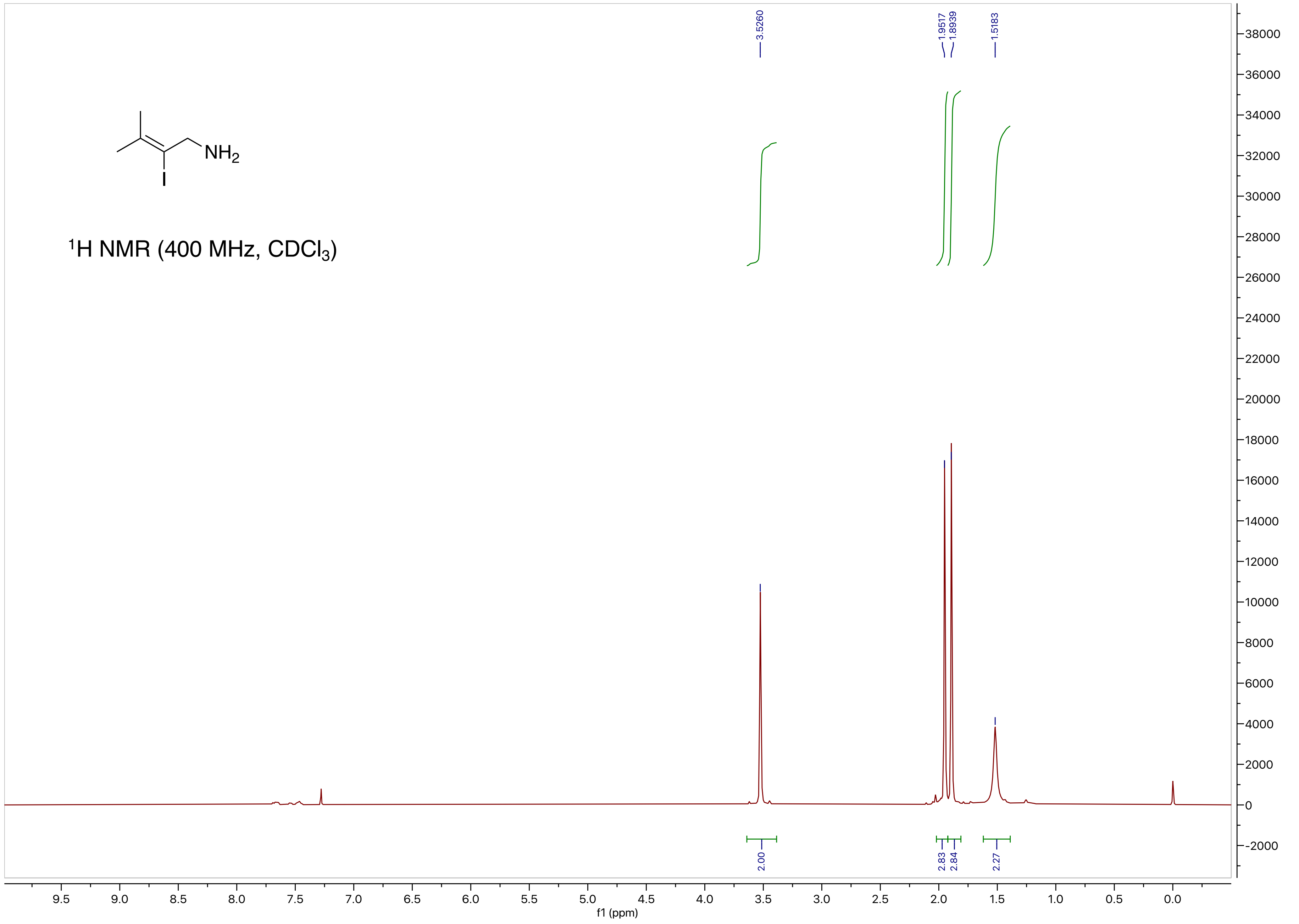

NH2
I
1H NMR (400 MHz, CDCl3)
3.5260
1.9517
1.8939
1.5183
2.00
2.83
2.84
2.27
f1 (ppm)

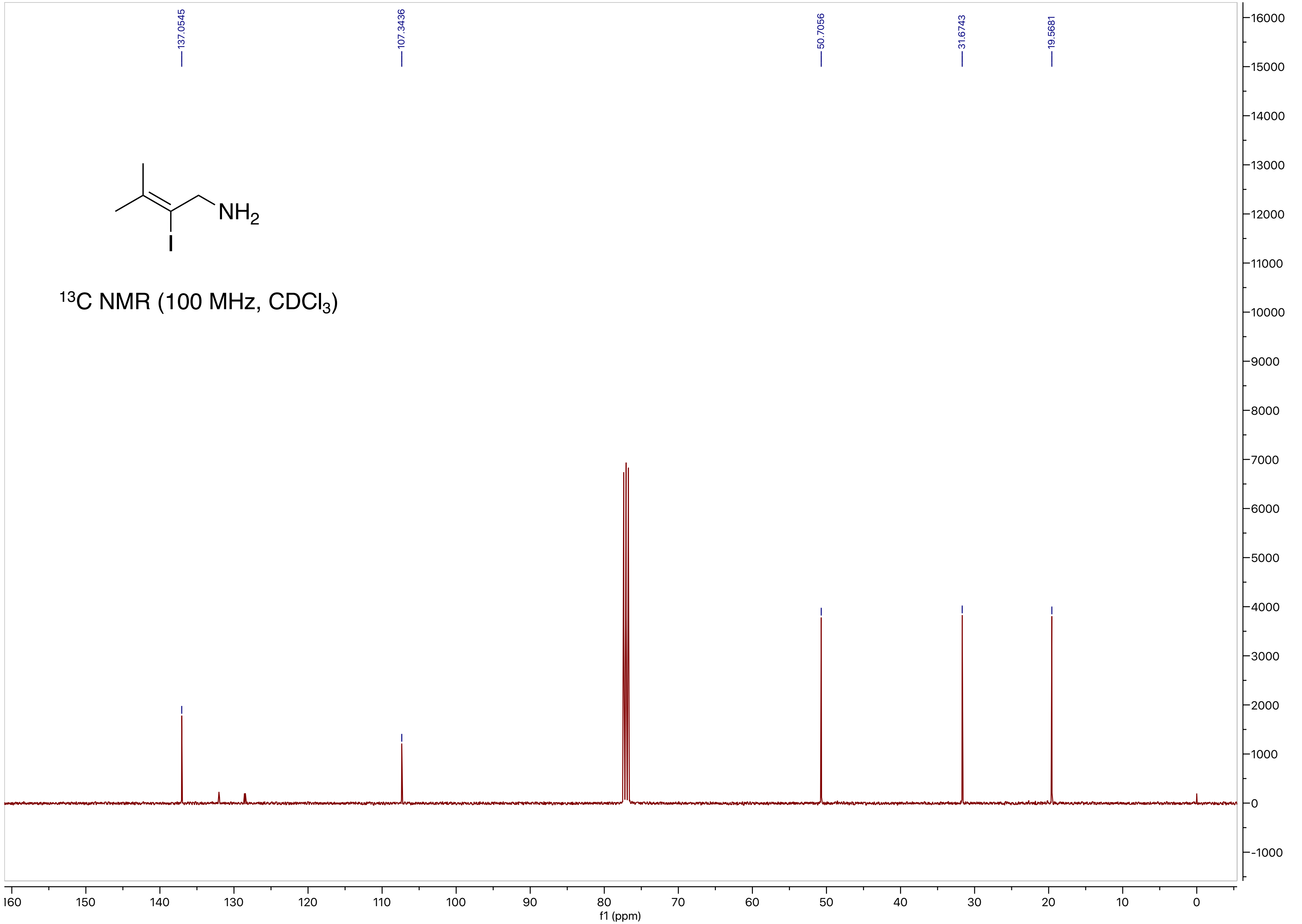
137.0545
107.3436
50.7056
31.6743
19.5681
NH2
I
13C NMR (100 MHz, CDCl3)
f1 (ppm)
160 150 140 130 120 110 100 90 80 70 60 50 40 30 20 10 0
16000 15000 14000 13000 12000 11000 10000 9000 8000 7000 6000 5000 4000 3000 2000 1000 0 -1000

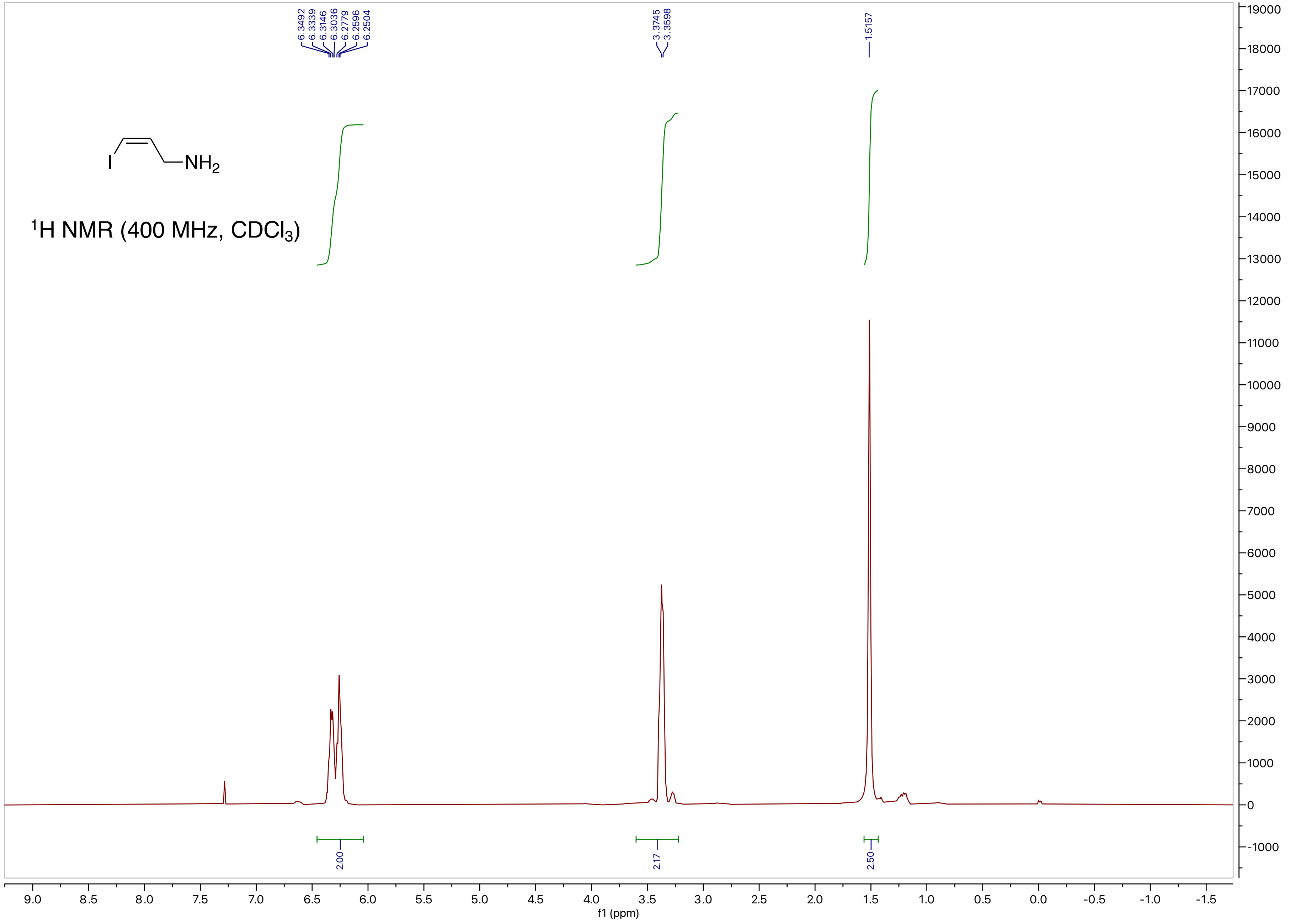

NH2
1H NMR (400 MHz, CDCl3)
6.3492
6.3339
6.3146
6.3036
6.2779
6.2596
6.2504
3.3745
3.3598
1.5157
2.00
2.17
2.50
f1 (ppm)

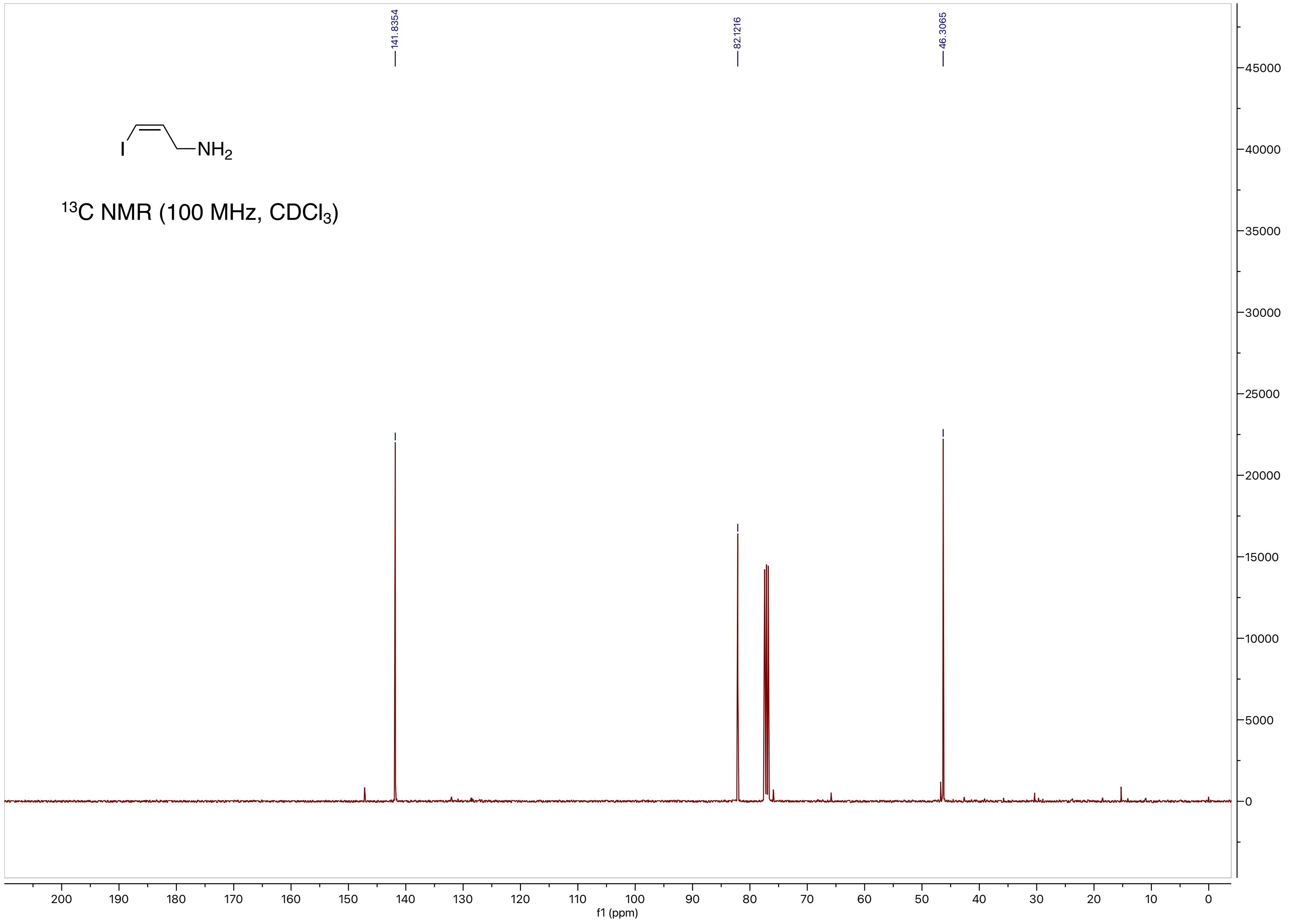

141.8354
82.1216
46.3065
I
NH2
13C NMR (100 MHz, CDCl3)
f1 (ppm)

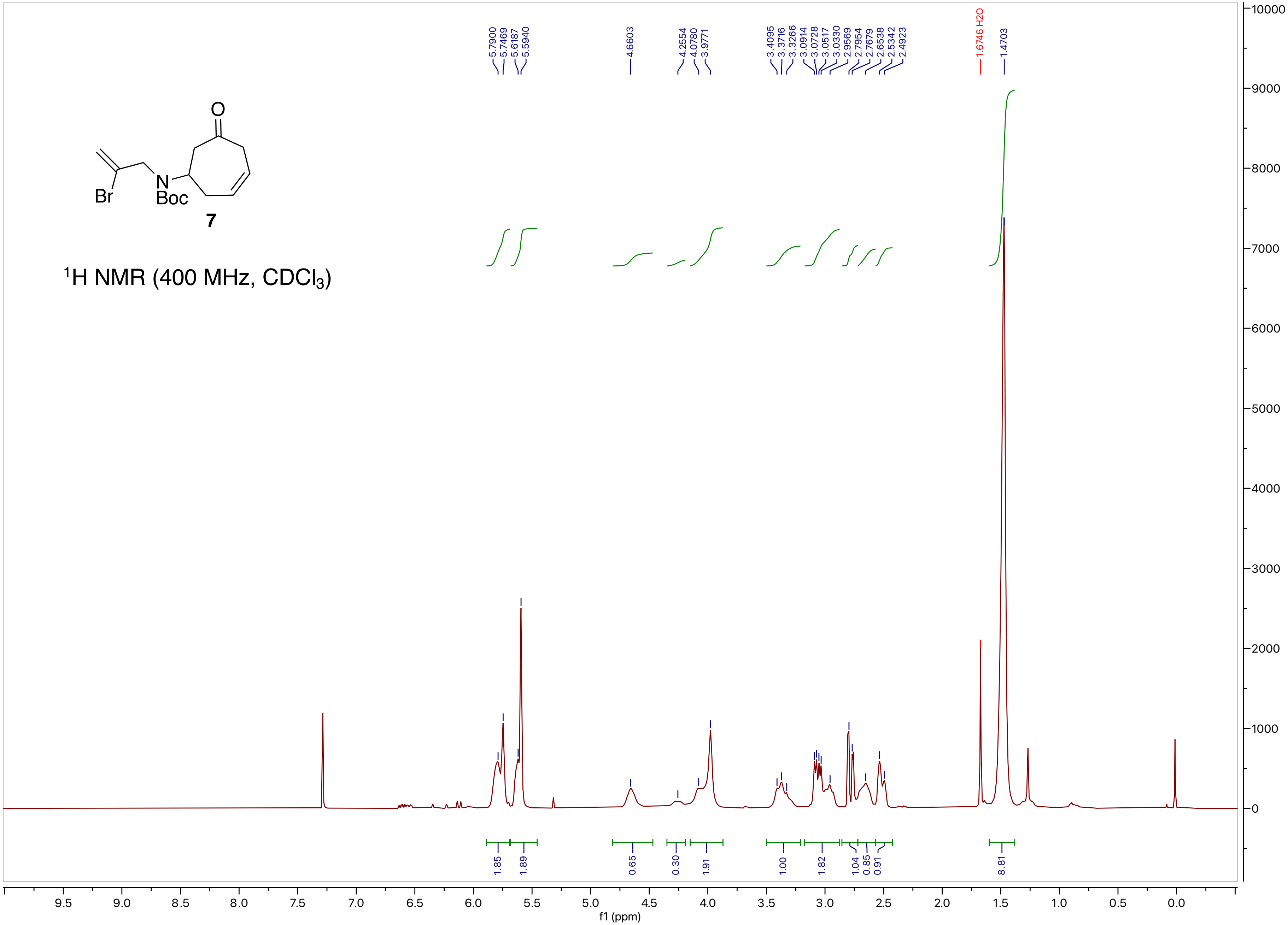
O
Br
N
Boc
7
1H NMR (400 MHz, CDCl3)
5.7900
5.7469
5.6187
5.5940
4.6603
4.2554
4.0780
3.9771
3.4095
3.3716
3.3266
3.0914
3.0728
3.0517
3.0330
2.9569
2.7954
2.7679
2.6538
2.5342
2.4923
1.6746 H2O
1.4703
1.85
1.89
0.65
0.30
1.91
1.00
1.82
1.04
0.85
0.91
8.81
f1 (ppm)

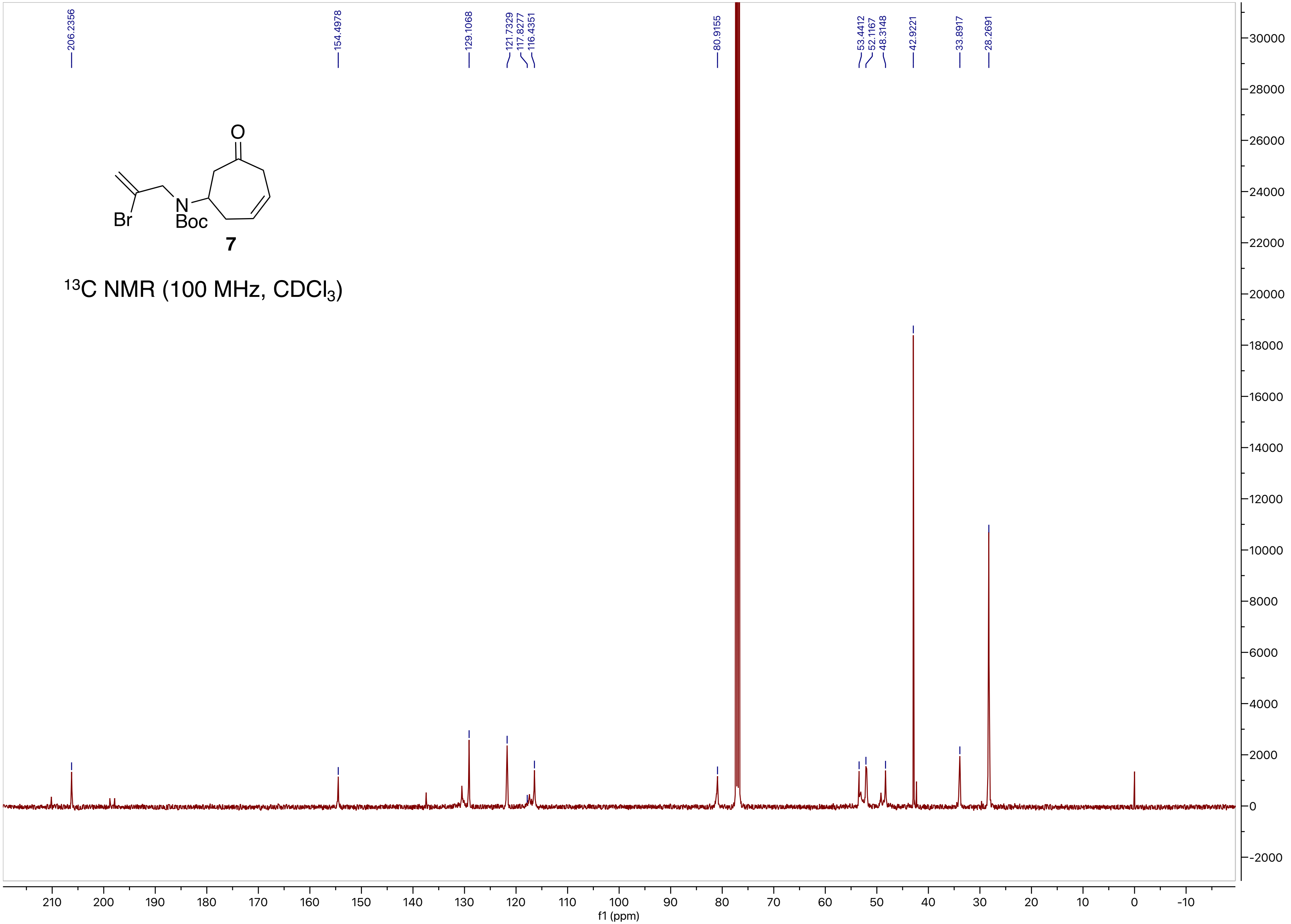
206.2356
154.4978
129.1068
121.7329
117.8277
116.4351
80.9155
53.4412
52.1167
48.3148
42.9221
33.8917
28.2691
O
N
Br
Boc
7
13C NMR (100 MHz, CDCl3)
f1 (ppm)

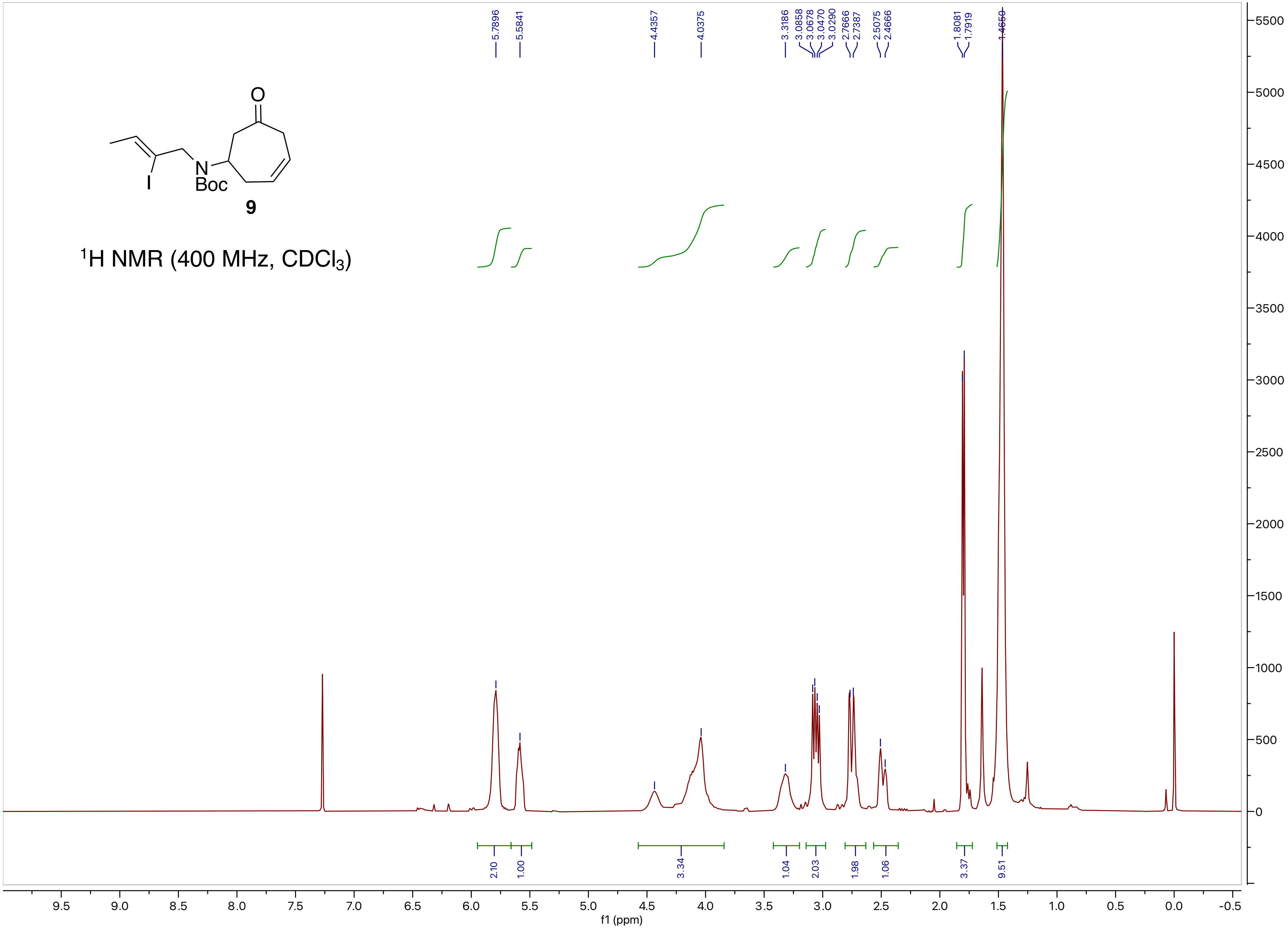

O
N
Boc
I
9
¹H NMR (400 MHz, CDCl₃)
5.7896
5.5841
4.4357
4.0375
3.3186
3.0858
3.0678
3.0470
3.0290
2.7666
2.7387
2.5075
2.4666
1.8081
1.7919
1.4650
2.10
1.00
3.34
1.04
2.03
1.98
1.06
3.37
9.51
f1 (ppm)

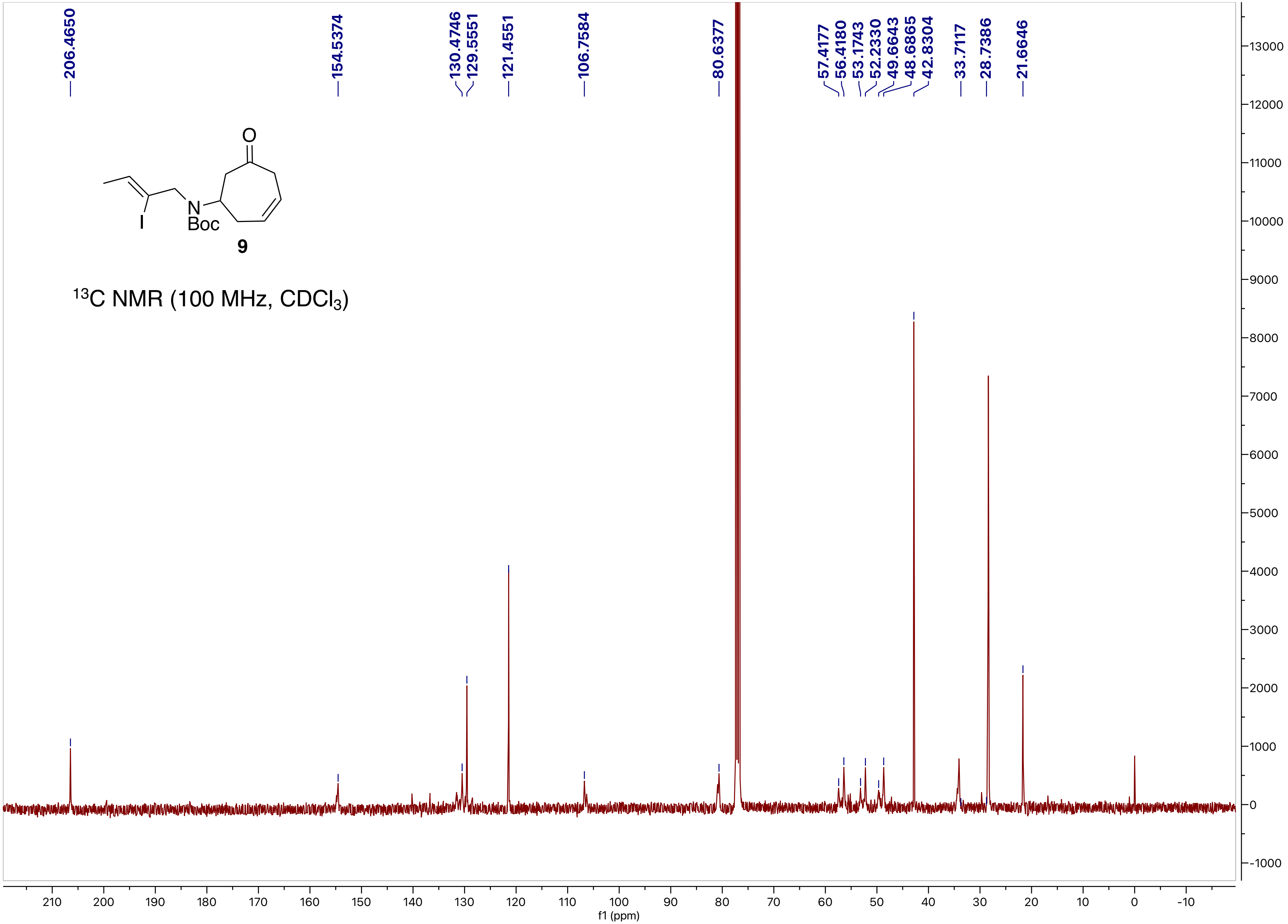
206.4650
154.5374
130.4746
129.5551
121.4551
106.7584
80.6377
57.4177
56.4180
53.1743
52.2330
49.6643
48.6865
42.8304
33.7117
28.7386
21.6646
O
N
Boc
I
9
^{13}C NMR (100 MHz, $CDCl_3$)
f1 (ppm)

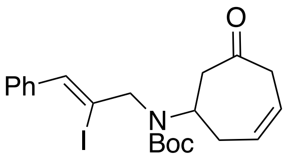

13

^{1}H NMR (400 MHz, $CDCl_3$)

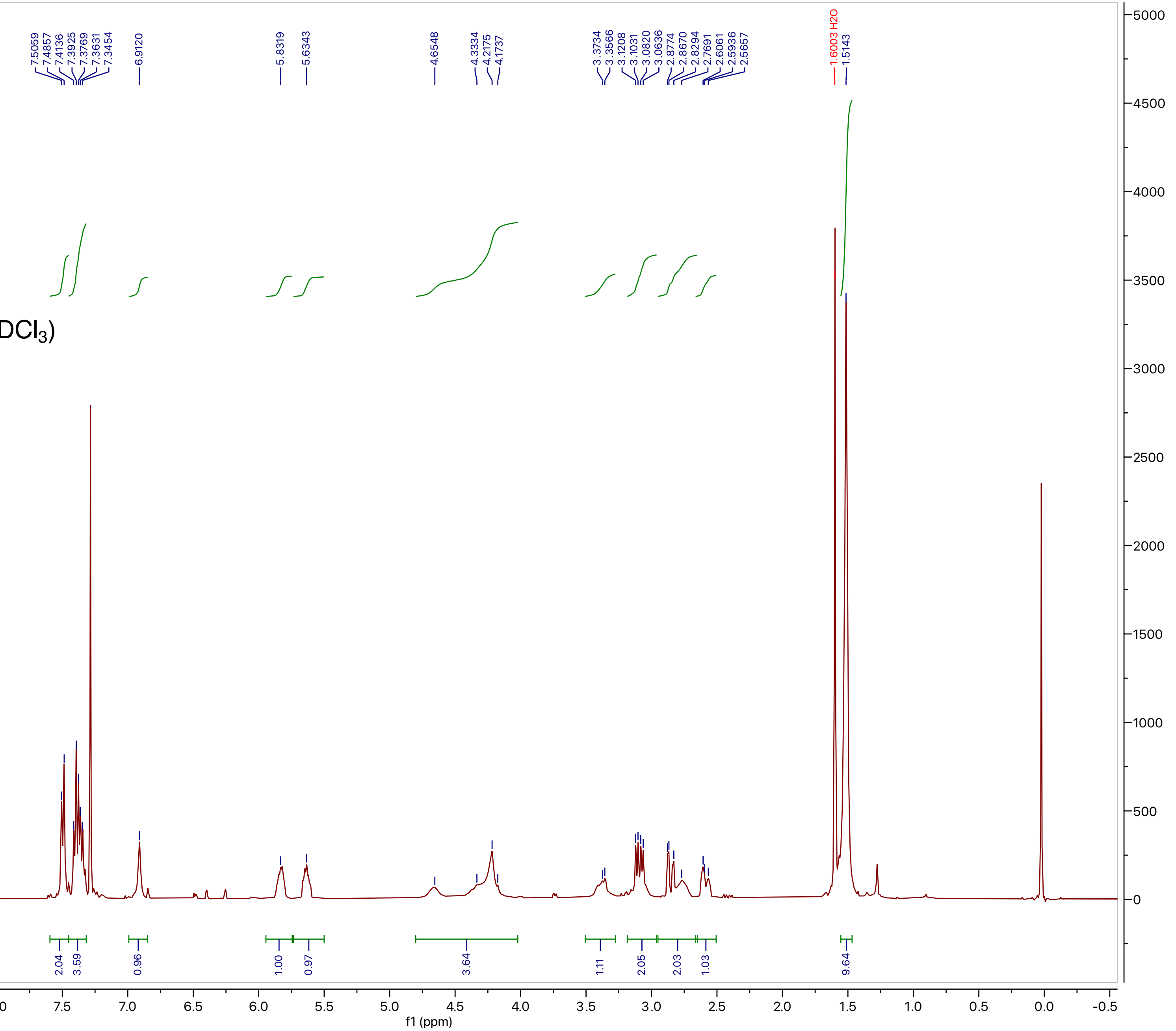

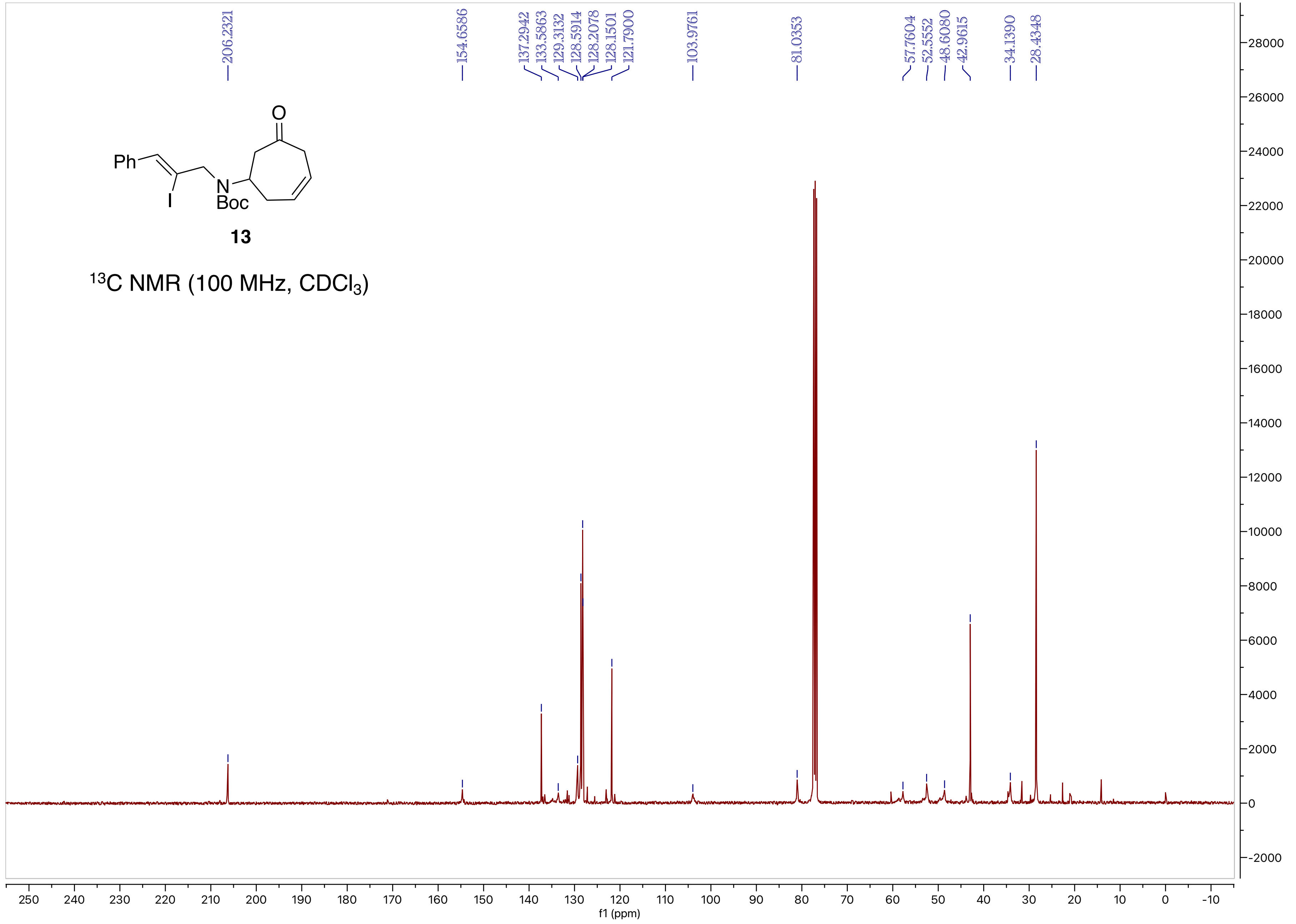
206.2321
154.6586
137.2942
133.5863
129.3132
128.5914
128.2078
128.1501
121.7900
103.9761
81.0353
57.7604
52.5552
48.6080
42.9615
34.1390
28.4348
Ph
I
N
Boc
O
13
^{13}C NMR (100 MHz, CDCl$_3$)
f1 (ppm)

15

^{1}H NMR (400 MHz, $CDCl_3$)

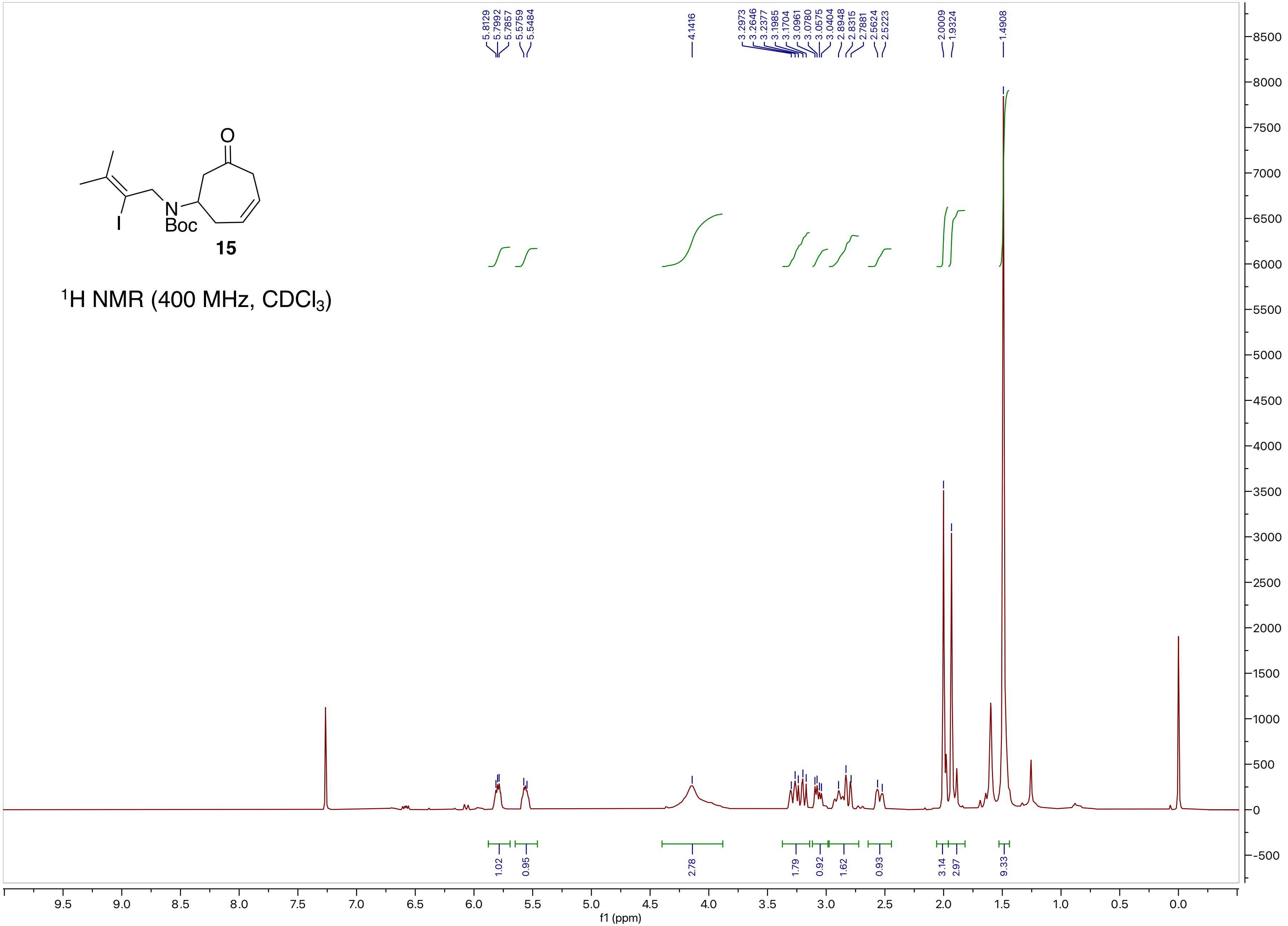

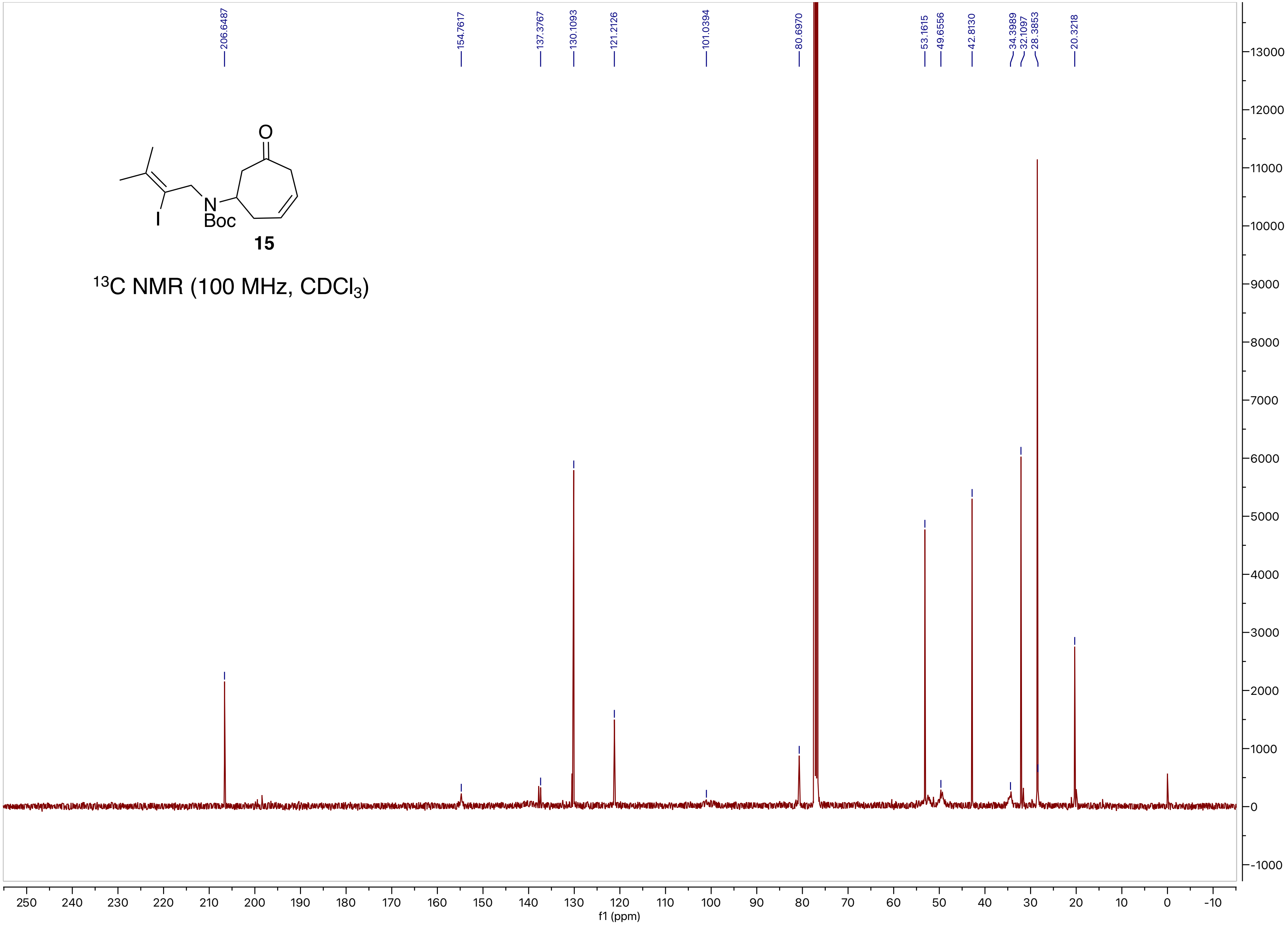
206.6487
154.7617
137.3767
130.1093
121.2126
101.0394
80.6970
53.1615
49.6556
42.8130
34.3989
32.1097
28.3853
20.3218
O
N
Boc
I
15
^{13}C NMR (100 MHz, $CDCl_3$)
f1 (ppm)

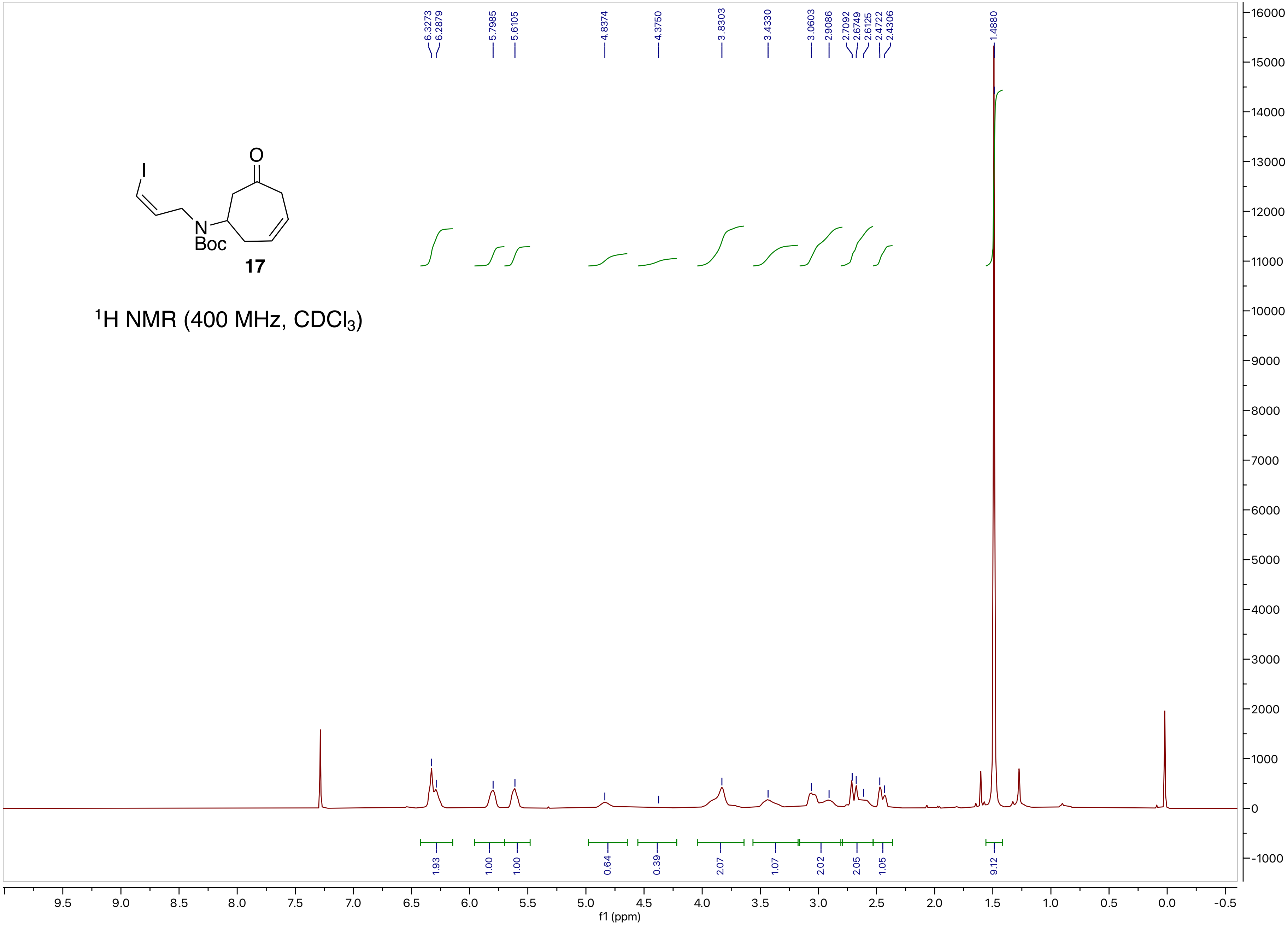
O
I
N
Boc
17
^{1}H NMR (400 MHz, $CDCl_3$)
6.3273
6.2879
5.7985
5.6105
4.8374
4.3750
3.8303
3.4330
3.0603
2.9086
2.7092
2.6749
2.6125
2.4722
2.4306
1.4880
1.93
1.00
1.00
0.64
0.39
2.07
1.07
2.02
2.05
1.05
9.12
f1 (ppm)

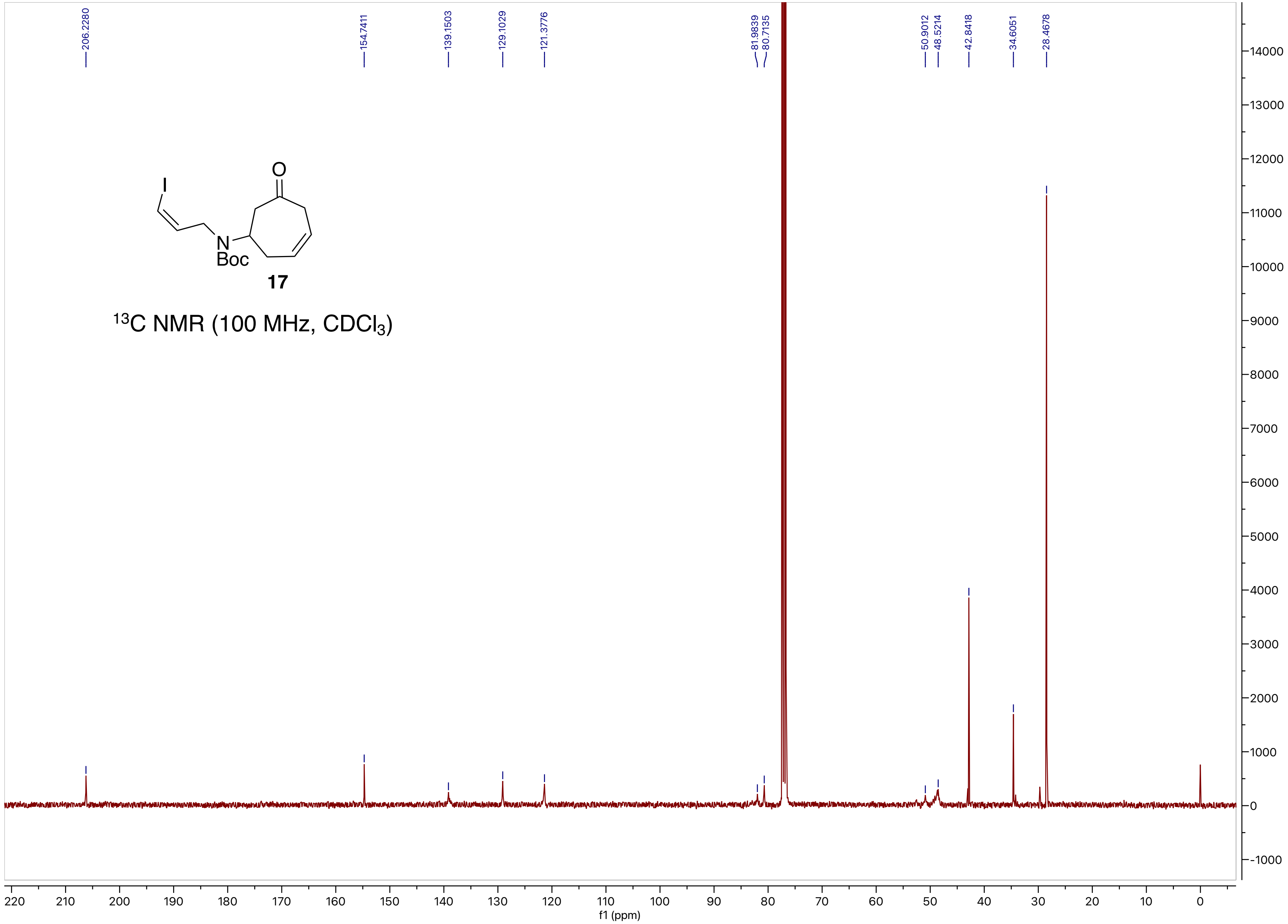
206.2280
154.7411
139.1503
129.1029
121.3776
81.9839
80.7135
50.9012
48.5214
42.8418
34.6051
28.4678
I
O
N
Boc
17
13C NMR (100 MHz, CDCl3)
14000
13000
12000
11000
10000
9000
8000
7000
6000
5000
4000
3000
2000
1000
0
-1000
220 210 200 190 180 170 160 150 140 130 120 110 100 90 80 70 60 50 40 30 20 10 0
f1 (ppm)

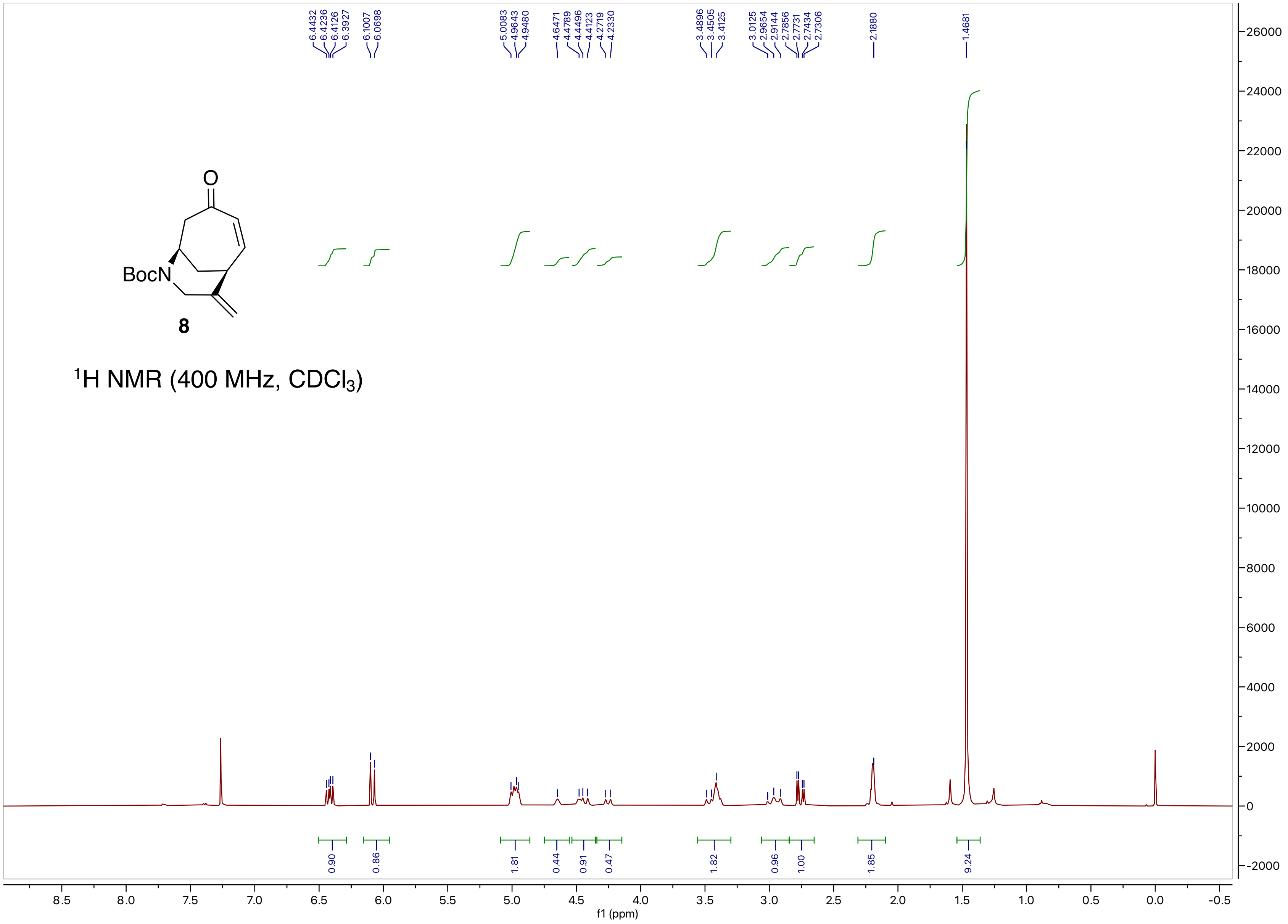

O
BocN
8
1H NMR (400 MHz, CDCl3)
6.4432
6.4236
6.4126
6.3927
6.1007
6.0698
5.0083
4.9643
4.9480
4.6471
4.4789
4.4496
4.4123
4.2719
4.2330
3.4896
3.4505
3.4125
3.0125
2.9654
2.9144
2.7856
2.7731
2.7434
2.7306
2.1880
1.4681
0.90
0.86
1.81
0.44
0.91
0.47
1.82
0.96
1.00
1.85
9.24
f1 (ppm)

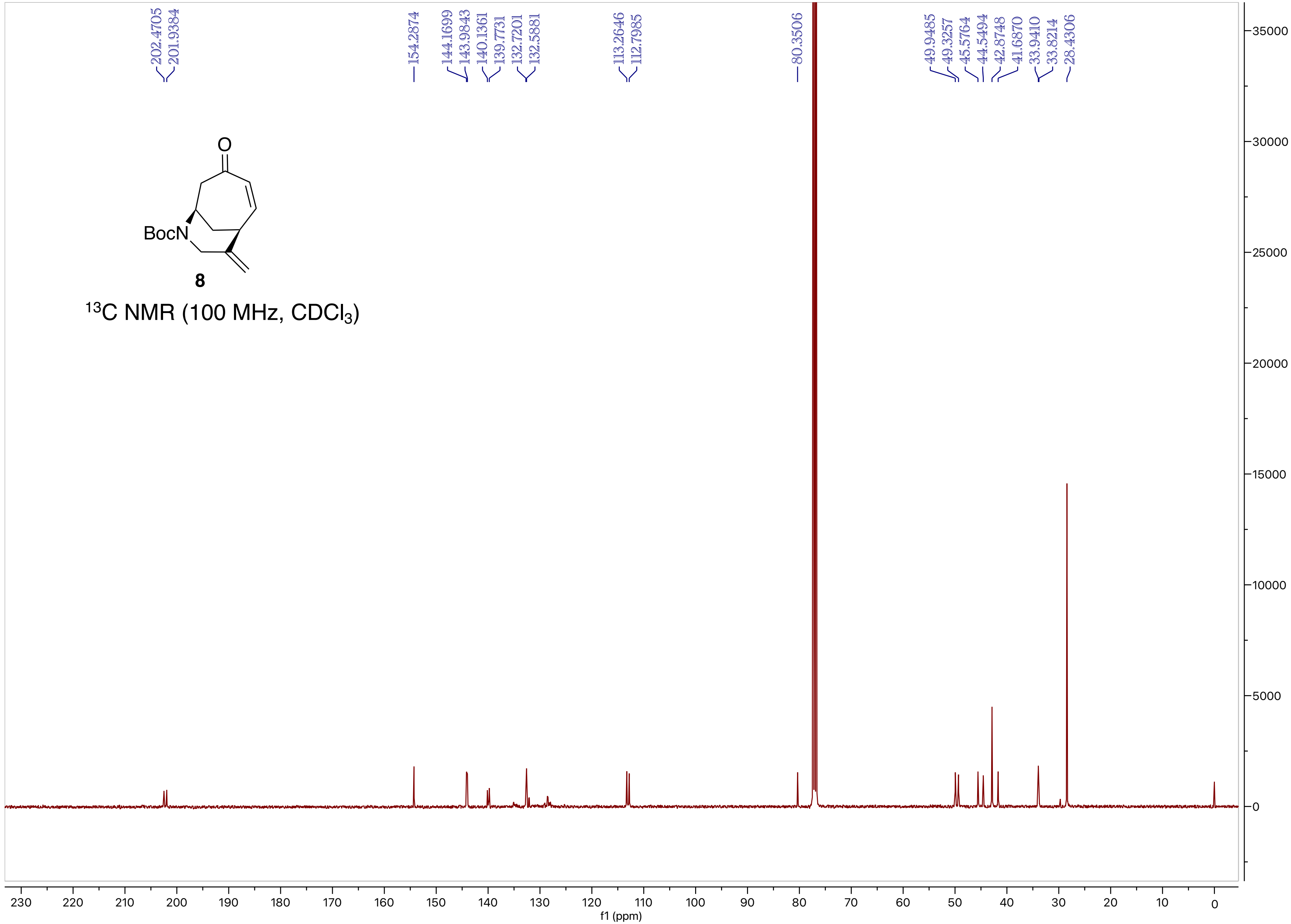
202.4705
201.9384
154.2874
144.1699
143.9843
140.1361
139.7731
132.7201
132.5881
113.2646
112.7985
80.3506
49.9485
49.3257
45.5764
44.5494
42.8748
41.6870
33.9410
33.8214
28.4306
O
BocN
8
^{13}C NMR (100 MHz, $CDCl_3$)
f1 (ppm)

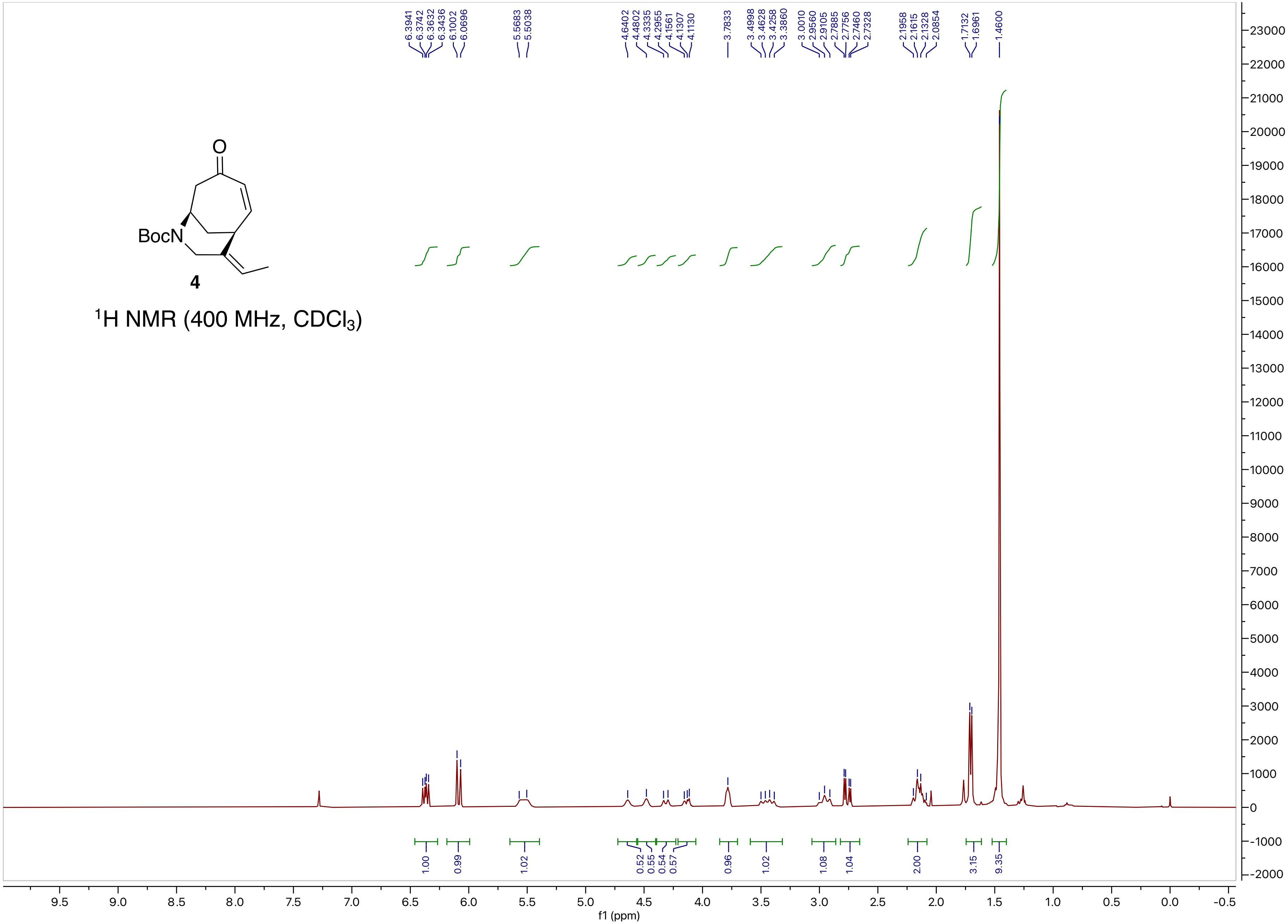

O
BocN
4
1H NMR (400 MHz, CDCl3)
6.3941
6.3742
6.3632
6.3436
6.1002
6.0696
5.5683
5.5038
4.6402
4.4802
4.3335
4.2955
4.1561
4.1307
4.1130
3.7833
3.4998
3.4628
3.4258
3.3860
3.0010
2.9560
2.9105
2.7885
2.7756
2.7460
2.7328
2.1958
2.1615
2.1328
2.0854
1.7132
1.6961
1.4600
1.00
0.99
1.02
0.52
0.55
0.54
0.57
0.96
1.02
1.08
1.04
2.00
3.15
9.35
f1 (ppm)

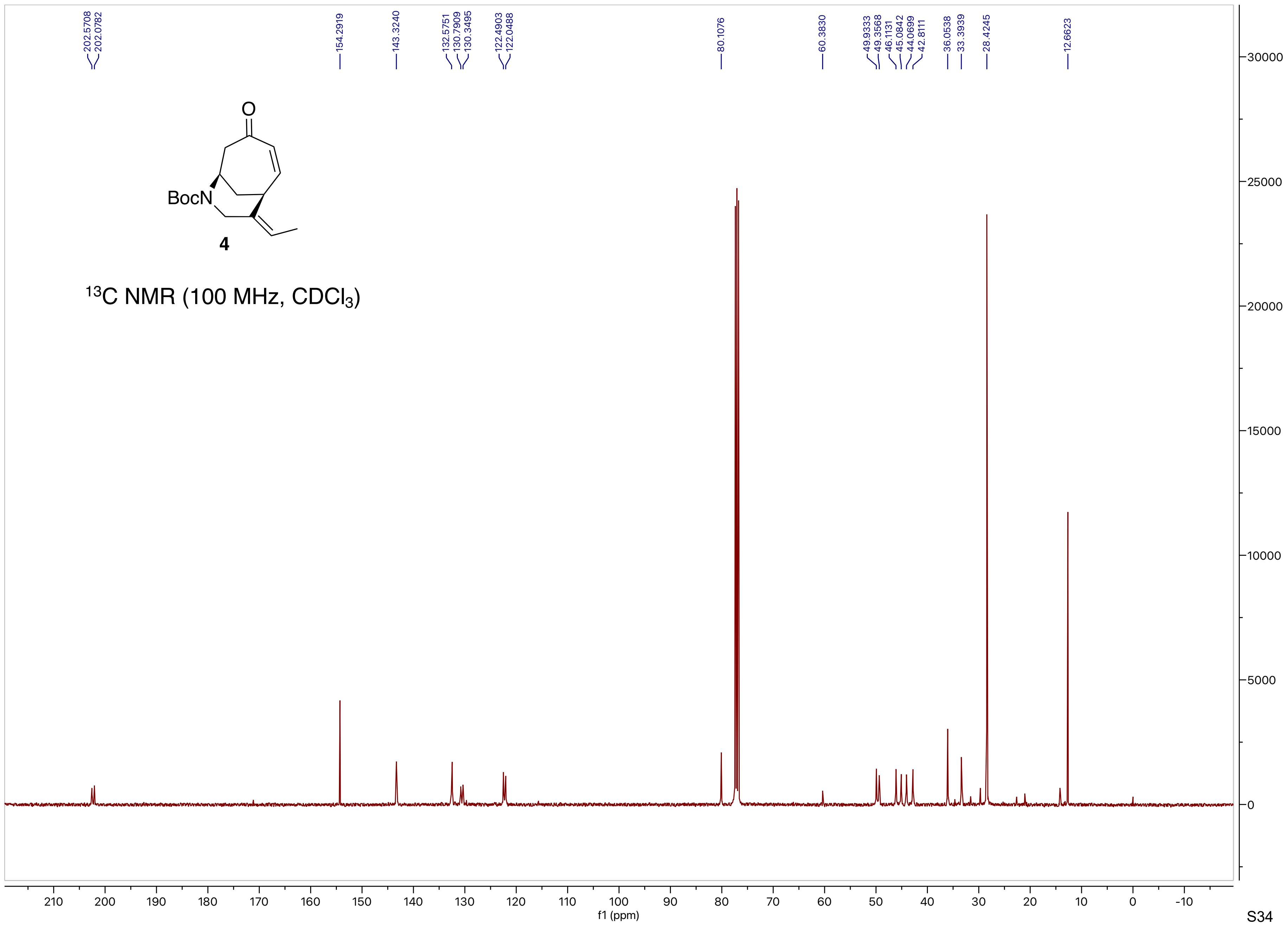
202.5708
202.0782
154.2919
143.3240
132.5751
130.7909
130.3495
122.4903
122.0488
80.1076
60.3830
49.9333
49.3568
46.1131
45.0842
44.0699
42.8111
36.0538
33.3939
28.4245
12.6623
O
BocN
4
^{13}C NMR (100 MHz, $CDCl_3$)
f1 (ppm)

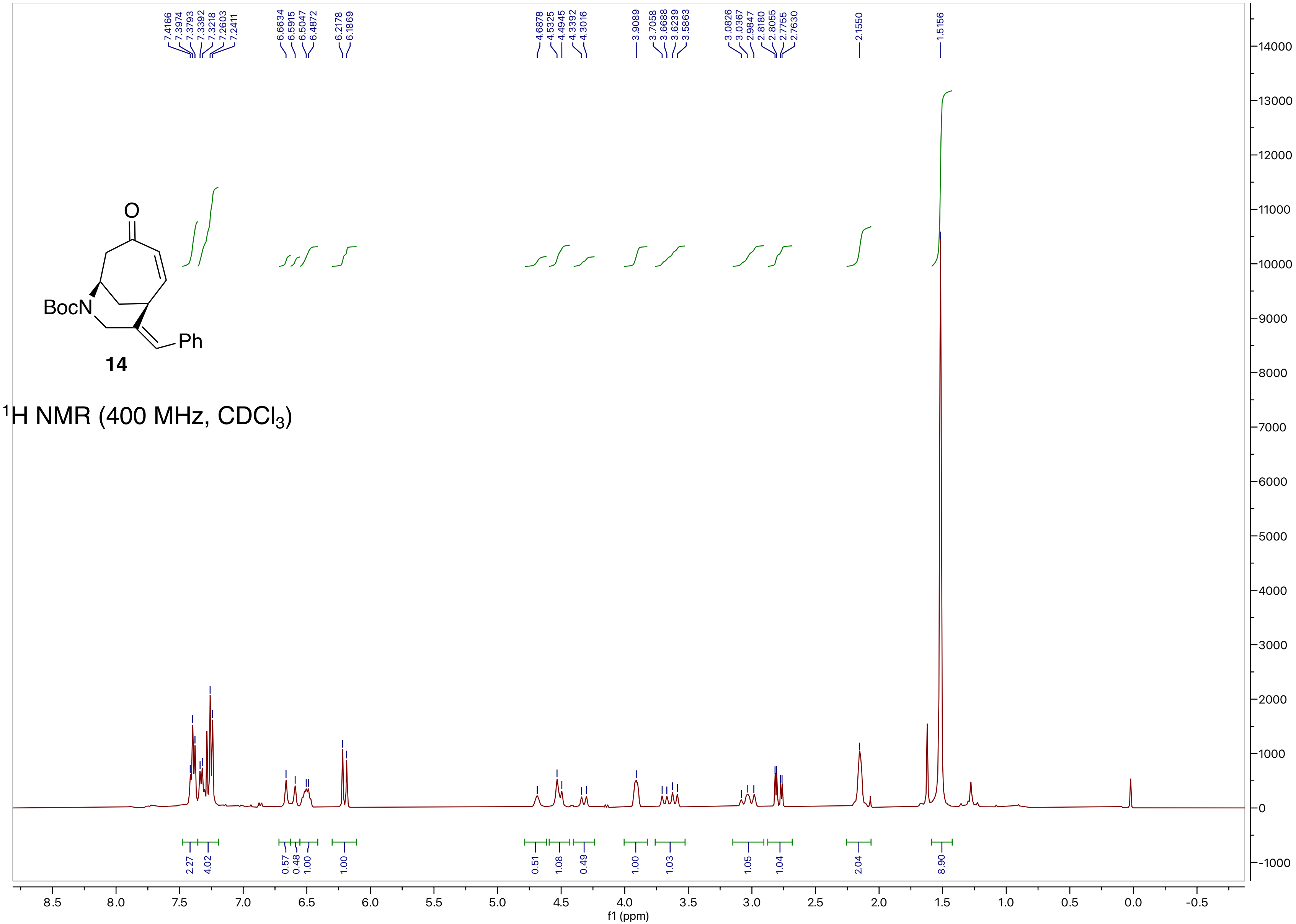
7.4166
7.3974
7.3793
7.3392
7.3218
7.2603
7.2411
6.6634
6.5915
6.5047
6.4872
6.2178
6.1869
4.6878
4.5325
4.4945
4.3392
4.3016
3.9089
3.7058
3.6688
3.6239
3.5863
3.0826
3.0367
2.9847
2.8180
2.8055
2.7755
2.7630
2.1550
1.5156
O
BocN
Ph
14
^{1}H NMR (400 MHz, CDCl$_3$)
2.27
4.02
0.57
0.48
1.00
1.00
0.51
1.08
0.49
1.00
1.03
1.05
1.04
2.04
8.90
f1 (ppm)

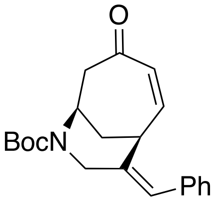

14

^{13}C NMR (100 MHz, $CDCl_3$)

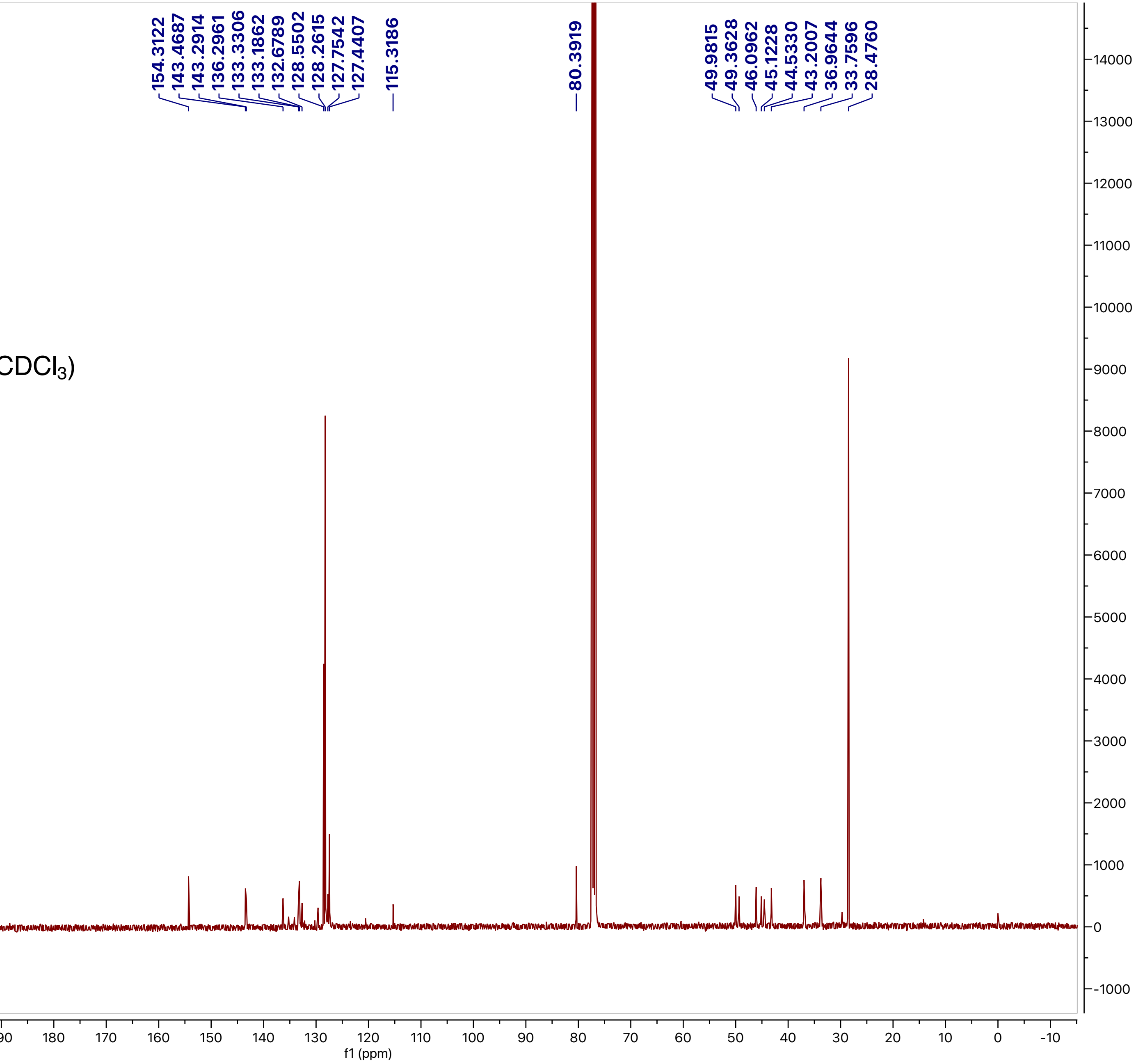

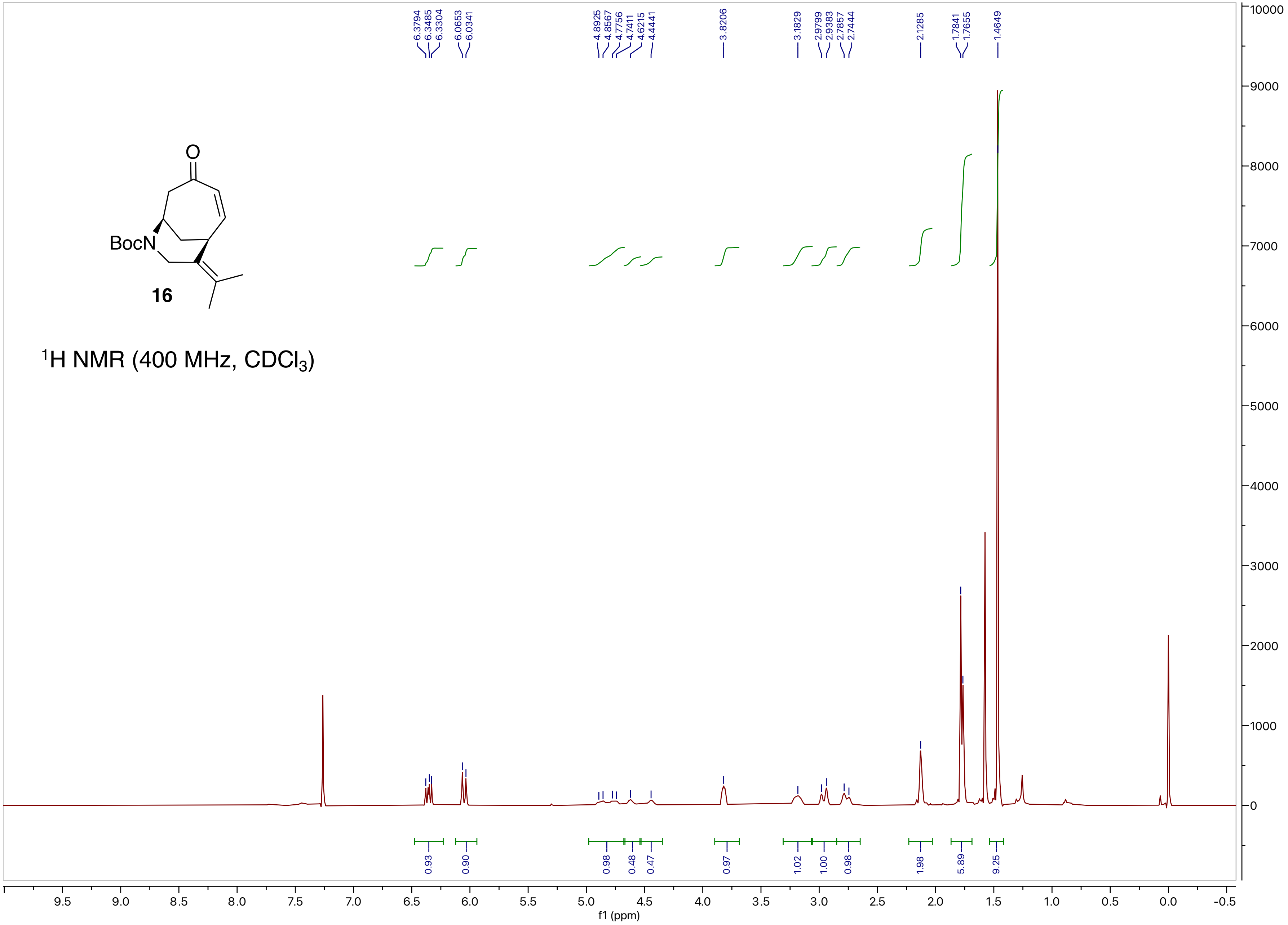
6.3794
6.3485
6.3304
6.0653
6.0341
4.8925
4.8567
4.7756
4.7411
4.6215
4.4441
3.8206
3.1829
2.9799
2.9383
2.7857
2.7444
2.1285
1.7841
1.7655
1.4649
O
BocN
16
^{1}H NMR (400 MHz, $CDCl_3$)
0.93
0.90
0.98
0.48
0.47
0.97
1.02
1.00
0.98
1.98
5.89
9.25
f1 (ppm)

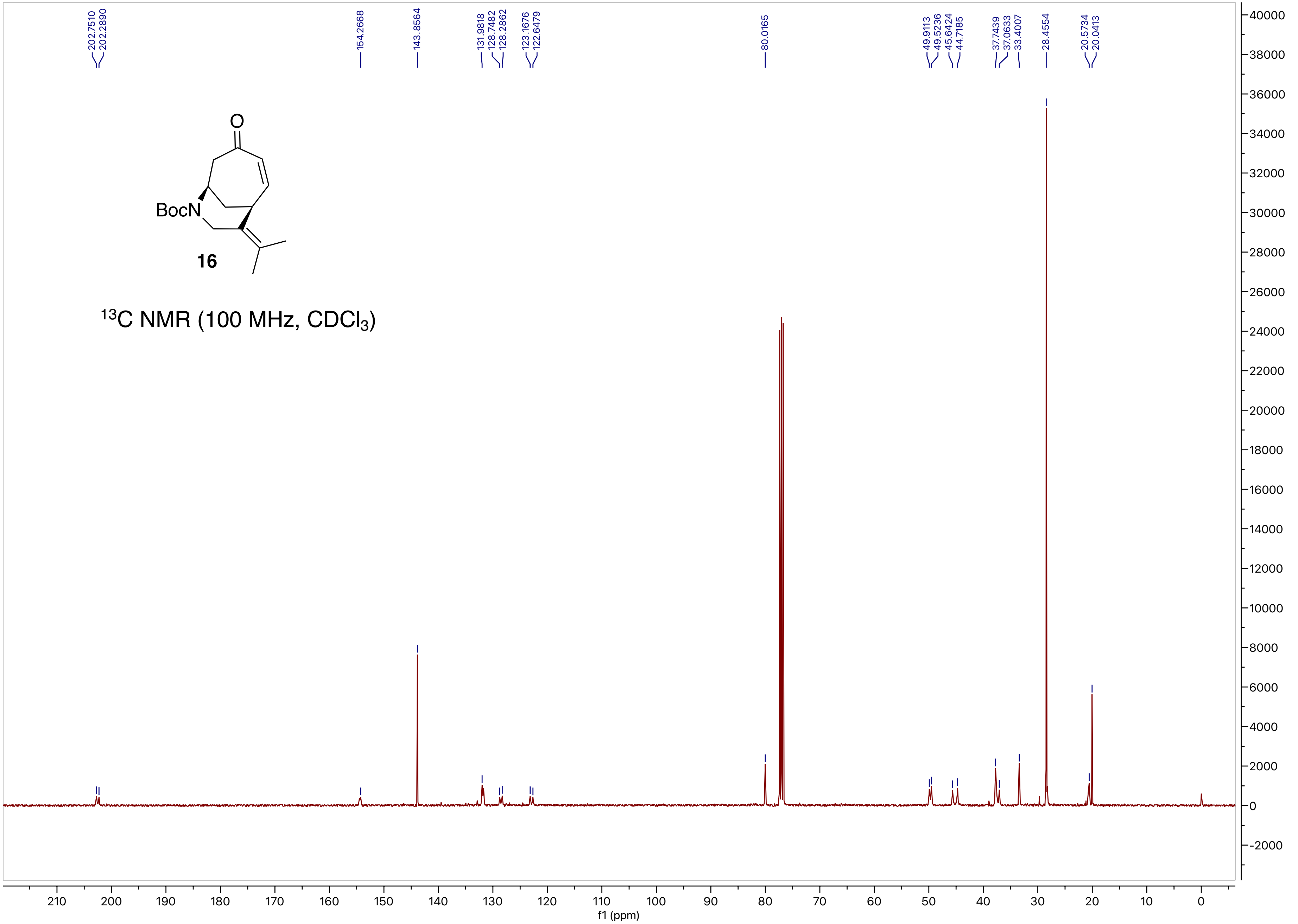
202.7510
202.2890
154.2668
143.8564
131.9818
128.7482
128.2862
123.1676
122.6479
80.0165
49.9113
49.5236
45.6424
44.7185
37.7439
37.0633
33.4007
28.4554
20.5734
20.0413
O
BocN
16
13C NMR (100 MHz, CDCl3)
f1 (ppm)

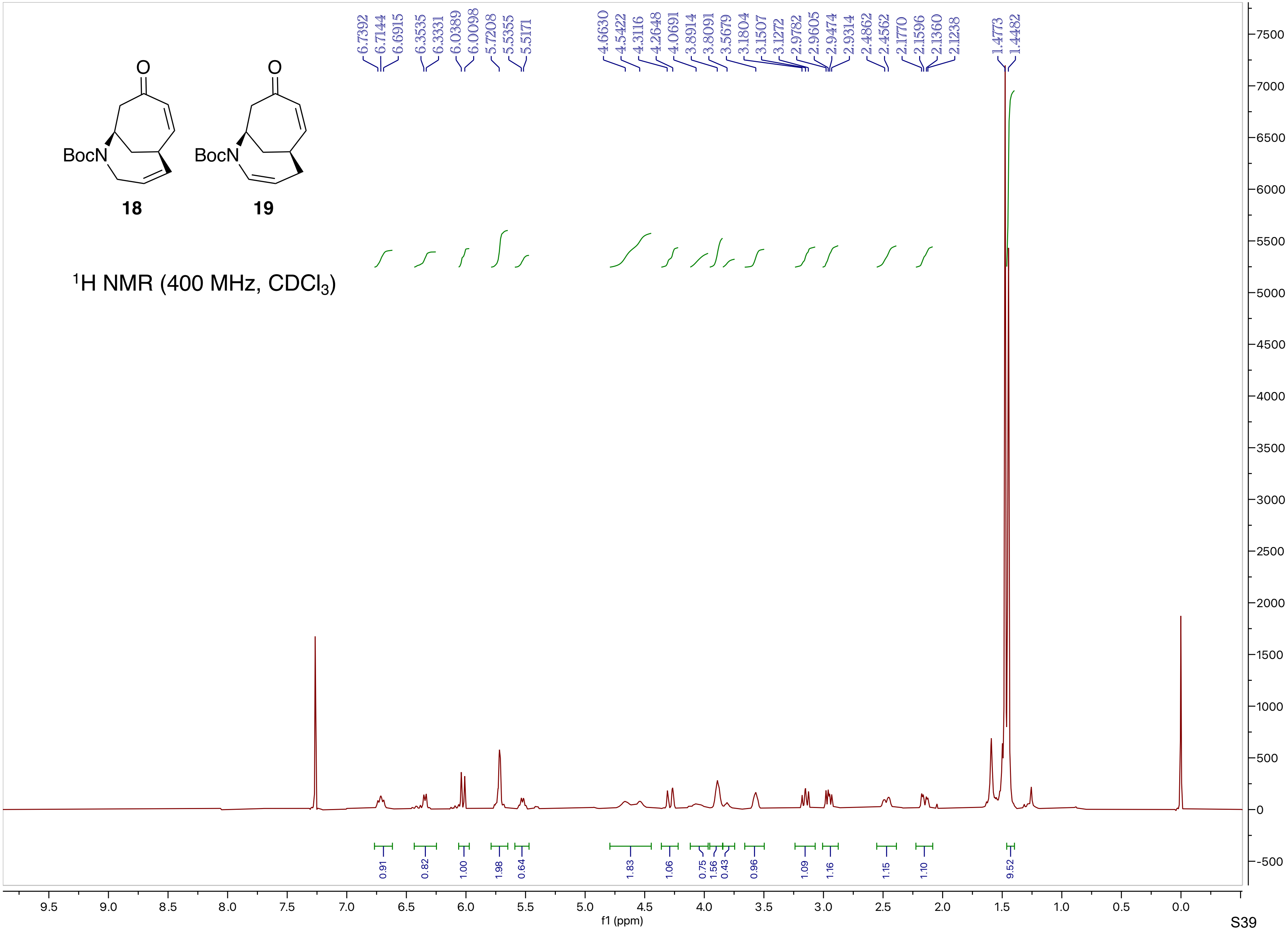
6.7392
6.7144
6.6915
6.3535
6.3331
6.0389
6.0098
5.7208
5.5355
5.5171
4.6630
4.5422
4.3116
4.2648
4.0691
3.8914
3.8091
3.5679
3.1804
3.1507
3.1272
2.9782
2.9605
2.9474
2.9314
2.4862
2.4562
2.1770
2.1596
2.1360
2.1238
1.4773
1.4482
O
BocN
18
19
^{1}H NMR (400 MHz, CDCl$_3$)
0.91
0.82
1.00
1.98
0.64
1.83
1.06
0.75
1.56
0.43
0.96
1.09
1.16
1.15
1.10
9.52
f1 (ppm)

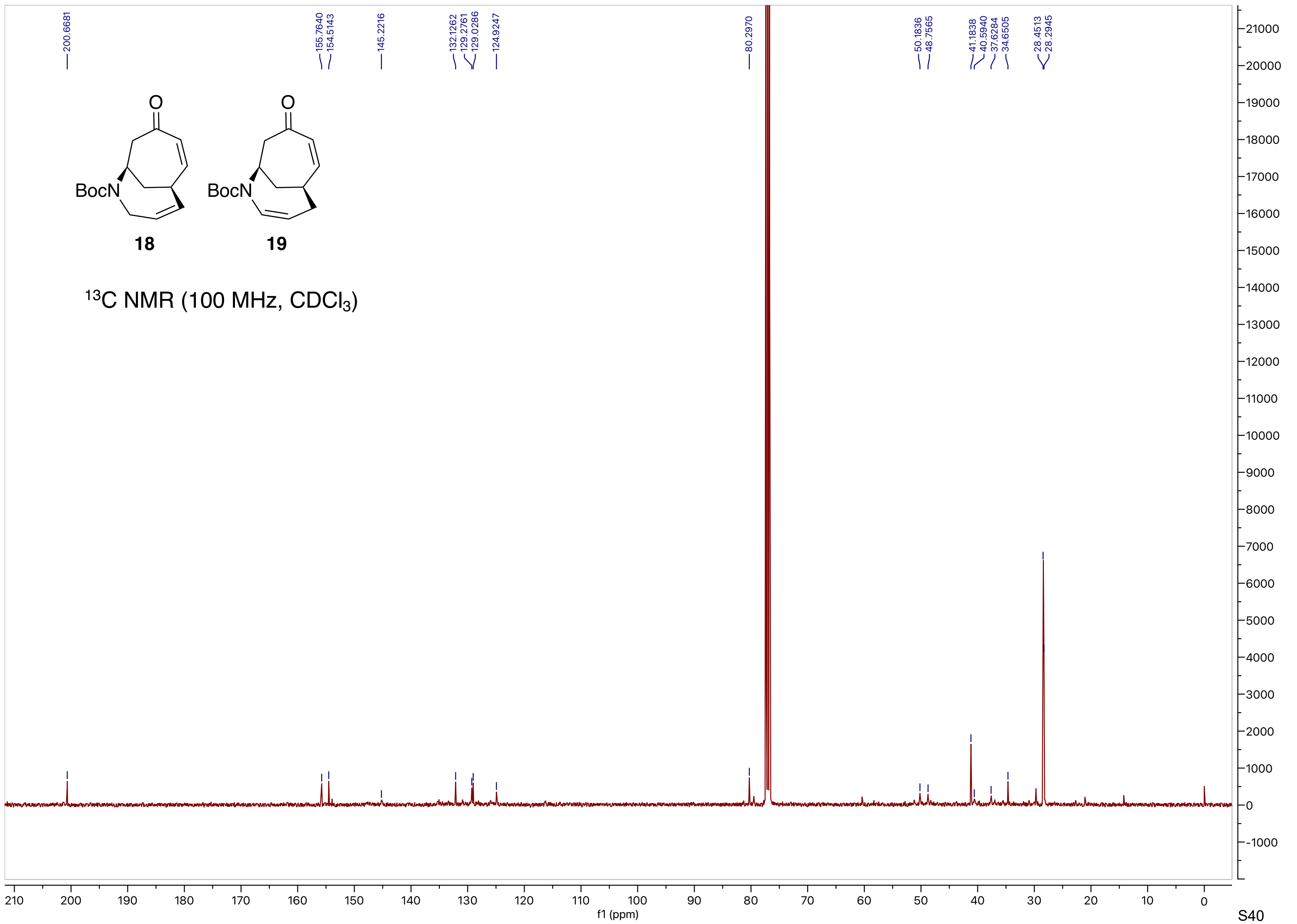
200.6681
155.7640
154.5143
145.2216
132.1262
129.2761
129.0286
124.9247
80.2970
50.1836
48.7565
41.1838
40.5940
37.6284
34.6505
28.4513
28.2945
O
BocN
18
19
^{13}C NMR (100 MHz, CDCl$_3$)
f1 (ppm)

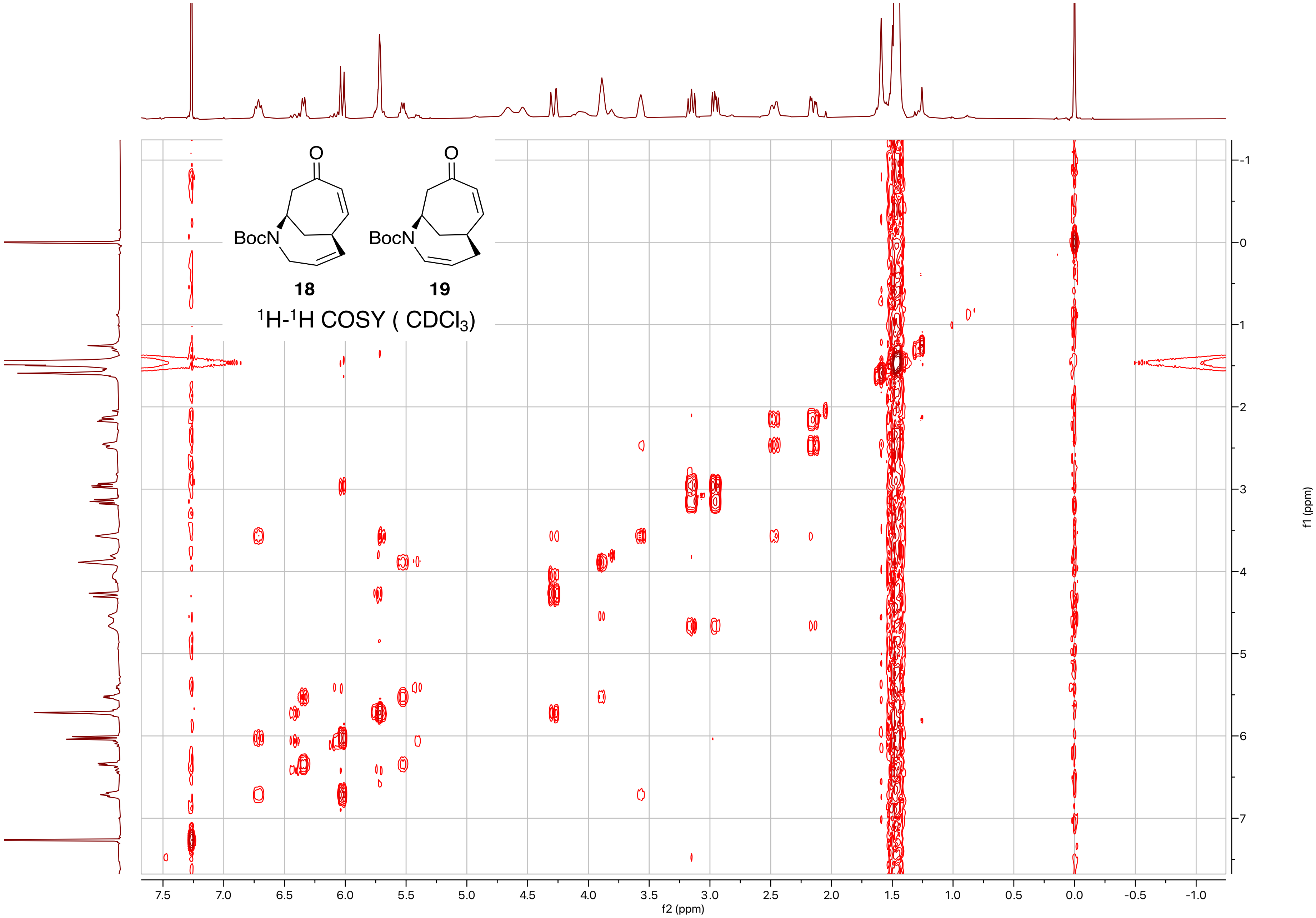
O
O
BocN
BocN
18
19
1H-1H COSY (CDCl3)
f2 (ppm)
f1 (ppm)
7.5 7.0 6.5 6.0 5.5 5.0 4.5 4.0 3.5 3.0 2.5 2.0 1.5 1.0 0.5 0.0 -0.5 -1.0
-1 0 1 2 3 4 5 6 7

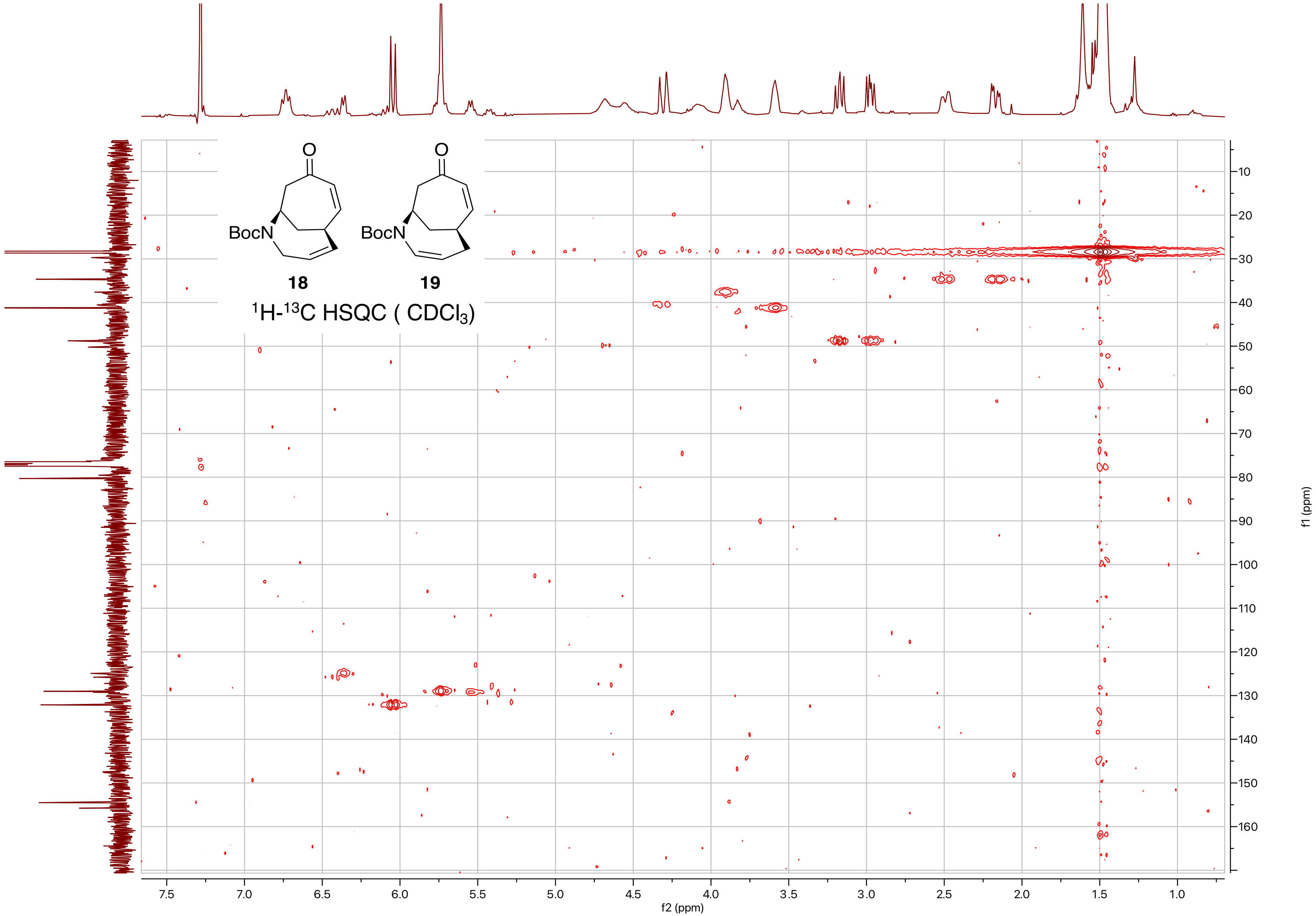

BocN
18
19
^{1}H-^{13}C HSQC ($CDCl_3$)
f2 (ppm)
f1 (ppm)
